# Establishment of a Strong Link Between Smoking and Cancer Pathogenesis through DNA Methylation Analysis

**DOI:** 10.1038/s41598-017-01856-4

**Published:** 2017-05-12

**Authors:** Yunlong Ma, Ming D. Li

**Affiliations:** 1 State Key Laboratory for Diagnosis and Treatment of Infectious Diseases, Collaborative Innovation Center for Diagnosis and Treatment of Infectious Diseases, The First Affiliated Hospital, Zhejiang University School of Medicine, Hangzhou, China; 2 Research Center for Air Pollution and Health, Zhejiang University, Hangzhou, China; 3 Institute for NeuroImmune Pharmacology, Seton Hall University, South Orange, NJ United States

## Abstract

Smoking is a well-documented risk factor in various cancers, especially lung cancer. In the current study, we tested the hypothesis that abnormal DNAm loci associated with smoking are enriched in genes and pathways that convey a risk of cancer by determining whether smoking-related methylated genes led to enrichment in cancer-related pathways. We analyzed two sets of smoking-related methylated genes from 28 studies originating from blood and buccal samples. By analyzing 320 methylated genes from 26 studies on blood samples (N = 17,675), we found 57 enriched pathways associated with different types of cancer (FDR < 0.05). Of these, 11 were also significantly overrepresented in the 661 methylated genes from two studies of buccal samples (N = 1,002). We further found the aryl hydrocarbon receptor signaling pathway plays an important role in the initiation of smoking-attributable cancer. Finally, we constructed a subnetwork of genes important for smoking-attributable cancer from the 48 non-redundant genes in the 11 oncogenic pathways. Of these, genes such as *DUSP4* and *AKT3* are well documented as being involved in smoking-related lung cancer. In summary, our findings provide robust and systematic evidence in support of smoking’s impact on the epigenome, which may be an important contributor to cancer.

## Introduction

Cigarette smoking is a common adverse behavior resulting in various cancers^1^. Notably, smoking confers a higher risk for lung cancer, on average between 5- and 10-fold. In developed countries, smoking is responsible for more than four of five cases of lung cancer^2^. A recent World Health Organization report^3^ showed that smoking-related deaths worldwide are approximately 6 million annually, of which the main deadly cause is cancer.

More than 60 known carcinogens have been detected in cigarette smoke^4^, which include polycyclic aromatic hydrocarbons (PAHs), nitrosamines, and aromatic amines; all play a crucial role in tumorigenesis^5^. Nicotine *per se* not only is the main addictive compound causing smokers to continue to their habit but also makes a genotoxic contribution to the pathogenesis of cancer^6^. Most of these carcinogenic substances require metabolic activation to form DNA adducts that evoke genetic mutations and epigenetic reprogramming, which have been linked to genomic instability and other alterations^4^.

So far, many genetic association studies have revealed numerous variants underlying smoking-attributable cancers^7–9^. One of the most robust findings in genome-wide association studies is that variants in the *CHRNA5*/*A3*/*B4* cluster on chromosome 15q24-25.1 show a significant association with both nicotine dependence and lung cancer^10^. However, current genetics-based evidence is lacking for elucidating the carcinogenic mechanisms of cigarette smoking-associated cancers, which leads many researchers to focus on the function of smoking-associated DNA methylation (SA-DNAm).

DNA methylation, a reversible and heritable alteration that attaches a methyl group to a nucleotide, influences the expression of a disease by mediating transcriptional regulation of genes^11^, alternative splicing^12^, or the integrity of the genome^13^. Recent studies have demonstrated an important role for changes in DNAm during the earlier stages of carcinogenesis^14, 15^. Furthermore, multiple lines of evidence from candidate gene-specific methylation (GSM) studies^16^ have indicated that aberrant DNAm in the promoter region of susceptibility genes for cigarette smoking confer a risk of cancer.

As high-throughput next-generational sequencing and array platforms emerge, our research approach and concept have been converted from hypothesis-driven exploration to data-driven hypothesis generation^17^. Many epigenome-wide association studies (EWASs) have revealed a greater number of DNAm loci associated significantly with *in utero* effects of either maternal smoking^18^ or smoking in adulthood^19^. Besides, several studies have indicated that sustained exposure to cigarette smoke is an indicator of epigenetic reprogramming at a global level by measuring the methylation of repetitive elements, such as those of Sat2^20^ and LINE-1^21^.

To the best of our knowledge, there has been no study that provides a systematic analysis of these identified SA-DNAm loci with the system biology approach for smoking behavior. Our working hypothesis was that abnormal DNAm loci associated with smoking are enriched in important genes and biological pathways, which convey a risk of the initiation and progression of cancer. The primary objective of this study was to test this hypothesis by determining whether these methylated genes in smokers are indeed enriched in well-documented biological pathways implicated in the etiology of cancer.

## Results

### Genes enriched by SA-DNAm from blood samples

Following the procedure described in Supplementary Figure S1, 28 studies published between 2008 and 2015 were identified, which included 9 candidate GSM studies and 19 EWASs (N = 18,677 subjects; Supplementary Table S1). Of them, 26 studies were from 17,675 blood samples. For the blood samples, 320 SA-DNAm-enriched genes with at least two independent pieces of evidence were included for the pathway-based analysis in the discovery stage. A list of the genes from the blood samples is shown in Supplementary Table S2.

### Overrepresented pathways of genes from blood samples

In the discovery stage, we did pathway analysis of 320 genes significantly methylated by smoking, which revealed 90 overrepresented biological pathways with an FDR Q value of <0.05 (Supplementary Table S4). Of these, 57 pathways were reported to be associated with the etiology of cancer (Supplementary Table S5). For example, the most significant pathway of “MSP-RON signaling” (FDR Q value = 2.2 × 10^−4^; see Table 1) has been implicated in regulating the activity of macrophages in response to inflammatory stimuli related to epithelial and leukemic carcinogenesis^22^. The second significant one, “RAR activation,” was overrepresented by 12 identified genes (FDR Q value = 3.7 × 10^−4^) and has been prominently associated with the development of cancer^23^.

**Table 1:** Overrepresented Pathways Underlying Smoking-Attributable Cancer from Blood Samples (FDR < 0.01).

Canonical Pathway	No. of Genes	P value	FDR
MSP-RON signaling pathway	8	6.17 × 10^−07^	0.00022
RAR activation	14	2.04 × 10^−06^	0.00037
Rac signaling	10	6.17 × 10^−06^	0.00071
Actin cytoskeleton signaling	14	7.94 × 10^−06^	0.00071
Aryl hydrocarbon receptor signaling	11	1.15 × 10^−05^	0.00083
Signaling by Rho family GTPases	14	2.51 × 10^−05^	0.0015
AMPK signaling	12	2.951 × 10^−05^	0.0016
Renin-angiotensin signaling	9	6.03 × 10^−05^	0.0028
Molecular mechanisms of cancer	17	7.41 × 10^−05^	0.0030
CXCR4 signaling	10	0.00017	0.0058
ERK/MAPK signaling	11	0.00021	0.0058
HER-2 signaling in breast cancer	7	0.00021	0.0058
Thrombin signaling	11	0.00022	0.0058
HGF signaling	8	0.00027	0.0060
Relaxin signaling	9	0.00028	0.0060
Role of tissue factor in cancer	8	0.00033	0.0063
Non-small cell lung cancer signaling	6	0.00060	0.0096

Furthermore, some of these overrepresented pathways cause vulnerability to a specific type of cancer (Supplementary Table S5), such as the pathways of “non-small cell lung cancer signaling” (FDR Q value = 9.6 × 10^−3^), “small cell lung cancer signaling” (FDR Q value = 0.012), “pancreatic adenocarcinoma signaling” (FDR Q value = 0.017), “renal cell carcinoma signaling” (FDR Q value = 0.026), “ovarian cancer signaling” (FDR Q value = 0.026), and “prostate cancer signaling” (FDR Q value = 0.041). In addition, many other overrepresented pathways are involved in the oncogenic process of various cancers, which include “actin cytoskeleton signaling” (FDR Q value = 7.1 × 10^−4^), “signaling by rho family GTPases” (FDR Q value = 1.5 × 10^−3^), “AMPK signalling” (FDR Q value = 1.6 × 10^−3^), and “ERK/MAPK signaling” (FDR Q value = 5.8 × 10^−3^) (Supplementary Table S5).

### Common molecular pathways in blood and buccal samples

To validate the findings from blood samples, we conducted a similar pathway-based analysis for significantly methylated genes from the buccal samples, which revealed 32 common pathways in the two kinds of samples (P < 0.05; Supplementary Table S6). Among them, 11 pathways were associated with cancer (Table 2), including “RAR activation,” “actin cytoskeleton signaling,” “aryl hydrocarbon receptor signaling,” “signaling by rho family GTPases,” and “molecular mechanisms of cancer.” This provides evidence that these pathways are highly likely to contribute to the pathogenesis of smoking-attributable cancer.

**Table 2:** Eleven Overrepresented Cancer-Related Pathways in Both Blood and Buccal Samples.

Canonical Pathway	Discovery Sample (blood)			Validation Sample (buccal)	
	No. of Genes	P value	FDR	No. of Genes	P value
RAR activation	14	2.04 × 10^−06^	0.00037	13	0.008
Actin cytoskeleton signaling	14	7.94 × 10^−06^	0.0007	13	0.019
Aryl hydrocarbon receptor signaling	11	1.15 × 10^−05^	0.0008	11	0.004
Signaling by Rho family GTPases	14	2.51 × 10^−05^	0.002	13	0.039
Molecular mechanisms of cancer	17	7.41 × 10^−05^	0.003	28	1.55 × 10^−05^
G-protein coupled receptor signaling	12	8.51 × 10^−04^	0.012	17	0.004
PTEN signaling	7	0.003	0.021	9	0.014
Axonal guidance signaling	15	0.004	0.025	22	0.020
Colorectal cancer metastasis signaling	10	0.004	0.025	13	0.036
GNRH signaling	7	0.005	0.025	9	0.021
Breast cancer regulation by stathmin1	8	0.012	0.049	12	0.020

Note: The cut-off threshold of discovery samples was FDR < 0.05 and that of validation samples was P < 0.05.

**Figure 1:**
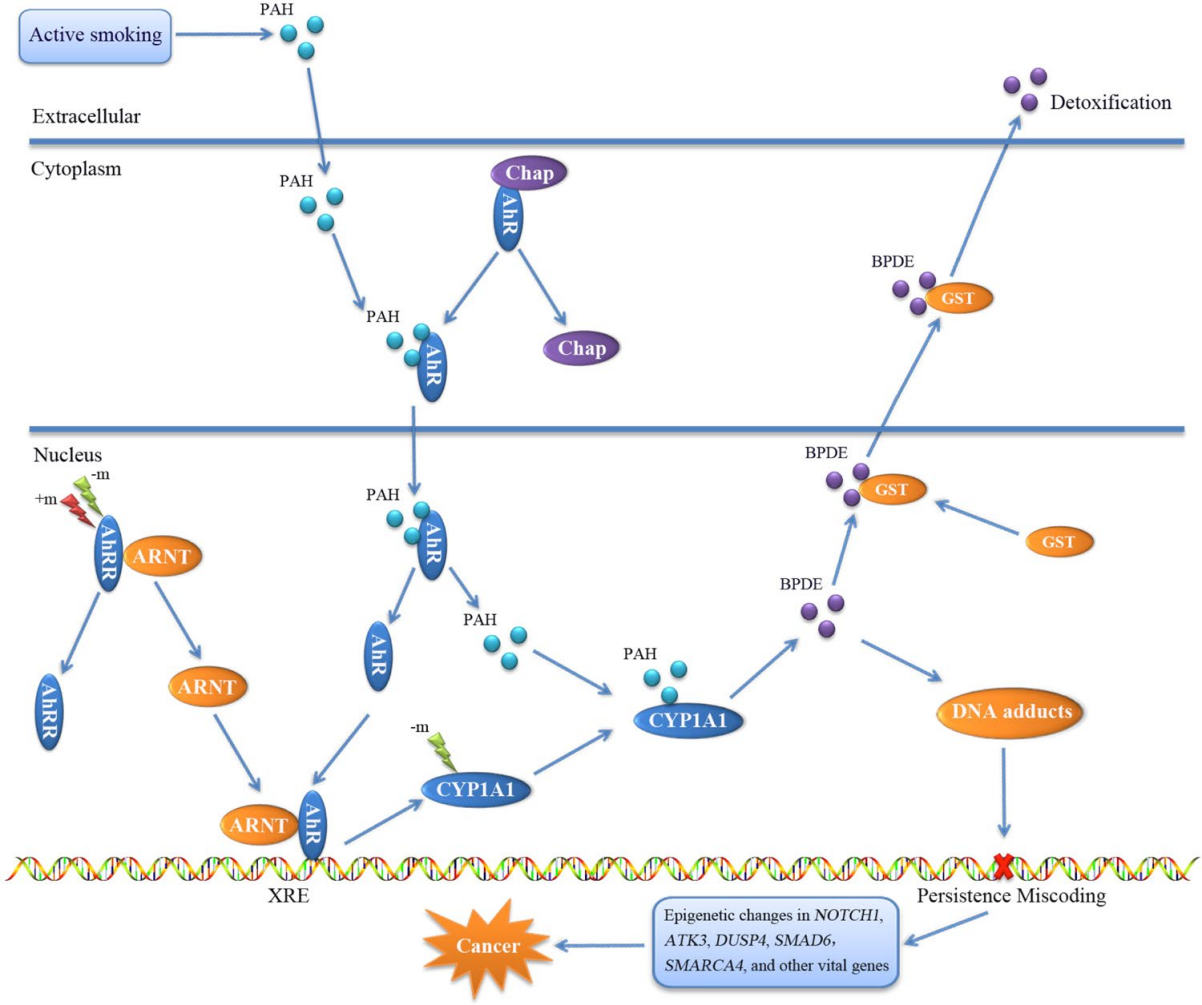
The pathway of “aryl hydrocarbon receptor signaling”-initiated smoking-related cancer. Arrows show event flow. –m represents hypomethylation, and +m represents hypermethylation. The plot was generated using Microsoft PowerPoint. Under normal circumstances, toxic substances from cigarette smoke, including PAHs, nitrosamines, and aromatic amines, could enter the bloodstream through the alveolar capillary system and be taken up by pulmonary cells. Toxic chemicals such as the PAHs bind to transcription factor AhR, which results from the dissociation of AhR and an associated chaperone protein (Chap) complex. After translocating to the nucleus, PAHs and AhR dissociate, and AhR is dimerized with ARNT, which is produced from the AhRR–ARNT complex. The resulting complex binds to the XRE in the promoter of *CYP1A1* to enhance the expression of CYP1A1. The CYP1A1 then metabolizes PAHs into hydrophilic intermediates such as B[a]-7,8-dihydrodiol-9,10-epoxide (BPDE), which can be detoxified through the glutathione S-transferase (GST) family of enzymes or, in an alternative manner, form DNA adducts. Under abnormal circumstances, *CYP1A1* is -m or *AhRR* has altered methylation (−m or +m) that may extraordinarily enhance the expression of CYP1A1, which could induce more DNA adduct formation that results in miscoding of the DNA sequence. Under long-term smoking exposure, the DNA sequence suffers persistent miscoding that triggers epigenetic changes in many critical cancer genes, such as *NOTCH1*, *ATK3*, *DUSP4*, *SMAD6*, and *SMARCA4*.

**Figure 2:**
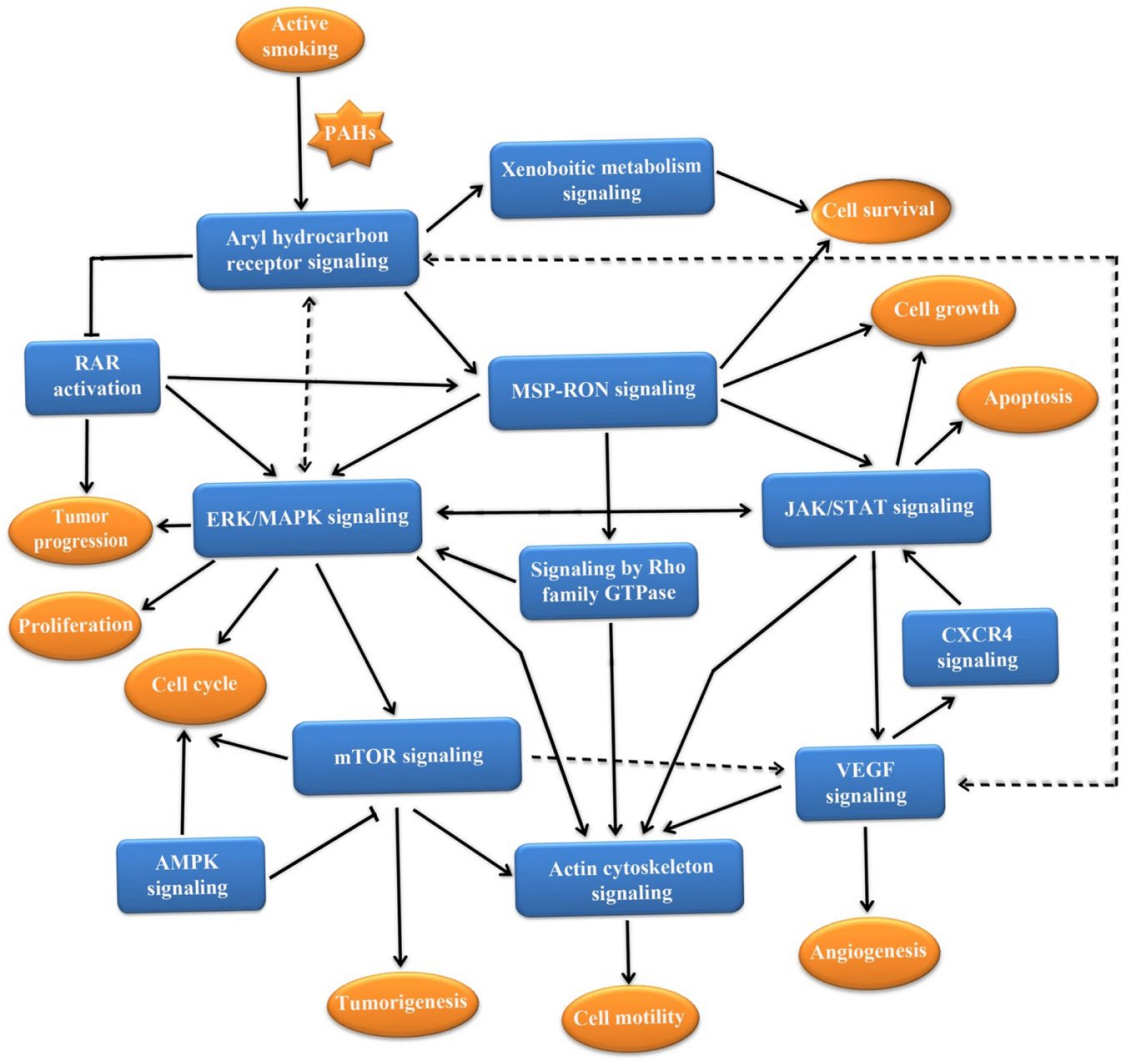
Schematic representation of the major enriched pathways underlying smoking-attributable cancers. Accumulating evidence indicates that smoking prominently induces cancer development. Based on the DNAm-enriched genes associated with smoking, we identified various overrepresented pathways. The major pathways were then linked on the basis of their biological relations originating from the database of IPA and reported literature. The dashed line representing the link between two pathways was reviewed from the reported literature. The plot is generated using Microsoft PowerPoint.

Interestingly, various crucial cancer-related genes, such as *AHRR*, *CYP1A1*, *TNF*, *SMARCA4*, *CDK6*, *RARA*, *RXRB*, *CDKN1A*, *RARG*, and *NFE2L2*, were enriched in the “aryl hydrocarbon receptor signaling pathway” (Supplementary Table S5), through which abnormal epigenetic programming may trigger smoking-attributable cancer (Fig. 1). Figure 2 presents a schematic model of major oncogenic pathways underlying the molecular mechanism of smoking-attributable cancer.

Similar to pathway analysis, we did a GO analysis for those significantly methylated genes from both blood and buccal samples. In the blood sample, we found 19 enriched categories of molecular functions, with an FDR Q value < 0.05 (Supplementary Table S7). The most significantly enriched gene set was “transcription activator activity,” with an enrichment of 3.22 (FDR Q value = 1.92 × 10^−4^). The second most significant one was “sequence-specific DNA binding,” with an enrichment of 2.73 (FDR Q value = 1.92 × 10^−4^). Seven categories of molecular functions were detected in the buccal samples as well (Table 3).

**Table 3:** Gene Ontology (GO) Analysis Reveals Common Molecular Functions of Genes from Both Blood and Buccal Samples.

GO-ID	Molecular Function	Blood Sample			Buccal Sample		
		No. of Genes	P value	FDR	No. of Genes	P value	FDR
0043565	Sequence-specific DNA binding	30	6.20 × 10^−07^	0.00019	43	1.91 × 10^−05^	2.15 × 10^−03^
0005515	Protein binding	178	2.63 × 10^−06^	0.00054	325	5.54 × 10^−05^	5.47 × 10^−03^
0030528	Transcription regulator activity	48	2.54 × 10^−05^	0.0026	88	5.93 × 10^−07^	2.34 × 10^−04^
0005488	Binding	239	4.26 × 10^−05^	0.0038	466	5.79 × 10^−06^	9.14 × 10^−04^
0003700	Transcription factor activity	30	1.12 × 10^−03^	0.032	62	9.90 × 10^−07^	2.57 × 10^−04^
0008092	Cytoskeletal protein binding	19	1.42 × 10^−03^	0.038	37	1.72 × 10^−05^	2.15 × 10^−03^
0019899	Enzyme binding	22	1.61 × 10^−03^	0.038	38	7.14 × 10^−04^	4.70 × 10^−02^

To gain insights from the pathological viewpoint, we did disease-focused enrichment analysis on those genes significantly methylated by smoking in both blood and buccal cells. The most significantly enriched disease was cancer (Supplementary Figure S2). This again indicates that many of these genes methylated by smoking are indeed correlated with cancer.

### Subnetwork constructed from the 11 common cancer-related pathways

**Figure 3:**
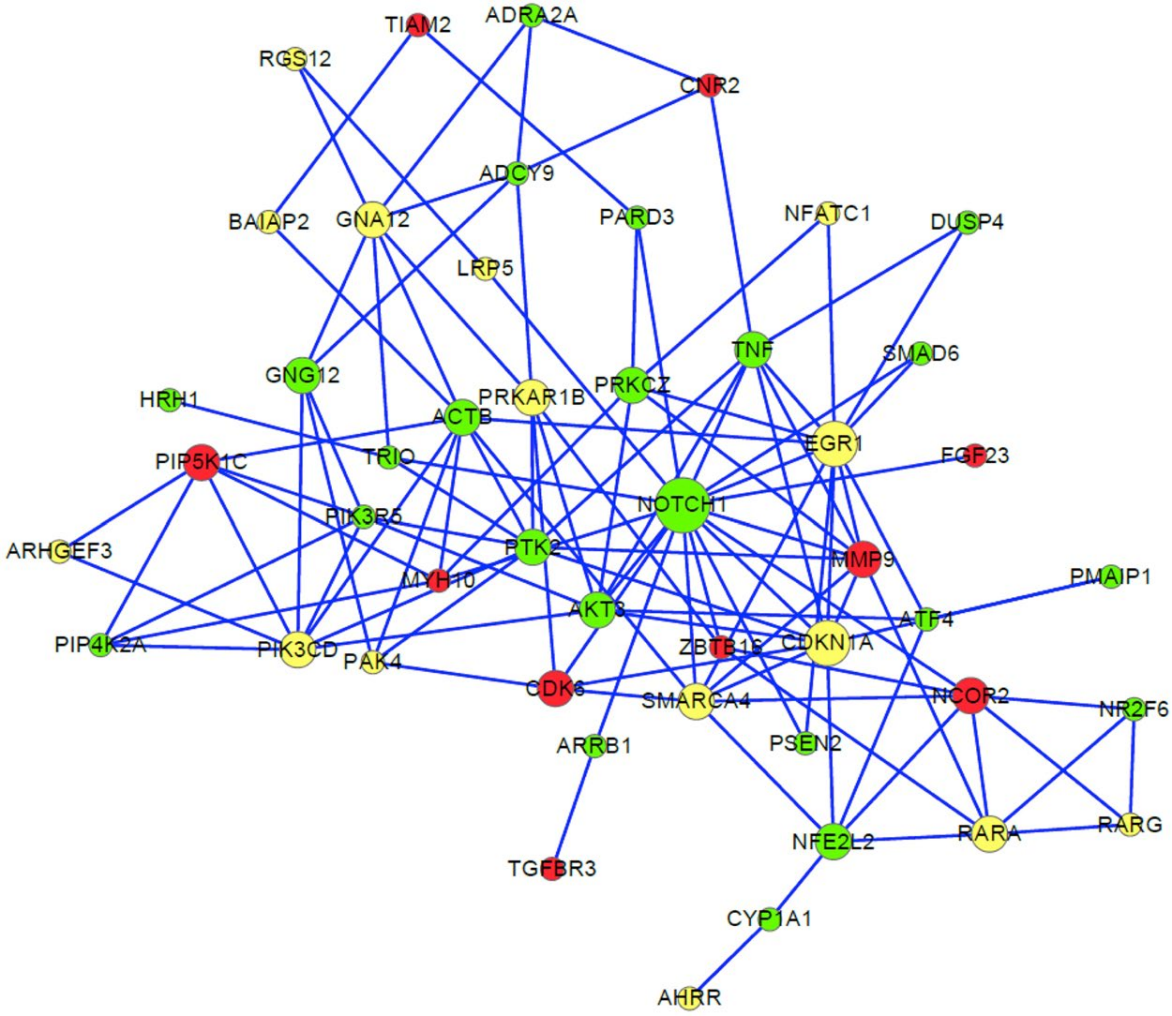
Gene subnetwork constituted by genes from the 11 common oncogenic pathways. The protein–protein interactions were based on the database of STRING v 10.0. We used Cytoscape software to visualize the subnetwork. The color of a node indicates the methylation direction of CpG loci in a gene. Red = hypermethylation, green = hypomethylation, and yellow = both hyper- and hypomethylation at different sites. The edges of the genes represent predicted functional links. The number of edges in each gene was used for determining the node size, of which *NOTCH1* is the biggest.

Considering the presence of a significant number of overlapping genes among the 11 common pathways, we selected 48 non-redundant genes based on their biological functions and appearance frequencies among the common pathways and used them to construct a cancer-associated molecular subnetwork (Fig. 3). The well-documented cancer-related genes *NOTCH1*, *CDKN1A*, *EGR1*, *AKT3*, *TNF*, *MMP9*, and *SMARCA4* are located in the center of this newly constructed subnetwork (Fig. 3).

### 48 smoking-related methylated genes contribute to lung cancer

**Figure 4:**
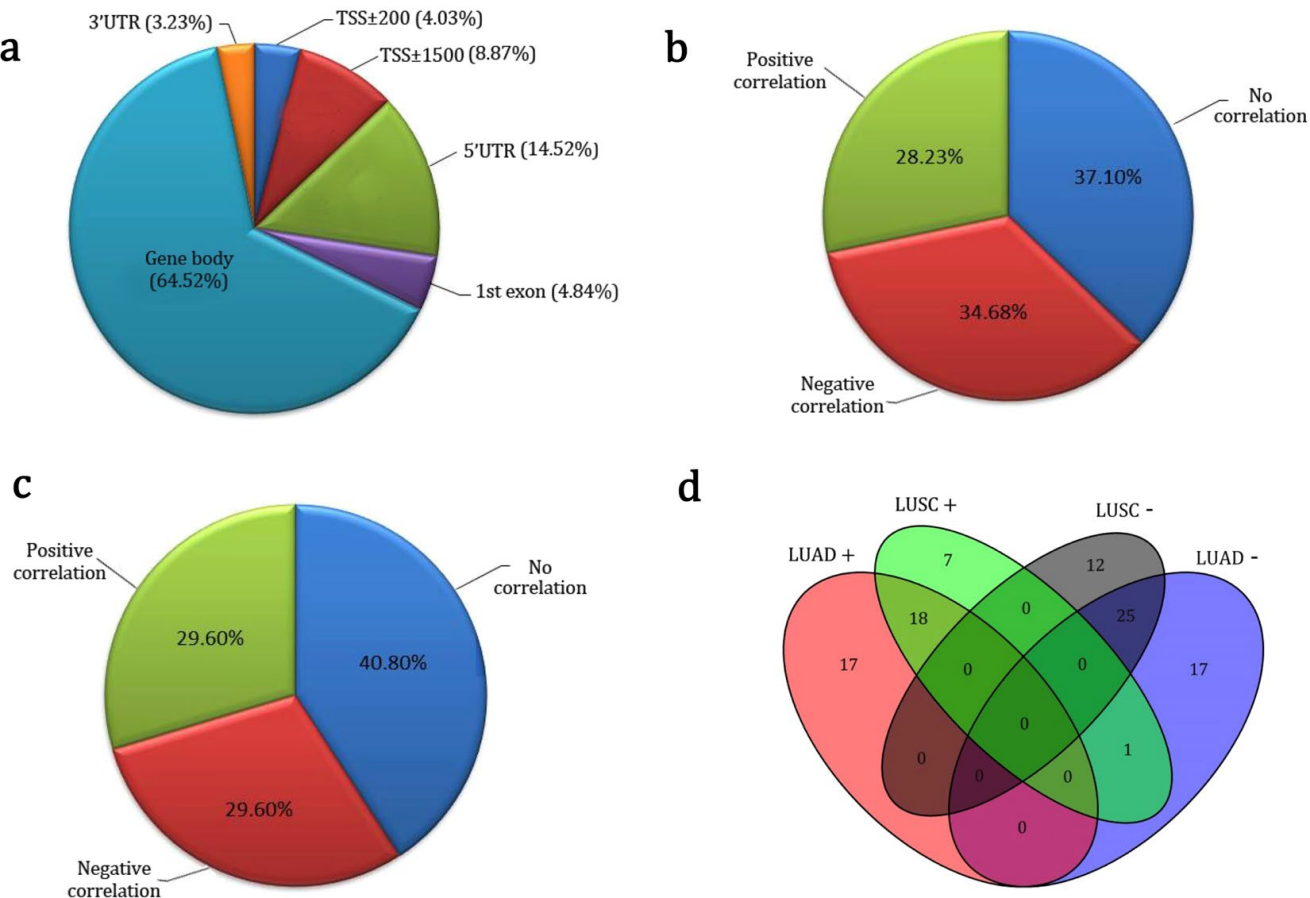
Methylation loci of the 48 identified genes. (**a**) Proportion of methylation loci in different regions. (**b**) Proportion of methylation loci that showed no, positive, or negative correlation with RNA expression in LUAD samples. (**c**) Proportion of methylation loci that showed no, positive, or negative correlation with RNA expression in LUSC samples. (**d**) Venn diagram shows that many methylation loci correlate consistently with the degree of expression of the associated gene in both LUAD and LUSC.

To gain further evidence of the contribution of the 48 methylated genes to cancer, we investigated the relation between RNA expression and methylation for the genes in the TCGA dataset. Among these genes, we found 148 methylation sites in different regions, with the largest number located in the gene body and 5′UTR (Fig. 4a). After examining the correlation between methylation loci and RNA expression in lung adenocarcinoma (LUAD) and lung squamous-cell carcinoma (LUSC) samples, we found that large portions of the methylation loci were significantly positively or negatively correlated with RNA expression in both LUAD (Fig. 4b and Supplementary Tables S8 and S9) and LUSC (Fig. 4c and Supplementary Tables S10 and S11). Most of the methylation loci correlated with RNA expression were located in the gene body and 5′-UTR in both LUAD (Supplementary Figure S3a,b) and LUSC (Supplementary Figure S3c,d).

**Figure 5:**
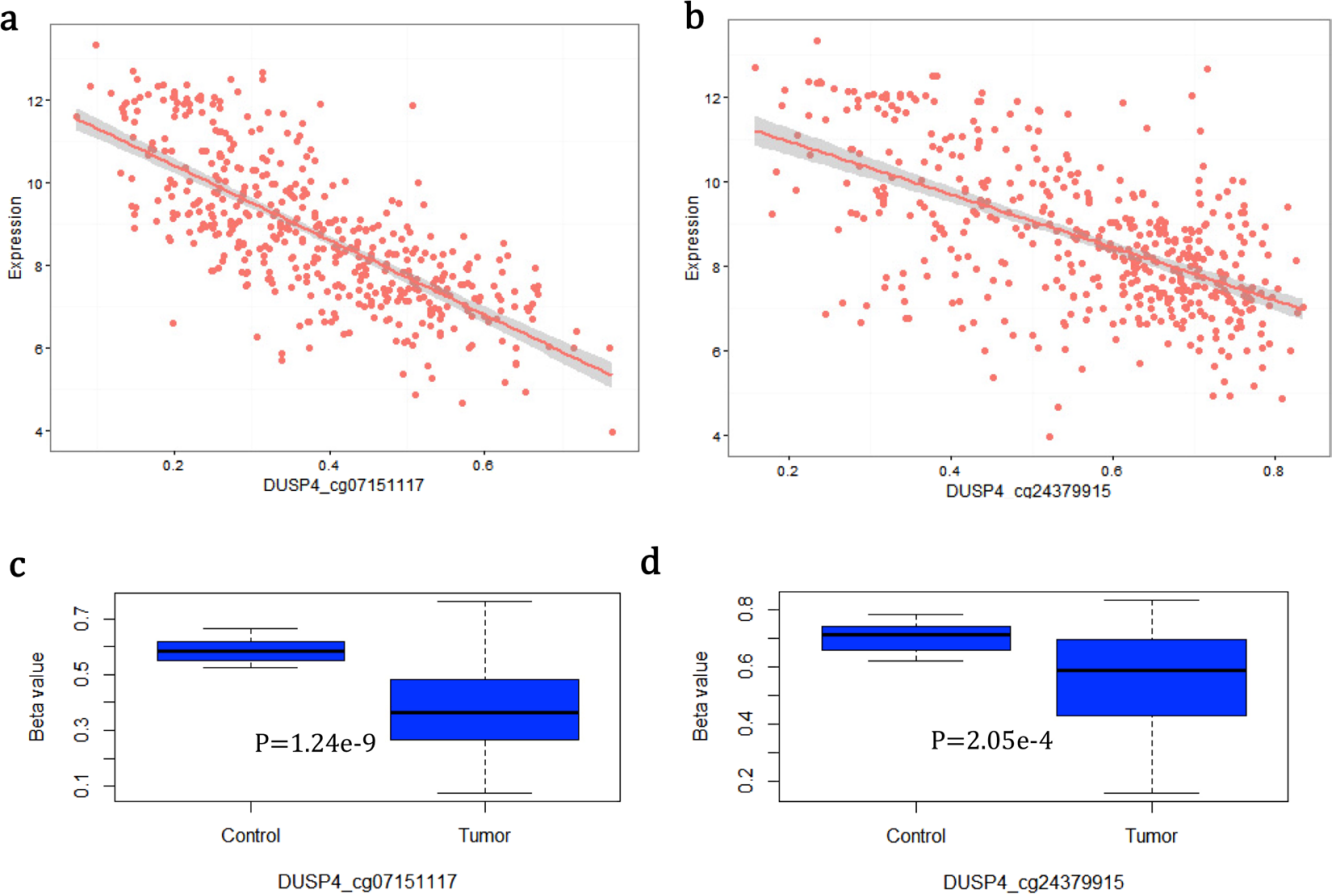
Two methylation probes of *DUSP4* in LUAD samples. (**a**) Correlation of cg07151117 probe with RNA expression in control and cancer cells. (**b**) Correlation of cg24379915 probe with RNA expression in control and cancer cells. (**c**) Extent of methylation of cg07151117 probe in control and cancer cells. (**d**) Extent of methylation of cg24379915 probe in control and cancer cells. P value was calculated by the Wilcoxon-rank sum test.

Interestingly, the majority of methylation loci correlated with the expression of the associated genes in both the LUAD and LUSC samples showed consistent directions (Fig. 4d). There were 18 methylation probes showing a positive correlation with RNA expression in both LUAD (51.4%) and LUSC (69.2%), and 25 methylation probes showing negative correlation with RNA expression in both LUAD (58.1%) and LUSC (67.6%). For example, the cg07151117 probe located in the 5′-UTR of *DUSP4*, the cg27514333 probe located in the gene body of *SMAD6*, and the cg26271591 probe located in the 5′-UTR of *NFE2L2* correlated in a significantly negatively way with RNA expression in both LUAD (Table 4, Fig. 5a,b, and Supplementary Figure S4a,b) and LUSC (Table 4 and Supplementary Figures S5a,b and S6a,b), and the cg11314684 probe in the gene body of *AKT3*, the cg02385153 probe in the gene body of *AHRR*, and the cg24538512 probe in the gene body of *NFATC1* were significantly positively correlated with RNA expression in both LUAD (Table 4 and Supplementary Figure S4c,d) and LUSC (Table 4 and Supplementary Figure S5c,d).

**Table 4:** Top-Ranked Negative and Positive Correlation between Methylation and RNA Expression in Lung Adenocarcinoma (LUAD) and Lung Squamous-Cell Carcinoma (LUSC).

CpG Locus	Chromosome: Position	Gene Region	Gene Name	Correlation Coefficient (r)	P value	Cancer Type
cg07151117	8: 29204954	5′UTR, body	DUSP4	−0.742	<0.001	LUAD
cg24379915	8: 29202958	Body	DUSP4	−0.657	<0.001	
cg27514333	15: 66996626	Body	SMAD6	−0.422	<0.001	
cg04265051	11: 68079686	TSS +/− 1500	LRP5	−0.396	<0.001	
cg04813697	10: 22920025	Body	PIP4K2A	−0.395	<0.001	
cg24538512	18: 77233465	Body	NFATC1	0.503	<0.001	
cg05944967	18: 77166811	5′UTR, body	NFATC1	0.459	<0.001	
cg02385153	5: 404766	Body	AHRR	0.442	<0.001	
cg11314684	1: 244006288	Body	AKT3	0.404	<0.001	
cg10841124	5: 433274	Body	AHRR	0.367	<0.001	
cg26271591	2: 178125956	5′UTR, body	NFE2L2	−0.544	<0.001	LUSC
cg07151117	8: 29204954	5′UTR, body	DUSP4	−0.485	<0.001	
cg27514333	15: 66996626	Body	SMAD6	−0.460	<0.001	
cg19572487	17: 38476024	5′UTR	RARA	−0.407	<0.001	
cg10062919	17: 38503802	Body	RARA	−0.407	<0.001	
cg11314684	1:244006288	Body	AKT3	0.422	<0.001	
cg03604011	5: 400201	Body	AHRR	0.334	<0.001	
cg11902777	5: 368843	Body	AHRR	0.324	<0.001	
cg26850624	5: 429559	Body	AHRR	0.323	<0.001	
cg07805542	1: 9779309	Body	PIK3CD	0.311	<0.001

**Figure 6:**
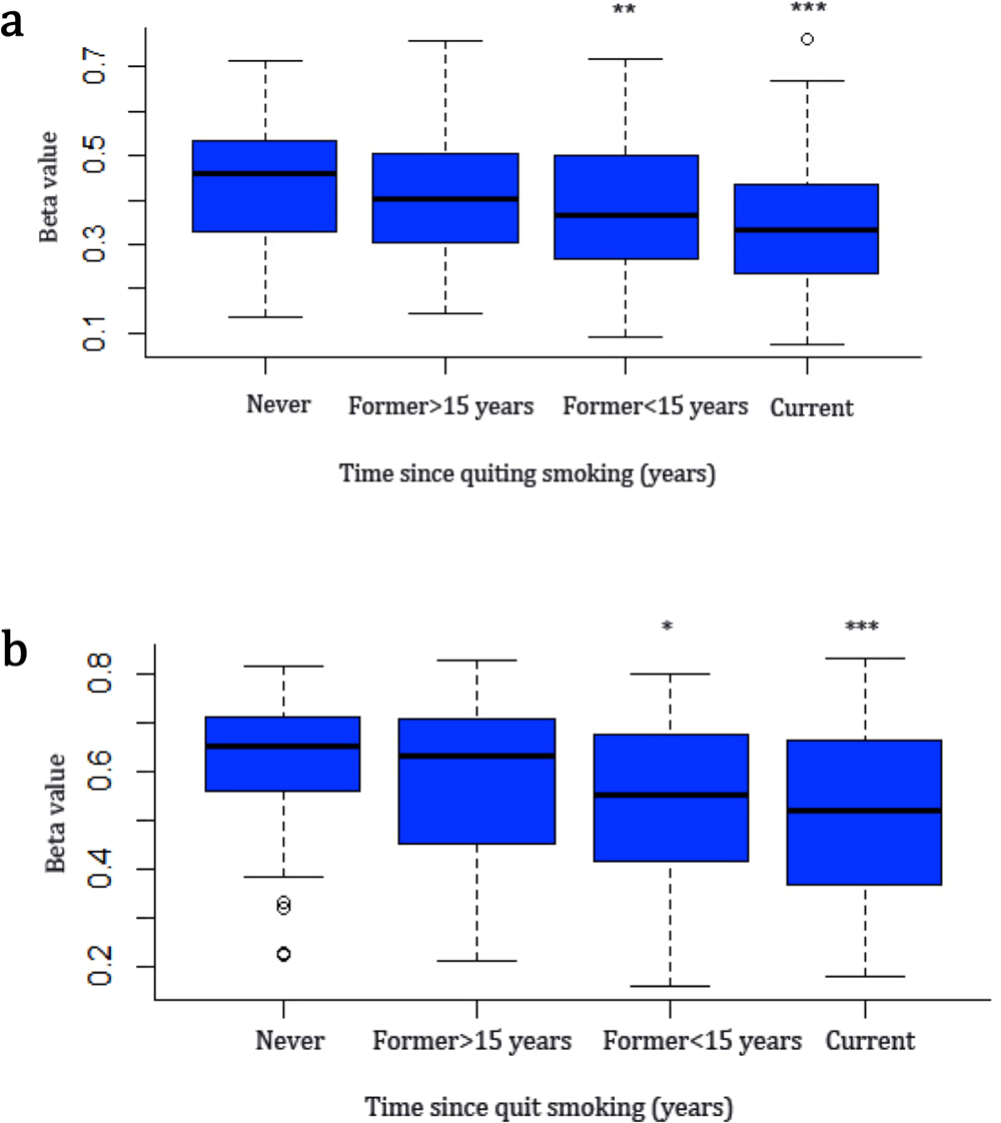
Associations between smoking and methylation of *DUSP4* in LUAD samples. (**a**) Methylation probe of cg07151117. (**b**) Methylation probe of cg24379915. *P < 0.05, **P < 0.01, and ***P < 0.001.

On the other hand, we found that most of the methylation loci that correlated with RNA expression were significantly differentially expressed in the control tissues vs. cancer in both LUAD and LUSC samples (Supplementary Table S12 and Supplementary Figures S7 and S8). This is especially true for *DUSP4*. There were two methylation probes (cg07151117 and cg24379915) of this gene showing significant correlation with RNA expression in both LUAD (Table 4 and Fig. 5a,b) and LUSC (Table 4 and Supplementary Figure S6a,b). The cg07151117 probe showed the strongest inverse correlation between methylation and expression in LUAD samples (r = −0.742; P < 0.001; see Table 4 and Fig. 5a). The cg24379915 probe was negatively correlated with *DUSP4* expression in the LUAD samples (r = −0.657; P < 0.001; see Table 4 and Fig. 5b). Compared with normal tissues, there were two hypomethylation probes of *DUSP4* in cancer tissues (Fig. 5c,d and Supplementary Figure S6c,d). Consistently, the associations of smoking with the two methylation probes of *DUSP4* in LUAD samples (Fig. 6a and b) were in line with the finding that these two CpG loci of *DUSP4* tended to be hypomethylated in smokers, as found by previous EWASs^24, 25^.

## Discussion

In recent years, many studies have emphasized the association of current smoking with DNAm, which is considered a critical mediating factor in the pathogenesis of cancer. In light of epidemiologic evidence indicating that cigarette smoking is highly correlated with cancer, we performed a systematic bioinformatics analysis with the goal of revealing the underlying mechanism of smoking-attributable cancer from an epigenetic point of view, which revealed a group of genes and pathways implicated in the pathology of interest. Based on the findings from the current study and previous biological evidence, we present a schematic model for elucidating the biological effects of smoking on cancer pathogenesis (Fig. 2).

There are two types of studies used to discern the association between smoking and DNAm: candidate GSM and EWAS. For candidate GSM studies, only a limited number of CpG sites mapped to a candidate gene of interest can be investigated. In contrast, a significant number of CpG sites can be studied with EWASs^24–26^. Although EWAS is powerful for identifying novel methylated CpG sites, many confounding factors remain unresolved. For example, in light of the tens of thousands of CpG sites that could be analyzed simultaneously in an EWAS, a significant proportion of reported studies might not have had a large enough sample to decrease the rate of false-positive associations evoked by multiple testing. Further, the presence of epigenetic and genetic heterogeneity and multiple interacting genes can limit the identification of the underlying molecular mechanism of complex diseases. Thus, pathway-based analysis is useful not only for reducing the influence of false-positive findings but also to collaborate the reported genes statistically based on particular biological functions to uncover the meaningful networks conveying the risk of smoking-induced cancer. In the current study, although we used three bioinformatics tools (i.e., IPA, EnrichNet, and GeneTrail) based on different databases to conduct the pathway-based analysis, the main findings were generated by the IPA.

Two independent SA-DNAm-enriched gene sets were extracted from blood and buccal samples. Among the genes from blood samples, many have strong association signals with smoking with multiple replications, such as *AHRR*, *F2RL3*, *AKT3*, and *GFI1*. For example, *AHRR*, a tumor suppressor gene on chromosome 5p15.33, encodes a class E basic helix–loop–helix protein that dampens the translocation of AHR–ligand complex to the nucleus. Knockdown of *AHRR* is correlated with greater tumor cell invasiveness in many tissues, including those of the lung, colon, ovary, and breast^27^. The *F2RL3* protein is related to platelet activation and coagulation, as well as to cell signaling. Epigenetic association studies^28, 29^ have provided consistent evidence that *F2RL3* methylation predisposes to implicatation in lung or colon cancer. By performing a genome-wide methylation analysis, Fasanelli *et al*.^30^ demonstrated that smoking-induced hypomethylation in *AHRR* and *F2RL3* contributes to the risk of lung cancer, providing evidence of specific altered methylation that can mediate the effect of smoking on cancer pathogenesis. Very recently, Joehanes *et al*.^31^ conducted a meta-analysis of genome-wide DNA methylation for the effect of smoking on DNA methylation based on 15,907 blood-derived DNA samples from subjects in 16 cohorts. By comparing current smokers (N = 2,433) with never smokers (N = 6,956), 18,760 CpG sites annotated to 7,201 genes were found to be differentially methylated at a genome-wide false discovery rate (FDR) <0.05. Although these results replicated many previously reported loci, including CpGs annotated to *AHRR*, *RARA*, and *F2RL3*, the authors did not use an independent sample to replicate most of the identified CpG loci. By performing an enrichment analysis for smoking-related phenotypes in the NHGRI-EBI GWAS Catalog, these authors found that these smoking-related methylated genes were significantly overrepresented in all types of cancer (P = 8.0 × 10^−15^), lung adenocarcinoma (P = 1.5 × 10^−3^), and colorectal cancer (P = 1.4 × 10^−3^), which is in line with our findings. In comparison, we found that 95.6% (306/320) of the genes identified in blood samples and 68.7% (454/661) of those in buccal samples overlapped with the genes (N = 7,201) of Joehanes’ study, which offers supportive evidence of the importance of the smoking-related methylated genes used in current study.

By employing a systematic statistical analysis, several intriguing findings emerged from our analyses, which probably never would have been identified in any individual epigenetic association study, including EWAS. Our analysis of methylated genes from blood corroborated the view that many oncogenic pathways were significantly associated with smoking, including non-small-cell lung cancer signaling, small-cell lung cancer signaling, prostate cancer signaling, and renal-cell carcinoma signaling. Furthermore, many other enriched pathways, for example MSP-RON signaling, RAR activation, rac signaling, and actin cytoskeleton signaling, which have been associated with the etiology of cancer in previous studies (Supplementary Table S5), were remarkably linked with smoking. For instance, the retinoic acid receptors (RARs) have potent anti-proliferative and anti-inflammatory properties, suppressing the activity of transcription factors AP-1 and NF-κB. Our findings thus suggest that abnormalities in the pathway of “RAR activation” confer susceptibility to cancer. Recently, Guilhamon *et al*.^32^ reported that the “RAR activation” pathway is affected by differential methylation in cancers.

To confirm our findings using blood samples, we conducted an independent pathway-based analysis of methylated genes from buccal cells, which validated 11 cancer-related pathways. This confirmation indicates that these common oncogenic pathways play important roles in the pathology of smoking-attributable cancer. Particularly, the pathway of aryl hydrocarbon receptor signaling plays a crucial role in detoxification of the toxic components of cigarette smoke, including PAHs, nitrosamines, and aromatic amines^33^. If there were aberrant modifications in this biological regulation, these toxic substances could directly influence the epigenetic profile of circulating whole blood cells or other tissues. Using mice lacking the aryl hydrocarbon receptor (AhR), several studies^34^ have shown that AhR regulates angiogenesis by activating vascular endothelial growth factor in the endothelium and inactivating tumor growth factor-β in the stroma; both are important in supporting the proliferation of tumor cells by supplying nutrients and oxygen. Together, abnormal smoking-related DNAm in the aryl hydrocarbon receptor signaling pathway may induce more DNA adduct formation that leads to miscoding of the sequence of DNA (see Fig. 1). With long-term smoking exposure, the DNA sequence suffers persistent miscoding that triggers epigenetic changes in various vital oncogenes, such as *NOTCH1*, *ATK3*, *DUSP4*, *SMAD6*, and S*MARCA4*, in the major enriched pathways (see Fig. 2) and leads to carcinogenesis, indicating that the aryl hydrocarbon receptor signaling pathway probably is implicated in the initiation of smoking-induced cancers.

Because pathway-based analysis cannot identify genes that work across different pathways, network analysis has been widely used to search for groups of functionally related genes that may collectively convey susceptibility to diseases such as cancer. In addition, because abnormal methylation may be implicated in cancer development through regulation of gene expression, we explored whether the smoking-associated methylation loci were correlated with RNA expression of genes identified in LUAD and LUSC. Thus, by using the web-based tool STRING^35^, we offer a subnetwork for the 48 non-redundant genes among the 11 common oncogenic pathways. Of note, 47 of the 48 genes (97.9%) in the subnetwork overlapped with the genes mapped by smoking-related CpG loci at a genome-wide FDR < 0.05 in Joehanes’s study^31^. Many of the 48 genes play essential roles and have been implicated in a variety of cancers. For example, the hub gene of *NOTCH1*, encoding one of the four Notch receptors, has an important role in a signaling pathway that is involved in multifaceted regulation of cell survival, proliferation, tumor angiogenesis, and metastasis^36^. A substantial body of research shows that *NOTCH1* is correlated with the pathology of cancer^37^. By cross-talking with many other critical cancer genes and pathways, *NOTCH1* plays a fundamental role in cancer pathogenesis. Aberrant methylation of *NOTCH1* may thus lead to a greater risk of smoking-induced cancer. Besides, the SWI/ShNF chromatin-remodeling complex, which has been linked to lung, pancreas, breast, and colon cancer^38^, is comprised of a catalytic subunit of either *SMARCA4* or *SMARCA2*. The product of *SMARCA4* modulates gene expression by using the energy of ATP hydrolysis to modify chromatin structure. Both DNA mutation and methylation influence the expression of *SMARCA4* in cancers such as Burkitt lymphoma^39^, ovarian carcinoma^40^, and lung cancer^41^. Consistently, two methylation loci (cg18040892 and cg23963476) were significantly inversely correlated with RNA expression of *SMARCA4* in LUSC samples. The extent of methylation of the cg23963476 probe, which is hypomethylated in smokers^25^, was significantly lower in LUSC tissues than in control tissues, suggesting that smoking-associated hypomethylation of *SMARCA4* elicits the development of lung cancer.

Furthermore, the *DUSP4* gene, which interacts with the hub genes *TNF* and *EGR1*, plays an important role in the subnetwork of 48 genes involved in oncogenesis. *DUSP4*, which belongs to dual-specificity phosphatase (DUSPs) family, regulating the activity and location of MAPKs, is a negative regulator of extracellular-regulated kinase activity and is upregulated in EGFR-mutant lung cancer cell lines compared with K-ras-mutant cells^42^. Coincidently, a group of investigators reported that allelic loss of *DUSP4* led to underexpression of *DUSP4* in EGFR-mutant lung adenocarcinoma^43^. In addition, numerous studies have shown that *DUSP4* acts as a tumor suppressor^44, 45^ or promotes cancer progression^46, 47^ depending on cancer type. In the present study, we found that two smoking-associated methylation probes (cg07151117 and cg24379915) that are correlated with RNA expression of *DUSP4* were significantly hypomethylated in both LUAD and LUSC cancer tissues compared with the control samples. These results indicate that hypomethylated *DUSP4* is involved in smoking-induced lung cancer. Together, our proposed subnetwork of 48 genes is not only enriched for genes associated with cancer but also associates with smoking-attributable cancer.

There are several limitations to the present study. First, a number of human genes are uncharacterized or not mapped to manually curated or computationally predicated pathways. Therefore, the effects of these unique genes cannot be delineated in our pathway-based analysis. Second, smoking-associated or methylation-associated confounding factors, such as alcohol consumption and body mass index, which were not adjusted for in many of the studies we included, may contribute to the heterogeneity. Third, 661 genes were collected from two buccal-based studies with 1,002 subjects, whereas 320 genes were extracted from 26 blood-based studies with a much larger number of 17,675 subjects. This might imply that there were more false-positive methylated genes in buccal-based studies than in blood-based studies. Thus, we used the methylated genes from blood samples more extensively for pathway-based analysis and used the methylated genes from buccal samples only for replication. Finally, because of the limitation of the cross-sectional design-based study, which was adopted by all the studies we examined, we could not determine whether changes in DNAm were direct consequences of smoking or part of its pathology.

In sum, the present study marks one of the first comprehensive pathway-based analyses of the abnormal methylation of DNA in adult smokers. Our findings indicate strongly that cigarette smoking causes prominent alterations in DNAm enriched in numerous genes and biologically meaningful pathways implicated in cancer pathology. This provides strongly and holistically epigenetics-based evidence in support of the carcinogenic effect of smoking on cancer. However, our understanding of the contribution of smoking-related DNAm to cancer pathogenesis is still in an early stage. More studies are warranted to reveal the specific function of aberrant methylation of particular genes in response to smoking in the development of cancer. Such understanding will have clinical implications for the personalized treatment of smoking-attributable cancer.

## Methods

To identify all studies on the association of cigarette smoking with alterations in DNAm, a total of 1,447 studies published prior to June 13, 2015, were retrieved from the PubMed database. The key words used for the search were “smoking,” “smoke,” “tobacco,” “nicotine,” “cigarette,” and “methylation.” All abstracts of these reports were reviewed for potentially eligible papers. We also manually checked the references individually for additional studies not indexed by the PubMed database.

To eliminate or minimize false-positive findings, we narrowed our selection criteria by choosing genes with significant reported associations with smoking. Once a paper met the inclusion criteria, the full text of the article was reviewed to ensure the conclusion was in accordance with the content. After rigorous and systematic screening, 28 epigenetic association studies consisting of 9 candidate GSM studies and 19 EWASs were included, among which 26 studies were conducted on DNA extracted from whole blood and 2 on DNA from buccal cells (Supplementary Table S1).

At first, we used the genes from the blood samples (Supplementary Table S2) to discover the underlying pathways associated with cigarette smoking. To enhance the reliability of our study, we included only those genes whose relevance is supported by at least two independent pieces of evidence (i.e., there are two or more significant CpG loci within a gene or there is only one significant methylation locus in a gene but the finding has been replicated in two or more independent samples). Under the same inclusion criteria, we also extracted an independent list of genes from buccal cells (Supplementary Table S3) to validate the pathways identified from the blood samples.

### Identification and validation of enriched biological pathways

To obtain a comprehensive understanding of the influence of smoking on cancer from an epigenetic perspective, we conducted stepwise pathway-based analyses for the two types of samples using the bioinformatics tools of Ingenuity Pathway Analysis (IPA)^48^, EnrichNet^49^, and Genetrail^50^.

For IPA, the core part is the Ingenuity Pathways Knowledge Base (IPKB), which is a well-organized proprietary database consisting of extensive information on the functions or interactions of each gene or protein. Based on defined biological knowledge, IPA can analyze a user-defined set of genes for molecular functions, canonical pathways, or cellular networks. With the IPA application, the significance of each identified pathway is calculated as follows: (1) the number of input genes mapped to a given pathway in the IPKB database, denoted by *m*; (2) the number of genes included in the pathway, denoted by *M*; (3) the total number of input genes mapped to the IPKB database, denoted by *n*; and (4) the total number of known genes included in the IPKB database, denoted by *N*. The significance of gene enrichment in the canonical pathways then is calculated using a one-tailed Fisher’s exact test^51^. A P value of <0.05 indicates a statistically significant link between the gene and a given pathway. Nevertheless, because many canonical pathways are examined simultaneously, we used the method of Benjamini-Hochberg^52^ to correct for multiple testing.

Two other web-based bioinformatics tools (i.e., EnrichNet and GeneTrail) for pathway analysis depend on popular public databases, such as the Kyoto Encyclopedia of Genes and Genomes (KEGG)^53^, Wiki pathways^54^, and Biocarta pathway^55^. By using overrepresentation analysis, these tools could be applied for identification, prioritization, and analysis of functional associations between user-collected gene sets and specified canonical pathways. Furthermore, we used the Biological Networks Gene Ontology tool (BiNGO; v 2.44)^56^ for Gene Ontology (GO) analysis, where GO terms are significantly overrepresented in a set of genes calculated by the hypergeometric test^57^ (FDR Q value < 0.05). ReViGO with default parameters^58^ was used to remove the redundant GO terms according to the enrichment in molecular functions. After obtaining the common pathways from both blood and buccal samples, we selected the non-redundant genes among the pathways to construct a cancer-associated molecular subnetwork based on the database of STRING v 10.0^35^. We used the software of Cytoscape^59^ to visualize the cancer-associated molecular subnetwork.

We also downloaded level 3 DNA methylation data (i.e., JHC_USC HumanMethylation450K)^60, 61^ and level 3 RNA expression data (i.e., UNC IlluminaHiSeq_RNASeqV2)^60, 61^ on lung adenocarcinoma (LUAD) and lung squamous cell carcinoma (LUSC) from the large-scale database of TCGA^62^ to provide validation for the identified smoking-related oncogenes. The RNA expression data are log-transformed before being utilized for statistical analysis and data visualization. By using the web-based tool of MEXPRESS^63^, which has two main functions of Pearson correlation^64^ and the non-parametric Wilcoxon rank-sum test^65^, we determined whether methylation probes were correlated with the extent of expression of the associated genes in both LUAD and LUSC samples and the different status of methylation loci correlated with RNA expression between control and cancer in LUAD or LUSC samples. The R packages (http://www.r-project.org/), such as VennDiagram^66^ and ggplot2^67^ were utilized for other statistical analyses and data visualization. By using multiple bioinformatics tools based on different databases, we were able to identify the important genes and biologically meaningful pathways contributing to the vulnerability to smoking-attributable cancer.

## Electronic supplementary material

Supplementary PDF Filepdf

## References

[1] Vineis P (2004). Tobacco and cancer: recent epidemiological evidence. Journal of the National Cancer Institute.

[2] CDC (2010). Racial/Ethnic disparities and geographic differences in lung cancer incidence — 38 States and the District of Columbia, 1998–2006. MMWR Morb Mortal Wkly Rep.

[3] WHO. WHO Tobacco Fact sheet N°339 (http://www.who.int/mediacentre/factsheets/fs339/en/) World Health Organization (2014).

[4] Hecht SS (2003). Tobacco carcinogens, their biomarkers and tobacco-induced cancer. Nature reviews. Cancer.

[5] Pfeifer GP (2002). Tobacco smoke carcinogens, DNA damage and p53 mutations in smoking-associated cancers. Oncogene.

[6] Grando SA (2014). Connections of nicotine to cancer. Nature reviews. Cancer.

[7] Amos CI (2008). Genome-wide association scan of tag SNPs identifies a susceptibility locus for lung cancer at 15q25.1. Nature genetics.

[8] Thorgeirsson TE (2008). A variant associated with nicotine dependence, lung cancer and peripheral arterial disease. Nature.

[9] Hung RJ (2008). A susceptibility locus for lung cancer maps to nicotinic acetylcholine receptor subunit genes on 15q25. Nature.

[10] Wen L, Jiang K, Yuan W, Cui W, Li MD (2016). Contribution of Variants in CHRNA5/A3/B4 Gene Cluster on Chromosome 15 to Tobacco Smoking: From Genetic Association to Mechanism. Molecular neurobiology.

[11] Bell JT (2011). DNA methylation patterns associate with genetic and gene expression variation in HapMap cell lines. Genome Biol.

[12] Laurent L (2010). Dynamic changes in the human methylome during differentiation. Genome research.

[13] Law JA, Jacobsen SE (2010). Establishing, maintaining and modifying DNA methylation patterns in plants and animals. Nature reviews. Genetics.

[14] Jones A (2013). Role of DNA methylation and epigenetic silencing of HAND2 in endometrial cancer development. PLoS medicine.

[15] Teschendorff AE (2012). Epigenetic variability in cells of normal cytology is associated with the risk of future morphological transformation. Genome medicine.

[16] Sundar IK, Mullapudi N, Yao H, Spivack SD, Rahman I (2011). Lung cancer and its association with chronic obstructive pulmonary disease: update on nexus of epigenetics. Curr Opin Pulm Med.

[17] Pastrello C (2014). Integration, visualization and analysis of human interactome. Biochemical and biophysical research communications.

[18] Maccani JZ, Maccani MA (2015). Altered placental DNA methylation patterns associated with maternal smoking: current perspectives. Advances in genomics and genetics.

[19] Gao X, Jia M, Zhang Y, Breitling LP, Brenner H (2015). DNA methylation changes of whole blood cells in response to active smoking exposure in adults: a systematic review of DNA methylation studies. Clinical epigenetics.

[20] Flom JD (2011). Prenatal smoke exposure and genomic DNA methylation in a multiethnic birth cohort. Cancer epidemiology, biomarkers & prevention: a publication of the American Association for Cancer Research, cosponsored by the American Society of Preventive Oncology.

[21] Furniss CS, Marsit CJ, Houseman EA, Eddy K, Kelsey KT (2008). Line region hypomethylation is associated with lifestyle and differs by human papillomavirus status in head and neck squamous cell carcinomas. Cancer epidemiology, biomarkers & prevention: a publication of the American Association for Cancer Research, cosponsored by the American Society of Preventive Oncology.

[22] Yao HP, Zhou YQ, Zhang R, Wang MH (2013). MSP-RON signalling in cancer: pathogenesis and therapeutic potential. Nature reviews. Cancer.

[23] Altucci L, Leibowitz MD, Ogilvie KM, de Lera AR, Gronemeyer H (2007). RAR and RXR modulation in cancer and metabolic disease. Nature reviews. Drug discovery.

[24] Guida F (2015). Dynamics of smoking-induced genome-wide methylation changes with time since smoking cessation. Human molecular genetics.

[25] Dogan MV (2014). The effect of smoking on DNA methylation of peripheral blood mononuclear cells from African American women. BMC genomics.

[26] Zeilinger S (2013). Tobacco smoking leads to extensive genome-wide changes in DNA methylation. PloS one.

[27] Zudaire E (2008). The aryl hydrocarbon receptor repressor is a putative tumor suppressor gene in multiple human cancers. The Journal of clinical investigation.

[28] Shenker NS (2013). Epigenome-wide association study in the European Prospective Investigation into Cancer and Nutrition (EPIC-Turin) identifies novel genetic loci associated with smoking. Human molecular genetics.

[29] Zhang Y (2014). F2RL3 methylation in blood DNA is a strong predictor of mortality. International journal of epidemiology.

[30] Fasanelli F (2015). Hypomethylation of smoking-related genes is associated with future lung cancer in four prospective cohorts. Nature communications.

[31] Joehanes R (2016). Epigenetic Signatures of Cigarette SmokingCLINICAL PERSPECTIVE. Circulation: Cardiovascular Genetics.

[32] Guilhamon P (2013). Meta-analysis of IDH-mutant cancers identifies EBF1 as an interaction partner for TET2. Nature communications.

[33] Novakovic B (2014). Postnatal stability, tissue, and time specific effects of AHRR methylation change in response to maternal smoking in pregnancy. Epigenetics.

[34] Tsay JJ, Tchou-Wong KM, Greenberg AK, Pass H, Rom WN (2013). Aryl hydrocarbon receptor and lung cancer. Anticancer Res.

[35] Szklarczyk D (2015). STRING v10: protein-protein interaction networks, integrated over the tree of life. Nucleic acids research.

[36] Fiuza UM, Arias AM (2007). Cell and molecular biology of Notch. The Journal of endocrinology.

[37] Radtke F, Raj K (2003). The role of Notch in tumorigenesis: oncogene or tumour suppressor?. Nature reviews. Cancer.

[38] Medina PP, Sanchez-Cespedes M (2008). Involvement of the chromatin-remodeling factor BRG1/SMARCA4 in human cancer. Epigenetics: official journal of the DNA Methylation Society.

[39] Kretzmer H (2015). DNA methylome analysis in Burkitt and follicular lymphomas identifies differentially methylated regions linked to somatic mutation and transcriptional control. Nature genetics.

[40] Jelinic P (2014). Recurrent SMARCA4 mutations in small cell carcinoma of the ovary. Nature genetics.

[41] Medina PP (2004). Genetic and epigenetic screening for gene alterations of the chromatin-remodeling factor, SMARCA4/BRG1, in lung tumors. Genes, chromosomes & cancer.

[42] Britson J, Barton F, Balko JM, Black EP (2009). Deregulation of DUSP activity in EGFR-mutant lung cancer cell lines contributes to sustained ERK1/2 signaling. Biochem bioph res co.

[43] Chitale D (2009). An integrated genomic analysis of lung cancer reveals loss of DUSP4 in EGFR-mutant tumors. Oncogene.

[44] Armes JE (2004). Candidate tumor-suppressor genes on chromosome arm 8p in early-onset and high-grade breast cancers. Oncogene.

[45] Waha A (2010). Epigenetic downregulation of mitogen-activated protein kinase phosphatase MKP-2 relieves its growth suppressive activity in glioma cells. Cancer research.

[46] Lawan A (2011). Deletion of the dual specific phosphatase-4 (DUSP-4) gene reveals an essential non-redundant role for MAP kinase phosphatase-2 (MKP-2) in proliferation and cell survival. Journal of Biological Chemistry.

[47] Gröschl B (2013). Expression of the MAP kinase phosphatase DUSP4 is associated with microsatellite instability in colorectal cancer (CRC) and causes increased cell proliferation. International Journal of Cancer.

[48] Kramer A, Green J, Pollard J, Tugendreich S (2014). Causal analysis approaches in Ingenuity Pathway Analysis. Bioinformatics.

[49] Glaab E, Baudot A, Krasnogor N, Schneider R, Valencia A (2012). EnrichNet: network-based gene set enrichment analysis. Bioinformatics.

[50] Backes C (2007). GeneTrail–advanced gene set enrichment analysis. Nucleic acids research.

[51] Agresti A (1992). A survey of exact inference for contingency tables. Statistical science.

[52] Benjamini, Y. & Hochberg, Y. Controlling the false discovery rate: a practical and powerful approach to multiple testing. *Journal of the royal statistical society*. *Series B* (*Methodological*) 289–300 (1995).

[53] Ogata H (1999). KEGG: Kyoto Encyclopedia of Genes and Genomes. Nucleic acids research.

[54] Kelder T (2012). WikiPathways: building research communities on biological pathways. Nucleic acids research.

[55] Nishimura D (2001). BioCarta. Biotech Software & Internet Report: The Computer Software Journal for Scient.

[56] Maere S, Heymans K, Kuiper M (2005). BiNGO: a Cytoscape plugin to assess overrepresentation of gene ontology categories in biological networks. Bioinformatics.

[57] Berkopec A (2007). HyperQuick algorithm for discrete hypergeometric distribution. Journal of Discrete Algorithms.

[58] Supek F, Bošnjak M, Škunca N, Šmuc T (2011). REVIGO summarizes and visualizes long lists of gene ontology terms. PloS one.

[59] Shannon P (2003). Cytoscape: a software environment for integrated models of biomolecular interaction networks. Genome research.

[60] The Cancer Genome Atlas Research Networks, Comprehensive genomic characterization of squamous cell lung cancers. *Nature* **489**, 519–525, doi:10.1038/nature11404 (2012). 10.1038/nature11404 PMC3466113 22960745

[61] The Cancer Genome Atlas Research Networks, Comprehensive molecular profiling of lung adenocarcinoma. *Nature* **511**, 543–550, doi:10.1038/nature13385 (2014). 10.1038/nature13385 PMC4231481 25079552

[62] Tomczak K, Czerwinska P, Wiznerowicz M (2015). The Cancer Genome Atlas (TCGA): an immeasurable source of knowledge. Contemporary oncology.

[63] Koch A, De Meyer T, Jeschke J, Van Criekinge W (2015). MEXPRESS: visualizing expression, DNA methylation and clinical TCGA data. BMC genomics.

[64] Gayen A (1951). The frequency distribution of the product-moment correlation coefficient in random samples of any size drawn from non-normal universes. Biometrika.

[65] Wilcoxon F (1945). Individual comparisons by ranking methods. Biometrics bulletin.

[66] Chen H, Boutros PC (2011). VennDiagram: a package for the generation of highly-customizable Venn and Euler diagrams in R. BMC bioinformatics.

[67] Wickham, H. *ggplot2: elegant graphics for data analysis* (Springer, 2016).

