# Supplementary material for: Establishment of a Strong Link Between Smoking and Cancer Pathogenesis through DNA Methylation Analysis
Source: Sci Rep. 2017 May 12;7:1811. doi: 10.1038/s41598-017-01856-4 (PMC5431893; doi:10.1038/s41598-017-01856-4)
Supplement: Supplementary file 1 — Supplementary PDF Filepdf [file 41598_2017_1856_MOESM1_ESM.pdf]

Establishment of a Strong Link Between Smoking and Cancer Pathogenesis through DNA Methylation Analysis

Yunlong Ma[1,2)] and Ming D. Li[1,2,3)]

1) State Key Laboratory for Diagnosis and Treatment of Infectious Diseases, Collaborative Innovation Center for Diagnosis and Treatment of Infectious Diseases, The First Affiliated Hospital, Zhejiang University School of Medicine, Hangzhou, China; 2) Research Center for Air Pollution and Health, Zhejiang University, Hangzhou; 3) Institute for NeuroImmune Pharmacology, Seton Hall University, South Orange, NJ, United States

Supplementary Figures

Supplemental Figure S1: The data collection pipeline for current study.

Supplemental Figure S2: Functional enrichment analysis of the two-hint-based genes from blood and buccal samples by using IPA software. Top 5 terms of different categories ranked by p-values are shown. The dark blue box present the name of functional category. The light blue box present the specific category and the number in parenthesis represent the number of genes enriched in the specific category.

Supplemental Figure S3: Distributions of methylation loci significantly correlated with RNA expression. (a) positive correlation in LUAD; (b) negative correlation in LUAD; (c) positive correlation in LUSC; (d) negative correlation in LUSC.

Supplemental Figure S4: Correlation of methylation loci with RNA expression in LUAD samples. (a) negative correlation of SMAD6_cg27514433 with RNA expression of SMAD6 gene; (b) negative correlation of NFE2L2_cg26271591 with RNA expression of NFE2L2 gene; (c) positive correlation of AKT3_cg11314684 with RNA expression of AKT3 gene; (d) positive correlation of AHRR_cg02385153 with RNA expression of AHRR gene.

Supplemental Figure S5: Correlation of methylation loci with RNA expression in LUSC samples. (a) negative correlation of SMAD6_cg27514433 with RNA expression of SMAD6 gene; (b) negative correlation of NFE2L2_cg26271591 with RNA expression of NFE2L2 gene; (c) positive correlation of AKT3_cg11314684 with RNA expression of AKT3 gene; (d) positive correlation of AHRR_cg02385153 with RNA expression of AHRR gene.

Supplemental Figure S6: Two methylation-probes of *DUSP4* in LUSC samples. (a) Correlation of cg07151117-probe with RNA expression of *DUSP4* gene in control and cancer of LUSC samples. (b) Correlation of cg24379915-probe with RNA expression of *DUSP4* gene in control and cancer of LUSC samples. (c) Methylation of cg07151117-probe in control and cancer of LUSC samples. (d) Methylation of cg24379915-probe in control and cancer of LUSC samples. P-value is from the Wilcoxon-rank sum test.

Supplemental Figure S7: Methylation loci correlated with RNA expression of specific gene showed differences between control and LUAD. (a) LRP5_cg04265051; (b) TNF_cg09637172; (c) CDK6_cg06688763; (d) NFE2L2_cg26271591; (e) AKT3_cg11314684; (f) AHRR_cg02385153; (g) RARA_cg19572487; (h) AHRR_cg11902777; (i) PIP4K2A_cg04813697. P-value is from the Wilcoxon-rank sum test.

Supplemental Figure S8: Methylation loci correlated with RNA expression of specific gene showed differences between control and LUSC. (a) NOTCH1_cg14120703; (b) TNF_cg09637172; (c) SMARCA4_cg23963476; (d) SMAD6_cg27514333; (e) AKT3_cg11314684; (f) AHRR_cg02385153; (g) RARA_cg19572487; (h) AHRR_cg11902777; (i) PIK3CD_cg07805542. P-value is from the Wilcoxon-rank sum test.

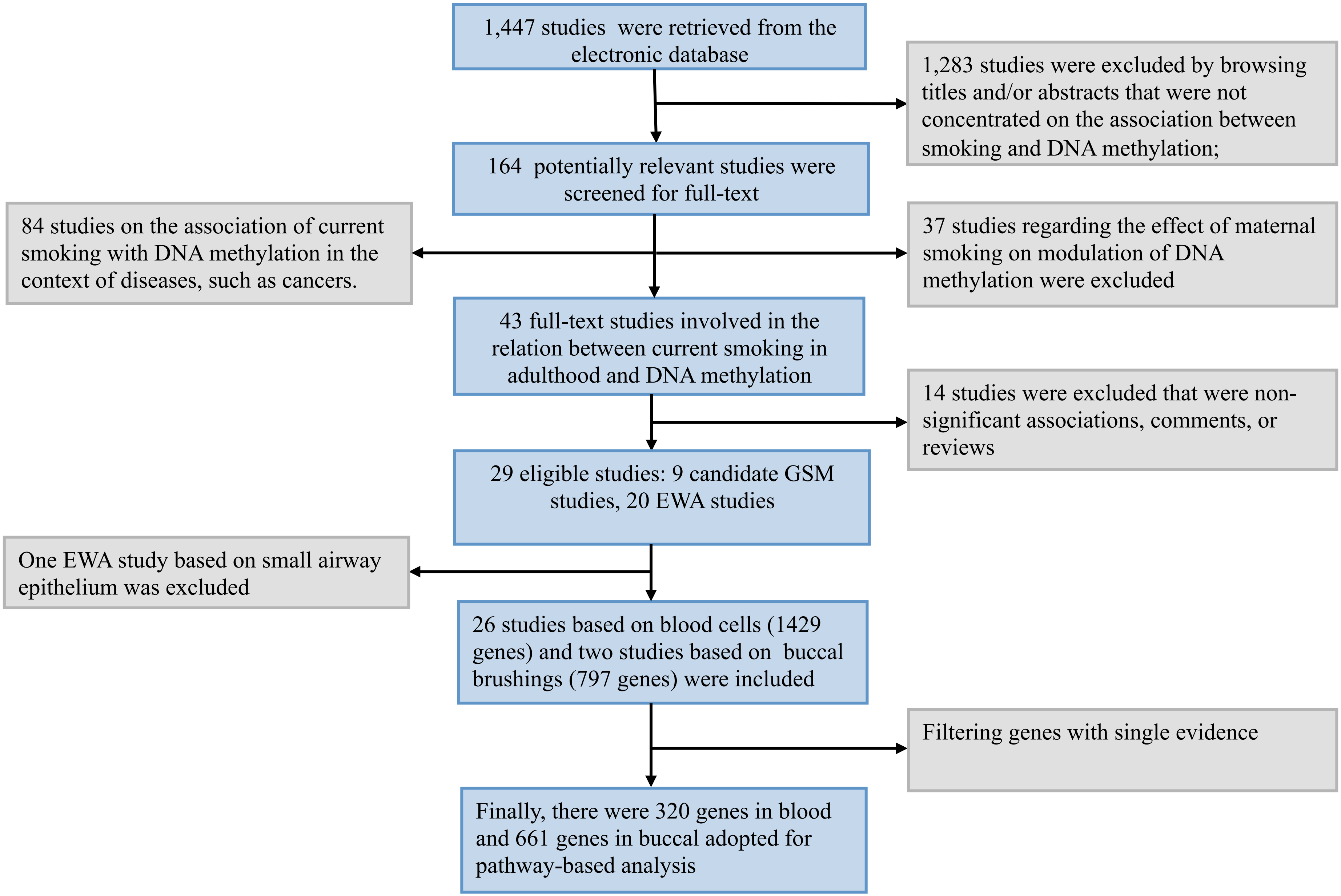

Supplemental Figure S1

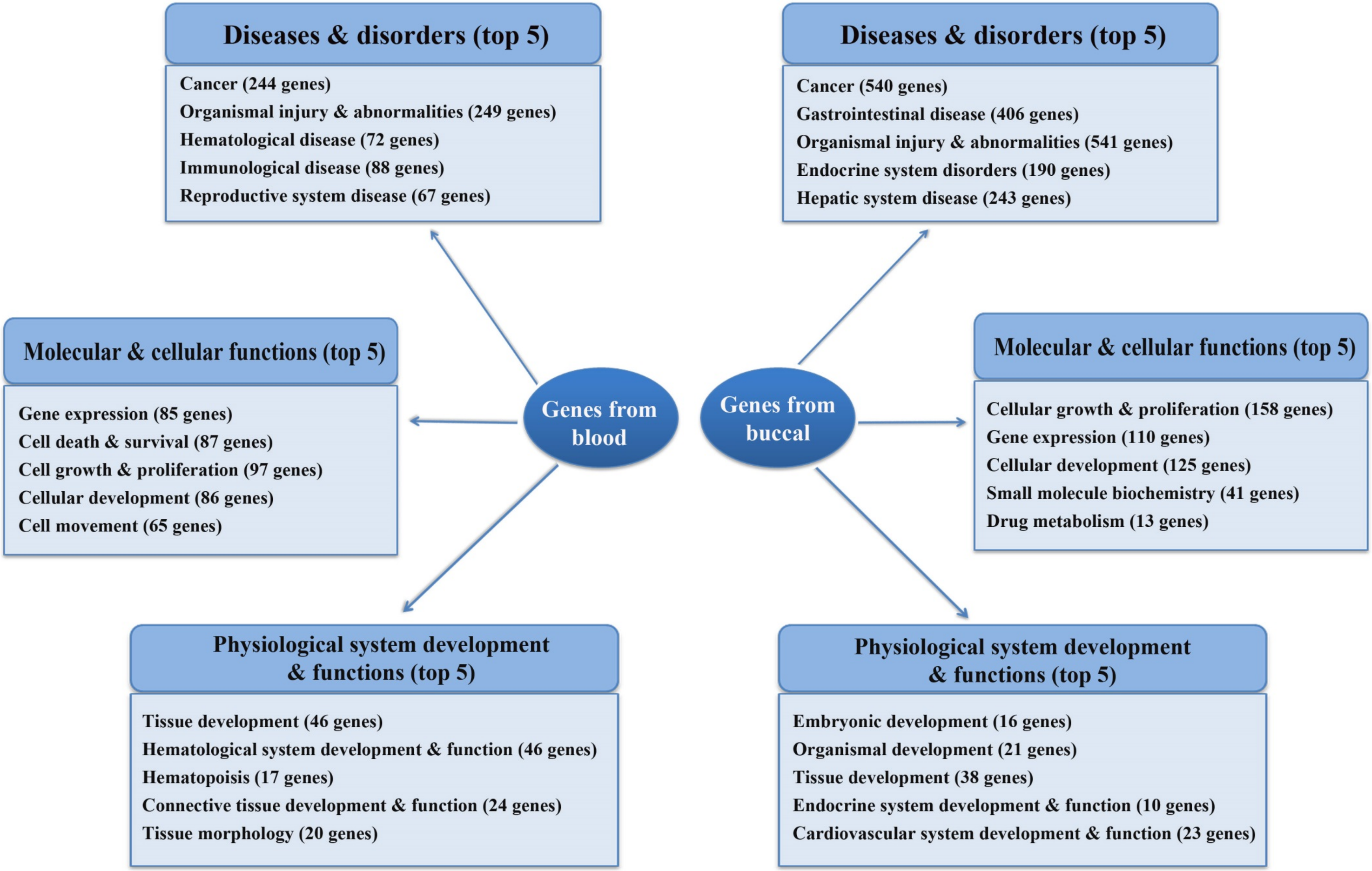

Supplemental Figure S2

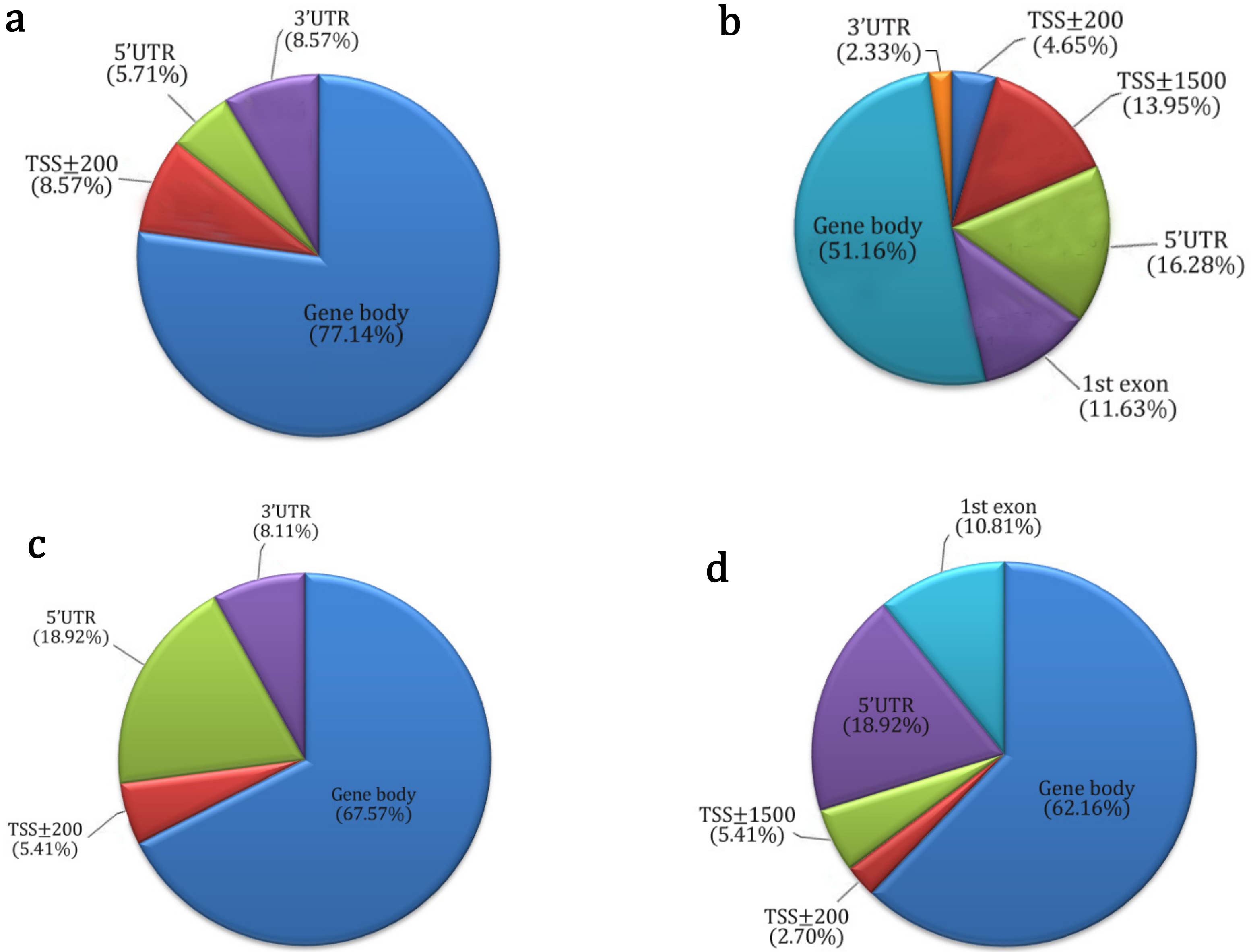

Supplemental Figure S3

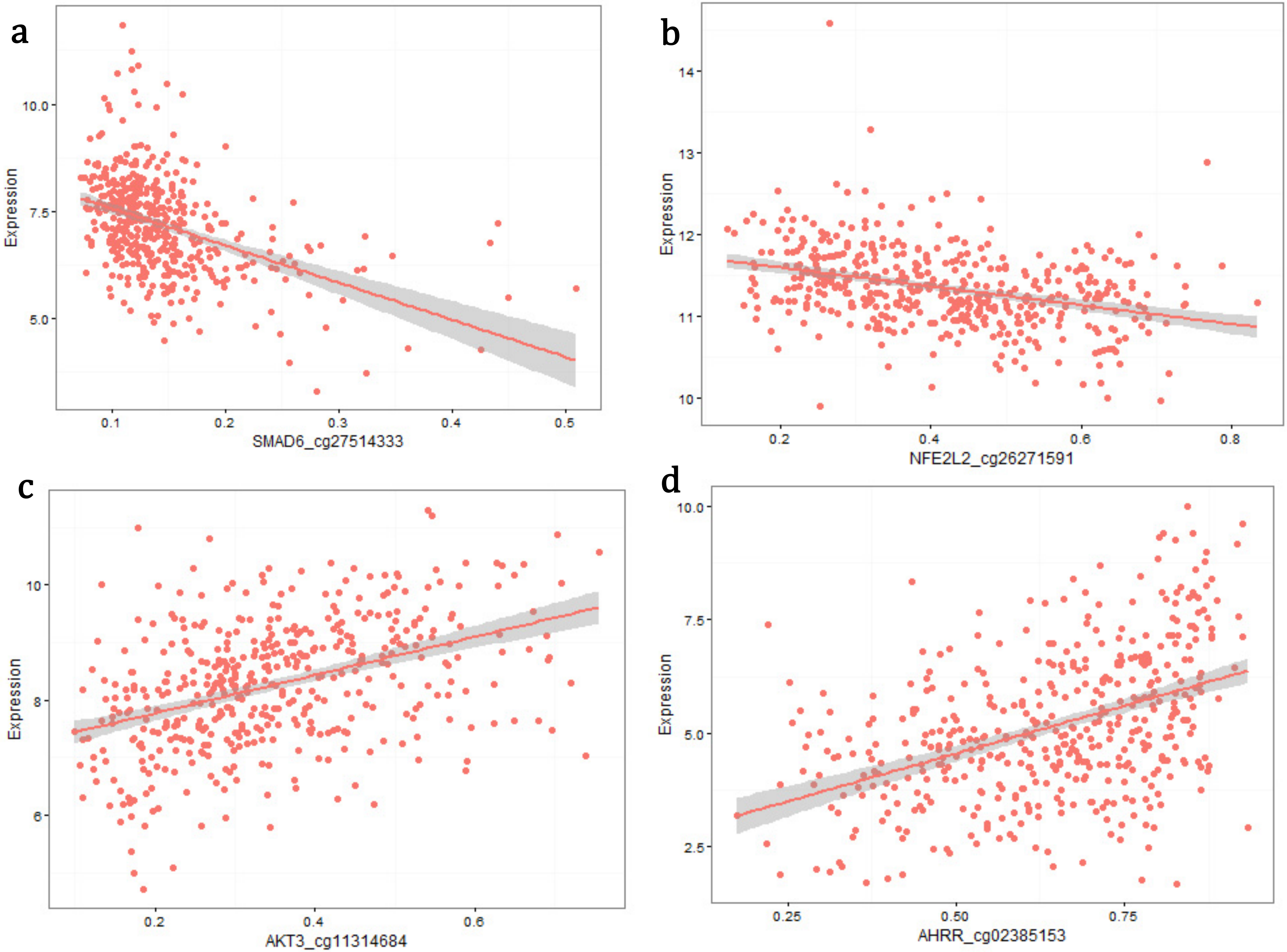

Supplemental Figure S4

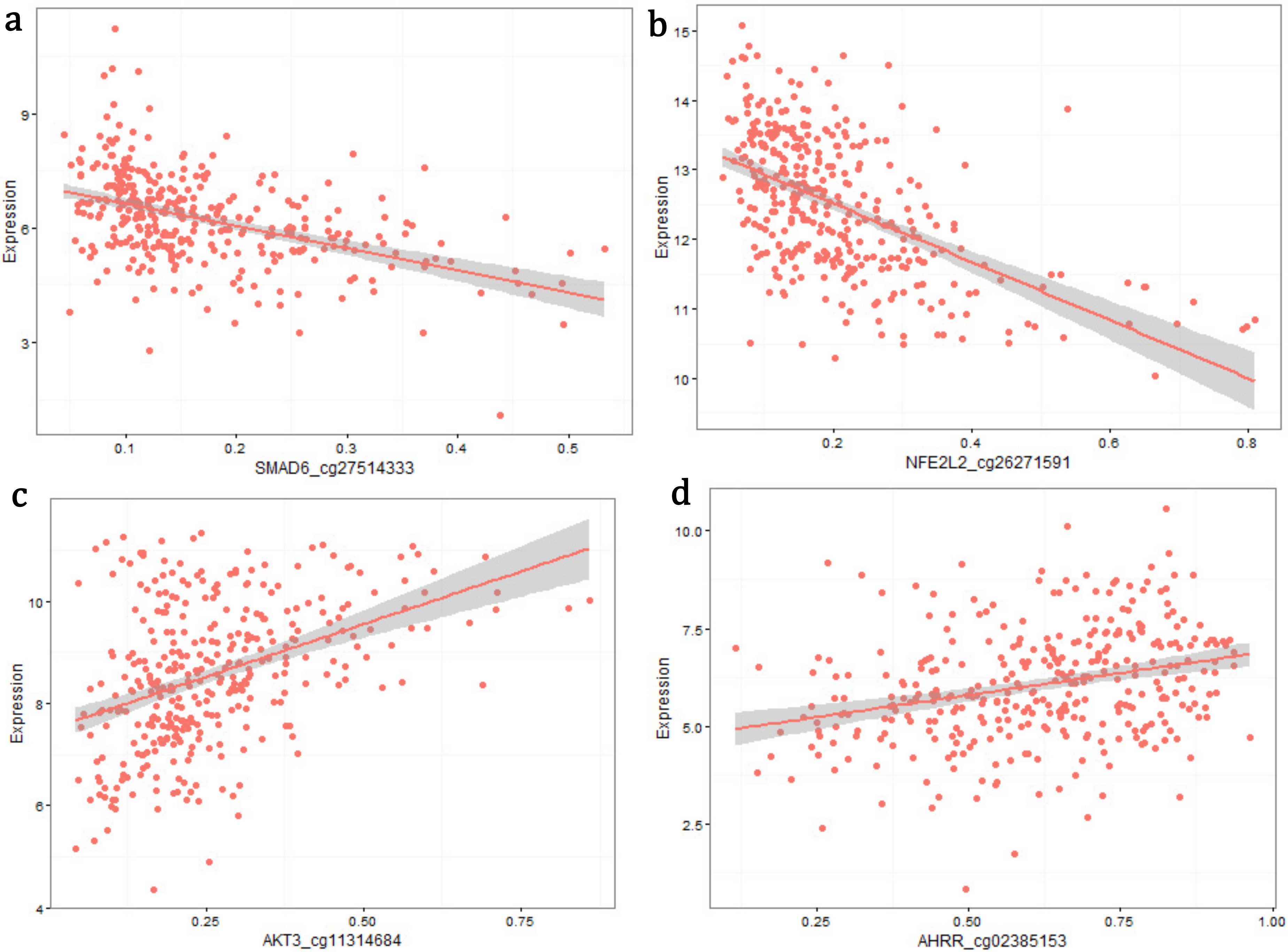

Supplemental Figure S5

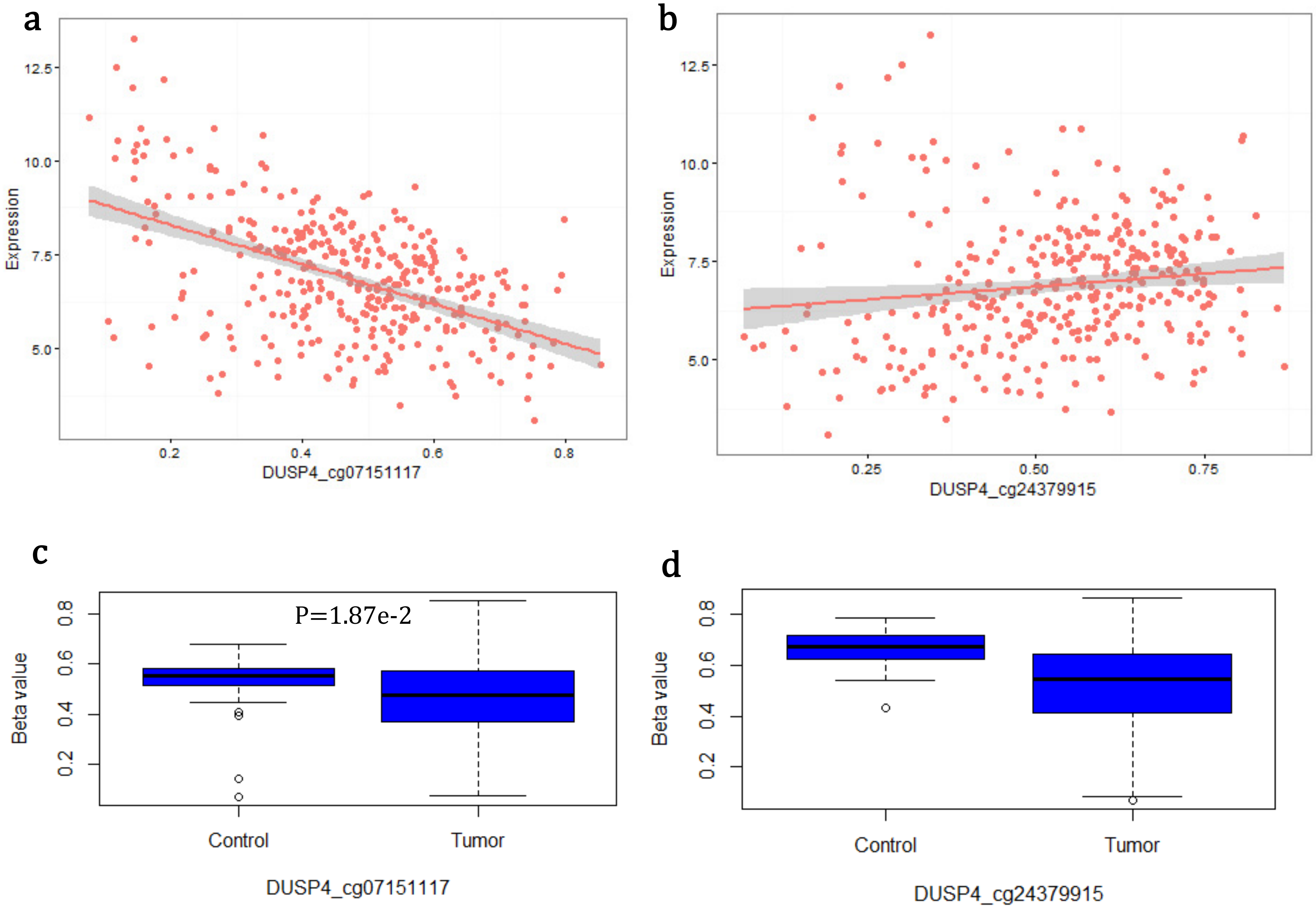

Supplemental Figure S6

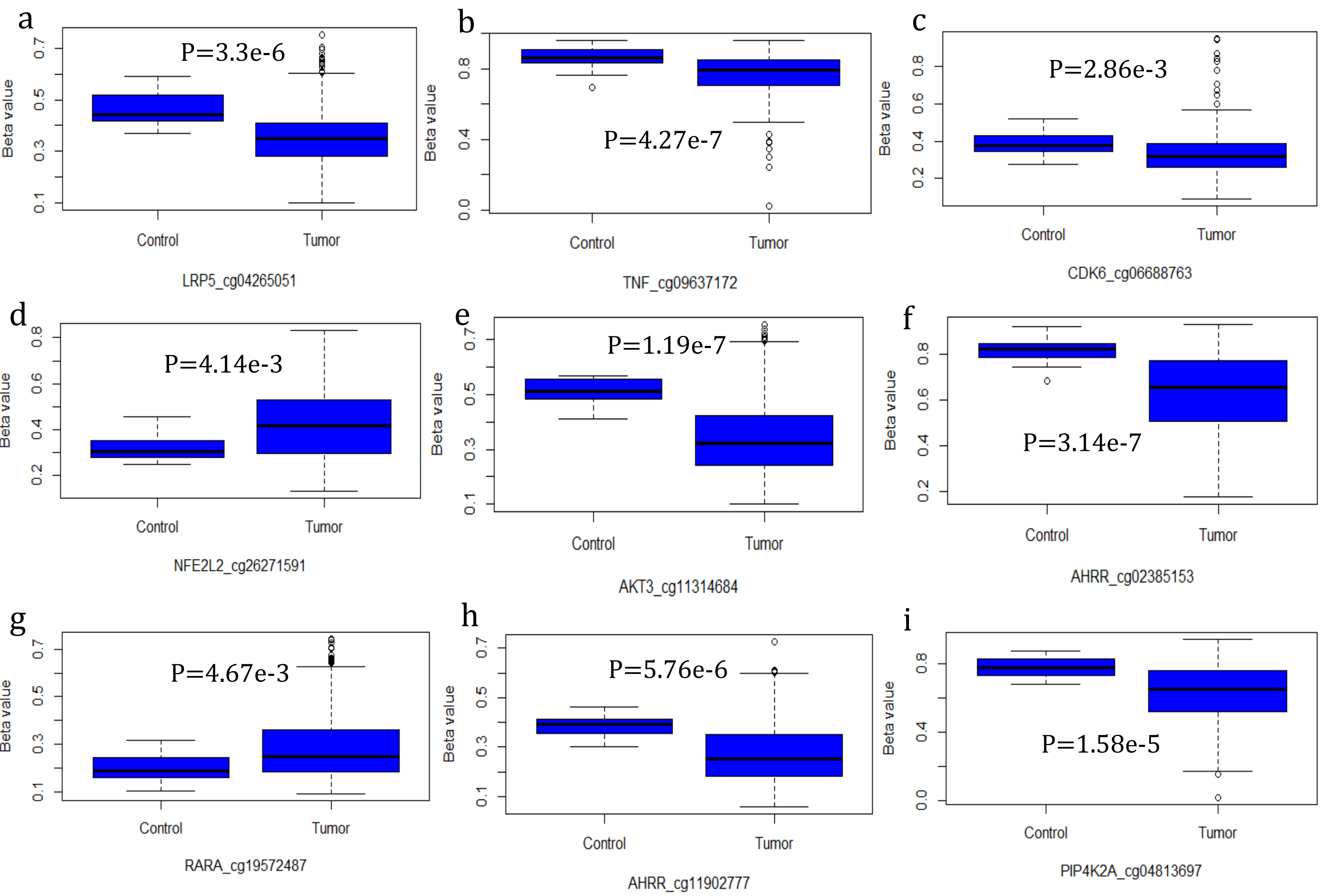

Supplemental Figure S7

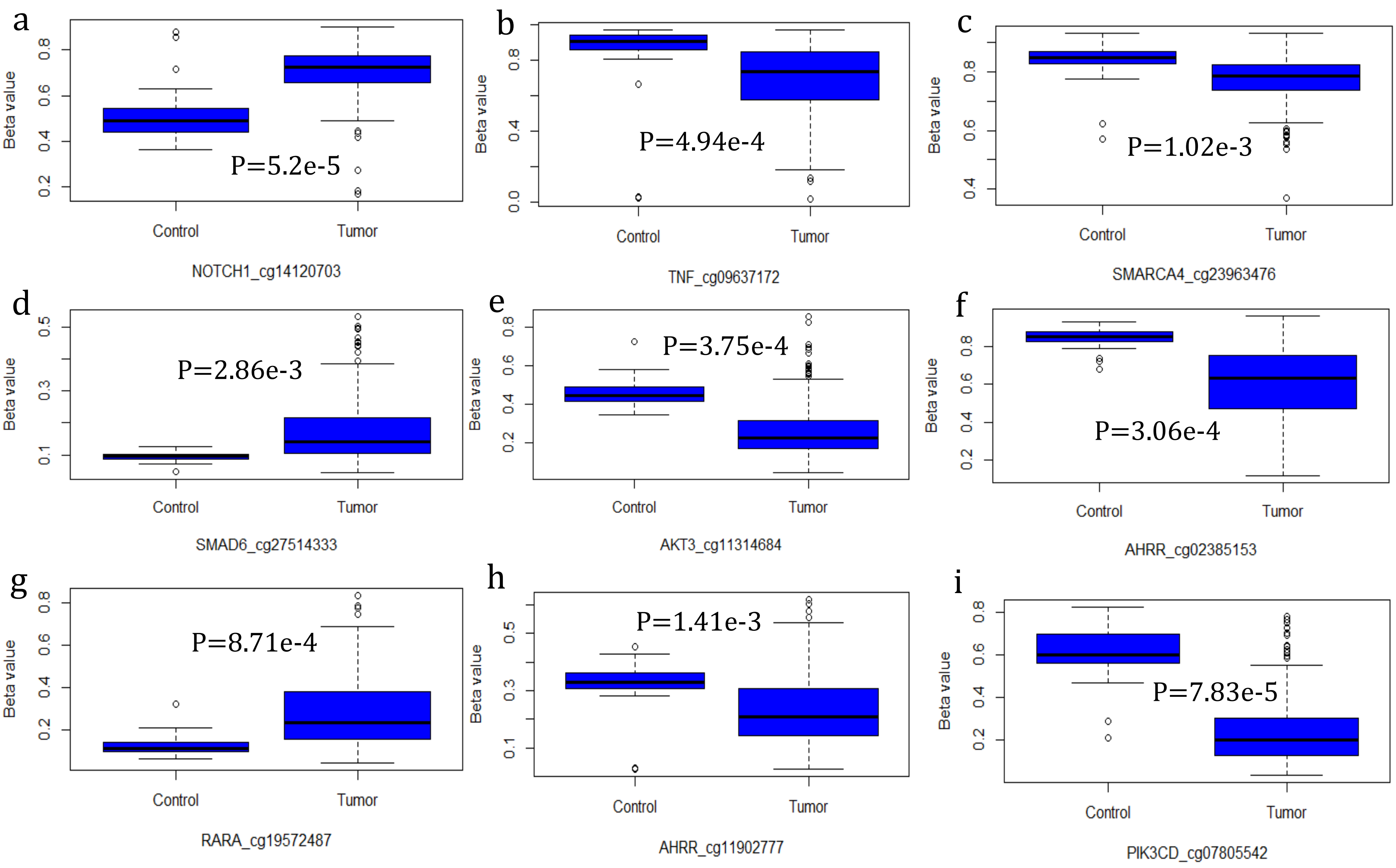

Supplemental Figure S8

Supplemental Table S1 Statistics of the chosen studies based on different biomaterials

Study Method/Platform	#Study	#Total samples	Biomaterial	#Enriched Genes	References
**Candidate gene-specific methylation studies**					
Based on bisulfite conversion	8	5,712	Blood cells	5	[1-8]
Based on the Illumina GoldenGate Methylation Cancer Panel I	1	316	Blood cells	16	[9]
**Epigenome-wide association studies**					
Based on the Illumina HumanMethylation 27K BeadChip	3	3,158	Blood cells	97	[10-12]
Based on the Illumina HumanMethylation 450K BeadChip	14	8,489	Blood cells	1,340	[13-26]
Based on the Illumina HumanMethylation 450K BeadChip	2	1,002	Buccal brushings	797	[27, 28]
Based on the 720K Roche-NimbleGen custom array	1	39	Small airway epithelium	4	[29]

Note: There are a number of 29 epigenetic association studies (N = 18,716 subjects) based on different biomaterials. After filtering duplicated genes, a total of 1,429 included genes supported by at least one item of evidence were initially extracted from 26 studies based on blood samples (N = 17,675 subjects) and a total of 797 genes supported by at least single evidence were selected from 2 studies based on buccal samples (N = 1,002 subjects). Since only one study based on the biomaterial of small airway epithelium, we excluded this study for further analysis.

Supplemental Table S2 Genes enriched by SA-DNAm loci with at least two independent pieces of evidence from blood samples

Gene Symbol	Gene Name	References[a]
ABCC4	ATP-binding cassette, sub-family C (CFTR/MRP), member 4	[16, 22]
ABTB1	ankyrin repeat and BTB (POZ) domain containing 1	[12]
ACACA	acetyl-coenzyme A carboxylase alpha	[13, 22]
ACTB	actin, beta	[13]
ADARB1	adenosine deaminase, RNA-specific, B1 (RED1 homolog rat)	[13, 16]
ADCY9	adenylate cyclase 9	[16, 22]
ADO	2-aminoethanethiol (cysteamine) dioxygenase	[16, 22]
ADRA2A	adrenergic, alpha-2A-, receptor	[16, 22]
ADSL	adenylosuccinate lyase	[13]
AHDC1	AT hook, DNA binding motif, containing 1	[13]
AHRR	aryl hydrocarbon receptor repressor	[13-19, 21-24, 30]
AKT3	v-akt murine thymoma viral oncogene homolog 3 (protein kinase B, gamma)	[12, 17, 22]
ALAS1	aminolevulinate, delta-, synthase 1	[13, 22]
ALPI	alkaline phosphatase, intestinal	[19]
ALPP	alkaline phosphatase, placental (Regan isozyme)	[13, 16, 18, 22]
ALPPL2	alkaline phosphatase, placental-like 2	[13, 18, 19]
ANKFY1	ankyrin repeat and FYVE domain containing 1	[13]
ANKRD11	ankyrin repeat domain 11; hypothetical protein LOC100128265	[13]
ANKRD53	ankyrin repeat domain 53	[16]
ANPEP	alanyl (membrane) aminopeptidase (aminopeptidase N, aminopeptidase M, microsomal aminopeptidase, CD13, p150)	[13, 19, 22]
AP2A2	adaptor-related protein complex 2, alpha 2 subunit	[13, 16]
APBA2	amyloid beta (A4) precursor protein-binding, family A, member 2 (X11-like)	[11]
APLP	Amyloid beta (A4) precursor-like protein 1	[19]
ARHGAP25	Rho GTPase activating protein 25	[11, 12, 19]
ARHGEF3	Rho guanine nucleotide exchange factor (GEF) 3	[13, 22]
ARID5B	AT rich interactive domain 5B	[13, 16, 22]
ARRB1	arrestin, beta 1	[13, 22]
ASB2	ankyrin repeat and SOCS box-containing 2	[13, 16]
ASCC1	activating signal cointegrator 1 complex subunit 1	[13, 16]
ATF4	activating transcription factor 4 (tax-responsive enhancer element B67)	[13, 22]
ATP11A	ATPase, class VI, type 11A	[13, 16]
ATP8A2	ATPase, aminophospholipid transporter-like, class I, type 8A, member 2	[13, 16]
ATP9A	ATPase, class II, type 9A	[13, 22]
AVP	arginine vasopressin (neurophysin II, antidiuretic hormone, diabetes insipidus, neurohypophyseal)	[13]
AVPR1B	arginine vasopressin receptor 1B	[13, 15, 18, 19, 22]
B3GALT4	UDP-Gal:betaGlcNAc beta 1,3-galactosyltransferase, polypeptide 4	[13, 22]
B3GNTL1	UDP-GlcNAc:betaGal beta-1,3-N-acetylglucosaminyltransferase-like 1	[13, 16]

BAIAP2	BAI1-associated protein 2	[13, 16]
BAT1	HLA-B associated transcript 1	[16]
BCAS3	breast carcinoma amplified sequence 3	[13, 16]
BCAS4	breast carcinoma amplified sequence 4	[13, 16]
BCL11B	B-cell CLL/lymphoma 11B (zinc finger protein)	[13]
BRCA2	breast cancer 2	[13, 21]
BTBD11	BTB (POZ) domain containing 11	[13, 22]
C14orf43	chromosome 14 open reading frame 43	[13, 15, 16, 18, 19, 22]
C1orf230	chromosome 1 open reading frame 230	[13]
C1orf93	MGC82733 protein	[16]
C5orf62	MSTP150	[13, 19, 22]
C6orf48	chromosome 6 open reading frame 48; small nucleolar RNA, C/D box 52	[13, 16, 22]
C7orf40	chromosome 7 open reading frame 40	[13, 16, 22]
CACNA1D	calcium channel, voltage-dependent, L type, alpha 1D subunit	[13]
CAPZB	capping protein (actin filament) muscle Z-line, beta	[13, 22]
CARS2	cysteinyl-Trna synthetase 2, mitochondrial (putative)	[13, 22]
CBFB	core-binding factor, beta subunit	[12, 13, 16, 17, 22]
CCDC88C	coiled-coil domain containing 88C	[13, 22]
CD38	CD38 molecule	[13]
CD59	CD59 molecule, complement regulatory protein	[13]
CDK6	cyclin-dependent kinase 6	[13]
CDKN1A	cyclin-dependent kinase inhibitor 1A (p21, Cip1)	[13, 22, 25]
CHD3	Chromodomain-helicase-DNA-binding protein 3	[13]
CHRND	Cholinergic receptor, nicotinic, delta	[18]
CLCN6	chloride channel 6	[12, 22]
CLDND1	claudin domain containing 1	[16, 19]
CNR2	cannabinoid receptor 2 (macrophage)	[13]
CNTNAP2	contactin associated protein-like 2; hypothetical protein LOC750089	[11, 13, 14, 17, 19, 22]
COMMD7	COMM domain containing 7	[13, 22]
COMT	catechol-O-methyltransferase	[16, 31]
CPAMD8	C3 and PZP-like, alpha-2-macroglobulin domain containing 8	[13, 22]
CPOX	coproporphyrinogen oxidase	[13, 16-19, 22]
CRTC1	CREB regulated transcription coactivator 1	[13, 22]
CRTC2	CREB regulated transcription coactivator 2	[16, 22]
CSNK1G3	casein kinase 1, gamma 3; similar to casein kinase 1, gamma 3	[16]
CSRNP1	cysteine-serine-rich nuclear protein 1	[13, 22]
CUTA	XCC0520	[13, 22]
CUX1	similar to CCAAT displacement protein isoform b; cut-like homeobox 1	[13, 16]
CXCR5	chemokine (C-X-C motif) receptor 5	[13, 16, 22]
CXXC5	CXXC finger protein 5	[13, 16]
CYP1A1	cytochrome P450, family 1, subfamily a, polypeptide 1	[13, 18]

DDIT4	DNA-damage-inducible transcript 4	[13, 22]
DGKA	PMI2750	[13]
DKFZp434J0226	hypothetical LOC93429	[13, 18]
DUSP4	dual specificity phosphatase 4	[16, 22]
ECEL1P2	endothelin converting enzyme-like 1, pseudogene 2	[13, 16, 22]
EDC3	enhancer of mRNA decapping 3 homolog (S. cerevisiae)	[13, 22]
EDN2	endothelin 2	[13, 22]
EGR1	early growth response 1	[13, 22]
EIF2C2	eukaryotic translation initiation factor 2C, 1	[13, 22]
EIF4G1	eukaryotic translation initiation factor 4 gamma, 1; similar to eukaryotic translation initiation factor 4, gamma 1 isoform a; similar to Eif4g1 protein	[13, 22]
EPB41	cytoskeletal protein 4.1	[13, 16]
EPB49	erythrocyte membrane protein band 4.9 (dematin)	[22]
ETS1	C-ets-1	[13]
ETS2	E26 avian leukemia oncogene 2, 3' domain	[13, 22]
ETV6	ets variant 6	[13, 19, 22]
F2RL3	coagulation factor II (thrombin) receptor-like 3	[10-13, 15-19, 21, 22, 30, 32]
FAM102A	family with sequence similarity 102, member A	[13]
FAM171A2	hypothetical protein LOC100158440	[13]
FBXL12	F-box and leucine-rich repeat protein 12	[16]
FES	SFV_0530; S0503; SF0497	[25]
FGR	Gardner-Rasheed feline sarcoma viral (Fgr) oncogene homolog	[13]
FGF23	fibroblast growth factor 23	[13, 15]
FMNL1	formin-like 1	[13, 16]
GALNT7	UDP-N-acetyl-alpha-D-galactosamine:polypeptide N-acetylgalactosaminyltransferase 7 (GalNAc-T7)	[13, 22]
GAS5	growth arrest specific 5	[13, 22]
GATA3	Bpet0846	[13]
GFI1	growth factor independent 1 transcription repressor	[13, 15, 16, 19, 22]
GNA12	guanine nucleotide binding protein (G protein) alpha 12	[13, 22]
GNG12	guanine nucleotide binding protein (G protein), gamma 12	[12, 13, 15, 17, 18, 22]
GNG12-AS1	guanine nucleotide binding protein (G protein), gamma 12, antisense RNA 1	[18]
GP5	glycoprotein V (platelet)	[13]
GPR114	G protein-coupled receptor 114	[13]
GPR15	G protein-coupled receptor 15	[11-13, 15-18, 22]
GPR25	G protein-coupled receptor 25	[12]
GPR44	G protein-coupled receptor 44	[13, 22]
GPR55	G protein-coupled receptor 55	[13, 16, 18, 22]
GPR68	G protein-coupled receptor 68	[13, 22]
GPSM3	G-protein signaling modulator 3 (AGS3-like, C. elegans)	[13]

GPX1	glutathione peroxidase 1	[13, 22]
HAP1	huntingtin-associated protein 1	[13]
HIC1	hypermethylated in cancer 1	[13, 33]
HIST1H2BK	histone cluster 1, H2bk	[16]
HIVEP3	human immunodeficiency virus type I enhancer binding protein 3	[13, 19]
HMGA1	similar to high mobility group protein isoform I; high mobility group AT-hook 1	[13]
HNRNPA1	heterogeneous nuclear ribonucleoprotein A1	[13, 16, 22]
HNRNPA2B1	heterogeneous nuclear ribonucleoprotein A2/B1	[13, 22]
HNRNPF	heterogeneous nuclear ribonucleoprotein F	[13, 22]
HNRPUL1	Heterogeneous nuclear ribonucleoprotein U-like 1	[12]
HOXA7	homeobox A7	[19, 22]
HPN	hepsin	[13]
HRH1	histamine receptor H1	[16, 22]
HTT	huntingtin	[13, 15, 22]
HUS1	HUS1 checkpoint homolog (S. pombe)	[19, 22]
IER3	immediate early response 3	[18]
IL3	interleukin 3 (colony-stimulating factor, multiple)	[13]
IMMP2L	IMP2 inner mitochondrial membrane peptidase-like (S. cerevisiae)	[13, 19]
INF2	hypothetical protein MGC81508	[13]
INTS1	integrator complex subunit 1	[13, 22]
ISL1	ISL LIM homeobox 1	[9]
ITGAL	integrin, alpha L (antigen CD11A (p180), lymphocyte function-associated antigen 1; alpha polypeptide)	[13, 16, 18, 22]
ITGAM	integrin, alpha M (complement component 3 receptor 3 subunit)	[13]
ITPK1	Inositol-tetrakisphosphate 1-kinase	[13, 19, 22]
JMJD8	jumonji domain containing 8	[13, 22]
JTB	jumping translocation breakpoint	[22]
KAZALD1	MGC80370 protein	[13, 16, 22]
KCNN4	potassium intermediate/small conductance calcium-activated channel, subfamily N, member 4	[22]
KCNQ1	potassium voltage-gated channel, KQT-like subfamily, member 1	[13, 15, 18, 19]
KCNQ1OT1	KCNQ1 overlapping transcript 1	[13, 15, 18, 19, 22]
KDM2B	lysine (K)-specific demethylase 2B	[13, 16, 22]
KIAA0087	KIAA0087	[13, 18, 22]
KIAA1949	KIAA1949	[13, 22]
KRT8	predicted gene 5604; keratin 8	[13]
KSR1	kinase suppressor of ras	[16]
LDLR	low density lipoprotein receptor	[13, 16, 22]
LIM2	lens intrinsic membrane protein 2, 19kDa	[11, 12]
LINC00299	Long intergenic non-protein coding RNA 299	[18]
LINGO3	leucine rich repeat and Ig domain containing 3	[13]
LMF1	lipase maturation factor 1	[13, 16]
LOC10013093	hypothetical LOC100130933	[13, 22]

3		
LOC390594	kelch repeat and BTB (POZ) domain containing 13	[13]
LRP5	low density lipoprotein receptor-related protein 5	[13, 16, 18, 19, 22]
LRRC32	leucine rich repeat containing 32	[12, 13, 22]
LRRN3	leucine rich repeat neuronal 3	[11, 13, 16, 17, 19, 22]
LTB	lymphotoxin B	[13]
MACROD1	RIKEN cDNA D930010J01 gene	[16]
MAD1L1	MAD1 mitotic arrest deficient-like 1 (yeast)	[13, 22]
MAML2	mastermind-like 2 (Drosophila)	[13, 16]
MAN1C1	similar to Mannosyl-oligosaccharide 1,2-alpha-mannosidase IC (Processing alpha-1,2-mannosidase IC) (Alpha-1,2-mannosidase IC) (Mannosidase alpha class 1C member 1) (HMIC); mannosidase, alpha, class 1C, member 1	[13]
MAOA	monoamine oxidase A	[34, 35]
MAS1L	MAS1 oncogene-like	[13]
MBP	myelin basic protein	[13, 22]
MCF2L	similar to MCF.2 cell line derived transforming sequence-like; MCF.2 cell line derived transforming sequence-like	[13]
MFHAS1	malignant fibrous histiocytoma amplified sequence 1	[13]
MIR1204	microRNA 1204	[13]
MIR548H4	microRNA 548h-4	[13, 16]
MMP9	matrix metallopeptidase 9 (gelatinase B, 92kDa gelatinase, 92kDa type IV collagenase)	[13]
MOBKL2A	MOB1, Mps One Binder kinase activator-like 2A (yeast)	[13, 22]
MORG1	Mitogen-activated protein kinase organizer 1	[13, 22]
MTSS1	metastasis suppressor 1	[13, 22]
MYH10	myosin, heavy chain 10, non-muscle; myosin, heavy chain 11, smooth muscle; similar to Myosin-11 (Myosin heavy chain, gizzard smooth muscle); similar to myosin, heavy chain 9, non-muscle	[13, 16]
MYO10	myosin 10	[13, 16]
MYO1G	myosin IG	[13, 15, 18, 19, 21, 22]
NAV2	similar to neuron navigator 2	[13, 22]
NCAPH	13S condensin XCAP-H subunit	[13, 22]
NCBP1	nuclear cap binding protein subunit 1	[12]
NCF4	neutrophil cytosolic factor 4,	[12, 13, 19, 22]
NCOR2	nuclear receptor co-repressor 2	[13, 16]
NCRNA00114	non-protein coding RNA 114	[13, 22]
NFATC1	nuclear factor of activated T-cells, cytoplasmic, calcineurin-dependent 1	[13, 16]
NFE2	nuclear factor (erythroid-derived 2)	[13, 22]
NFE2L2	nuclear factor (erythroid-derived 2)-like 2	[13, 19, 22]
NFE2L3	nuclear factor (erythroid-derived 2)-like 3	[13, 22]
NME3	non-metastatic cells 3, protein expressed in	[12, 13]

NOS1AP	nitric oxide synthase 1 (neuronal) adaptor protein	[13, 19, 22]
NOTCH1	Notch homolog 1, translocation-associated (Drosophila)	[16, 22]
NR2F6	nuclear receptor subfamily 2, group F, member 6; hypothetical LOC468317	[9, 22]
OR2B6	olfactory receptor, family 2, subfamily B, member 6	[12, 22]
PAK4	p21 protein (Cdc42/Rac)-activated kinase 4	[13, 22]
PARD3	par-3 partitioning defective 3 homolog (C. elegans)	[13, 22]
PBX2	pre-B-cell leukemia transcription factor 2	[13, 16]
PCDH9	protocadherin 9	[13, 16]
PDZD2	PDZ domani containing 2	[13, 15]
PEX10	peroxisome biogenesis factor 10	[22]
PHF19	PHD finger protein 19	[13]
PIK3CD	phosphoinositide-3-kinase, catalytic, delta polypeptide	[13, 16, 22]
PIK3R5	phosphoinositide-3-kinase, regulatory subunit 5	[13, 22]
PIP4K2A	phosphatidylinositol-5-phosphate 4-kinase, type II, alpha	[13, 22]
PIP5K1C	phosphatidylinositol-4-phosphate 5-kinase, type I, gamma	[13, 16]
PITPNA	phosphatidylinositol transfer protein, alpha	[13]
PITPNM1	phosphatidylinositol transfer protein, membrane-associated 1	[13, 22]
PLEK	pleckstrin	[13]
PMAIP1	phorbol-12-myristate-13-acetate-induced protein 1	[12, 22]
PMEPA1	prostate transmembrane protein, androgen induced 1	[13, 16]
PNPLA7	patatin-like phospholipase domain containing 7	[16]
POLK	polymerase (DNA directed) kappa	[13, 22]
POU3F1	POU domain gene 50	[12]
PPP1R15A	protein phosphatase 1, regulatory (inhibitor) subunit 15A	[19, 22]
PRDM16	PR domain containing 16	[13, 16]
PRKAR1B	protein kinase, cAMP dependent regulatory, type I beta	[13, 22]
PRKCZ	protein kinase C, zeta	[16]
PRSS23	protease, serine, 23	[13, 15, 18, 19, 22]
PSEN2	presenilin-beta	[18]
PSMB8	proteasome (prosome, macropain) subunit, beta type, 8 (large multifunctional peptidase 7)	[13, 16]
PTK2	PTK2 protein tyrosine kinase 2	[13, 16, 18, 22]
PTK6	PTK6 protein tyrosine kinase 6	[13]
PTPN6	protein tyrosine phosphatase, non-receptor type 6	[13, 17]
PTPRN2	protein tyrosine phosphatase, receptor type, N polypeptide 2	[16, 19]
PVT1	Pvt1 oncogene (non-protein coding)	[13]
RAB32	RAB32, member RAS oncogene family	[9]
RARA	SGR_1063	[13, 16, 18, 19, 22]
RARG	retinoic acid receptor, gamma	[13, 16, 22]
RASA3	RAS p21 protein activator 3	[13]
RASSF5	inhibitor of kappa light polypeptide gene enhancer in B-cells, kinase epsilon; similar to Ras association (RalGDS/AF-6) domain family 5 isoform D	[13, 16]
RBM47	RNA binding motif protein 47	[13]

RECQL5	RecQ protein-like 5	[13, 22]
REM2	RAS (RAD and GEM)-like GTP binding 2	[13]
RGS12	regulator of G-protein signaling 12	[13, 16]
RHBDL3	rhomboid, veinlet-like 3 (Drosophila)	[22]
RIN3	Ras and Rab interactor 3	[13]
RNASEK	ribonuclease, RNase K	[13, 22]
RNF44	ring finger protein 44	[13, 22]
RPH3A	rabphilin 3A homolog	[22, 25]
RPL3	ribosomal protein L3	[13, 22]
RPL35	50S ribosomal protein L35	[22]
RPS18	30S ribosomal protein S18, chloroplastic	[13, 16]
RPS6KA2	ribosomal protein S6 kinase, 90kDa, polypeptide 2	[13, 18]
RPTOR	regulatory associated protein of MTOR, complex 1	[16]
RRS1	predicted gene 5067; RRS1 ribosome biogenesis regulator homolog (S. cerevisiae)	[13, 22]
RUNX3	runt-related transcription factor 3	[13]
RXRB	retinoid X receptor, beta	[13]
SDF4	stromal cell derived factor 4	[13]
SDHA	CFPG_454	[16, 19, 22]
SEMA7A	semaphorin 7A, GPI membrane anchor (John Milton Hagen blood group)	[12, 13, 19, 22]
SEPT9	septin 9	[13]
SERINC5	serine incorporator 5	[13, 22]
SFRS13A	FUS interacting protein (serine/arginine-rich) 1	[13, 22]
SIN3B	transcriptional regulator, SIN3B (yeast)	[13, 16, 22]
SKI	v-ski sarcoma viral oncogene homolog	[13, 16, 22, 25]
SLA2	Src-like-adaptor 2	[13, 22]
SLC1A5	solute carrier family 1 (neutral amino acid transporter), member 5	[13, 18]
SLC20A1	solute carrier family 20 (phosphate transporter), member 1	[22]
SLC22A15	solute carrier family 22, member 15	[13, 22]
SLC40A1	solute carrier family 40 (iron-regulated transporter), member 1	[13, 16]
SLC43A2	solute carrier family 43, member 2	[13]
SLFN13	schlafen family member 13	[13]
SMAD6	SMAD family member 6	[13, 22]
SMARCA4	SWI/SNF related, matrix associated, actin dependent regulator of chromatin, subfamily a, member 4	[16]
SMG6	Smg-6 homolog, nonsense mediated mRNA decay factor (C. elegans)	[22, 25]
SNAPC2	small nuclear RNA activating complex, polypeptide 2	[13, 22]
SNED1	sushi, nidogen and EGF-like domains 1	[19, 22]
SNORA38	small nucleolar RNA, H/ACA box 38B (retrotransposed); small nucleolar RNA, H/ACA box 38	[22]
SNORD52	chromosome 6 open reading frame 48; small nucleolar RNA, C/D box 52	[13]
SNORD58A	U58 small nucleolar RNA; small nucleolar RNA, C/D box 58C; small nucleolar RNA, C/D box 58A; small nucleolar RNA, C/D box 58B	[13, 22]

SNORD78	small nucleolar RNA, C/D box 78	[13, 22]
SNORD93	small nucleolar RNA, C/D box 93	[13, 22]
SNX21	sorting nexin family member 21	[13]
SNX22	sorting nexin 22	[13]
SORBS1	sorbin and SH3 domain containing 1	[13]
SPIRE2	spire homolog 2 (Drosophila)	[16]
SPN	sialophorin	[13, 19]
STXBP4	syntaxin binding protein 4	[13, 22]
SUCLG2	succinate-CoA ligase, GDP-forming, beta subunit	[16]
SUMF2	sulfatase modifying factor 2	[13]
TAOK3	TAO kinase 3	[16]
TBC1D14	TBC1 domain family, member 14	[13]
TBCD	tubulin-specific chaperone d	[13]
TCEA2	transcription elongation factor A (SII), 2	[22]
TCP11	t-complex protein 11; tRNA splicing endonuclease 34 homolog (S. cerevisiae); similar to pBS13 precursor polypeptide, testis-specific; RIKEN cDNA 4930526A20 gene	[13, 16]
TERT	telomerase reverse transcriptase	[13, 25]
TG	thyroglobulin	[13]
TGFBI	transforming growth factor, beta induced	[9]
TGFBR3	transforming growth factor, beta receptor III	[13]
TIAM2	T-cell lymphoma invasion and metastasis 2	[13, 22]
TIGIT	T cell immunoreceptor with Ig and ITIM domains	[13, 22]
TLCD1	TLC domain containing 1	[12, 22]
TM4SF19	transmembrane 4 L six family member 19	[11, 12]
TMEM51	transmembrane protein 51	[13, 15, 19, 22]
TNF	tumor necrosis factor (TNF superfamily, member 2)	[13, 22]
TNIP2	TNFAIP3 interacting protein 2	[13, 22]
TNRC18	trinucleotide repeat containing 18	[16, 19, 21, 22]
TRERF1	transcriptional regulating factor 1	[13, 16]
TRIM26	tripartite motif-containing 26	[13, 16]
TRIM69	tripartite motif-containing 69	[13]
TRIO	similar to Trio splicing; triple functional domain (PTPRF interacting)	[22]
TSKU	tsukushin	[13, 22]
TWF2	twinfilin, actin-binding protein, homolog 2 (Drosophila)	[13, 16]
UNC80	hypothetical protein LOC779473	[16]
UXS1	UDP-glucuronate decarboxylase 1	[13, 22]
VARS	Sensor protein	[13, 15, 16, 18]
VPS52	vacuolar protein sorting 52 homolog (S. cerevisiae)	[13]
XYLT1	xylosyltransferase I	[19, 22]
YAP1	Yes-associated protein 1, 65kDa	[17]
ZBTB16	zinc finger and BTB domain containing 16	[13]
ZC3H12A	zinc finger CCCH-type containing 12A	[13, 22]

ZC3H12D	zinc finger CCCH-type containing 12D	[13, 22]
ZC3H3	zinc finger CCCH-type containing 3	[13, 22]
ZFHX3	zinc finger homeobox 3	[13]
ZFYVE21	zinc finger, FYVE domain containing 21	[13]
ZMIZ1	zinc finger, MIZ-type containing 1	[19, 22]
ZMYND8	zinc finger, MYND-type containing 8	[13, 22]
ZNF384	zinc finger protein 384	[12]
ZNF385D	zinc finger protein 385D	[13, 18]

Note: Based on the biomaterial of blood samples, here we present a total of 320 genes that were supported by two or more independent items of evidence (two-hint-based evidence); i.e., there are two or more significant smoking-associated DNAm loci in a gene, or there is only one significant DNAm locus in a gene replicated by two or more independent samples. [a]References: the included studies that reported significant loci within genes. The identified genes with a single reference represent the two-hint-based evidence were provided by the reference itself.

Supplemental Table S3: Genes enriched by SA-DNAm loci with at least two independent pieces of evidence from buccal brushings

Gene Symbol	Gene Name	References[a]
AATF	apoptosis antagonizing transcription factor	[28]
ABCC3	ATP-binding cassette, sub-family C (CFTR/MRP), member 3	[28]
ABO	ABO blood group (transferase A, alpha 1-3-N-acetylgalactosaminyltransferase; transferase B, alpha 1-3-galactosyltransferase)	[28]
ABR	active BCR-related gene	[28]
ABTB2	ankyrin repeat and BTB (POZ) domain containing 2	[28]
ACOT11	acyl-CoA thioesterase 11	[28]
ACSL6	acyl-CoA synthetase long-chain family member 6	[28]
ACTB	actin, beta	[28]
ACTN4	actinin, alpha 4	[28]
ACVR1	activin A receptor, type I	[28]
ADCY2	adenylate cyclase 2 (brain)	[28]
ADCY4	adenylate cyclase 4	[28]
ADCY9	adenylate cyclase 9	[28]
ADHFE1	alcohol dehydrogenase, iron containing, 1	[28]
ADORA2B	hypothetical LOC100131909; adenosine A2b receptor	[28]
ADRBK1	adrenergic, beta, receptor kinase 1	[28]
AGAP1	ArfGAP with GTPase domain, ankyrin repeat and PH domain 1	[28]
AHRR	aryl-hydrocarbon receptor repressor; programmed cell death 6	[28]
AK3L1	adenylate kinase 3-like 2; adenylate kinase 3-like 1	[28]
ALDH3A1	aldehyde dehydrogenase 3 family, memberA1	[28]
ALDOA	aldolase A, fructose-bisphosphate	[28]
ALS2CL	ALS2 C-terminal like	[28]
ANGPT4	angiopoietin 4	[28]
ANK2	ankyrin 2, neuronal	[28]
ANKRD33B	ankyrin repeat domain 33B	[28]
APBB1IP	amyloid beta (A4) precursor protein-binding, family B, member 1 interacting protein	[28]
ARHGAP22	Rho GTPase activating protein 22	[28]
ARHGDIA	Rho GDP dissociation inhibitor (GDI) alpha	[28]
ARHGEF1	Rho guanine nucleotide exchange factor (GEF) 1	[28]
ARHGEF10	Rho guanine nucleotide exchange factor (GEF) 10	[28]
ARHGEF4	Rho guanine nucleotide exchange factor (GEF) 4	[28]
ARID3A	AT rich interactive domain 3A (BRIGHT-like)	[28]
ARID3B	AT rich interactive domain 3B (BRIGHT-like)	[28]
ARL5C	ADP-ribosylation factor-like 5C	[28]
ARPP-21	cAMP-regulated phosphoprotein, 21kDa	[28]
ARTN	artemin	[28]

ASAP2	ArfGAP with SH3 domain, ankyrin repeat and PH domain 2	[28]
ASPSCR1	alveolar soft part sarcoma chromosome region, candidate 1	[28]
ASS1	argininosuccinate synthetase 1	[28]
ATL3	atlastin GTPase 3	[28]
ATOH8	atonal homolog 8 (Drosophila)	[28]
ATP5G2	ATP synthase, H+ transporting, mitochondrial F0 complex, subunit C2 (subunit 9)	[28]
ATP6V0A1	ATPase, H+ transporting, lysosomal V0 subunit a1	[28]
ATP6V1G3	ATPase, H+ transporting, lysosomal 13kDa, V1 subunit G3	[28]
AUTS2	autism susceptibility candidate 2	[28]
AVEN	apoptosis, caspase activation inhibitor	[28]
AVP	arginine vasopressin	[28]
B4GALNT1	beta-1,4-N-acetyl-galactosaminyl transferase 1	[28]
BAHCC1	BAH domain and coiled-coil containing 1	[28]
BAIAP2	BAI1-associated protein 2	[28]
BANP	BTG3 associated nuclear protein	[28]
BCC5	Basal cell carcinoma, susceptibility to, 5	[28]
BCL11B	B-cell CLL/lymphoma 11B (zinc finger protein)	[28]
BCL3	B-cell CLL/lymphoma 3	[28]
BCL7A	B-cell CLL/lymphoma 7A	[28]
BCOR	BCL6 co-repressor	[28]
BEND7	BEN domain containing 7	[28]
BICD2	bicaudal D homolog 2 (Drosophila)	[28]
BLMH	bleomycin hydrolase	[28]
BMI1	BMI1 polycomb ring finger oncogene	[28]
BMP7	bone morphogenetic protein 7	[28]
BMPR1A	bone morphogenetic protein receptor, type IA; similar to ALK-3	[28]
BST1	bone marrow stromal cell antigen 1	[28]
BTBD17	BTB (POZ) domain containing 17	[28]
BTBD7	BTB (POZ) domain containing 7	[28]
BZRAP1	benzodiazapine receptor (peripheral) associated protein 1	[28]
C10orf105	chromosome 10 open reading frame 105	[28]
C10orf41	chromosome 10 open reading frame 41	[28]
C10orf99	chromosome 10 open reading frame 99	[28]
C11orf49	chromosome 11 open reading frame 49	[28]
C11orf88	chromosome 11 open reading frame 88	[28]
C11orf95	hypothetical protein LOC65998	[28]
C14orf39	chromosome 14 open reading frame 39	[28]
C15orf63	chromosome 15 open reading frame 63; small EDRK-rich factor 2	[28]
C17orf46	chromosome 17 open reading frame 46	[28]
C19orf38	chromosome 19 open reading frame 38	[28]
C19orf55	chromosome 19 open reading frame 55	[28]
C1orf212	chromosome 1 open reading frame 212	[28]

C1QA	complement component 1, q subcomponent, A chain	[28]
C22orf15	chromosome 22 open reading frame 15	[28]
C2orf58	chromosome 2 open reading frame 58	[28]
C2orf76	chromosome 2 open reading frame 76	[28]
C3orf20	chromosome 3 open reading frame 20	[28]
C3orf21	chromosome 3 open reading frame 21	[28]
C5orf32	chromosome 5 open reading frame 32	[28]
C6orf114	chromosome 6 open reading frame 114	[28]
C7orf50	chromosome 7 open reading frame 50	[28]
C9orf3	chromosome 9 open reading frame 3	[28]
C9orf47	chromosome 9 open reading frame 47	[28]
C9orf86	chromosome 9 open reading frame 86	[28]
CACNA1D	calcium channel, voltage-dependent, L type, alpha 1D subunit	[28]
CACNA2D4	calcium channel, voltage-dependent, alpha 2/delta subunit 4	[28]
CALCOCO2	calcium binding and coiled-coil domain 2	[28]
CAMK2B	calcium/calmodulin-dependent protein kinase II beta	[28]
CAMK2D	calcium/calmodulin-dependent protein kinase II delta	[28]
CAMK2G	calcium/calmodulin-dependent protein kinase II gamma	[28]
CAPN10	calpain 10	[28]
CAPN2	calpain 2, (m/II) large subunit	[28]
CAPZA2	capping protein (actin filament) muscle Z-line, alpha 2	[28]
CASR	calcium-sensing receptor	[28]
CASZ1	castor zinc finger 1	[28]
CBLN1	cerebellin 1 precursor	[28]
CCDC140	coiled-coil domain containing 140	[28]
CCDC150	coiled-coil domain containing 150	[28]
CCDC81	coiled-coil domain containing 81	[28]
CCNDBP1	cyclin D-type binding-protein 1	[28]
CCNJL	cyclin J-like	[28]
CD300LG	CD300 molecule-like family member g	[28]
CD52	CD52 molecule	[28]
CD59	CD59 molecule, complement regulatory protein	[28]
CDC42EP3	CDC42 effector protein (Rho GTPase binding) 3	[28]
CDH16	cadherin 16, KSP-cadherin	[28]
CDH23	cadherin-like 23	[28]
CDH5	cadherin 5, type 2 (vascular endothelium)	[28]
CDK2AP1	cyclin-dependent kinase 2 associated protein 1	[28]
CDKN2C	cyclin-dependent kinase inhibitor 2C (p18, inhibits CDK4)	[28]
CDKN2D	cyclin-dependent kinase inhibitor 2D (p19, inhibits CDK4)	[28]
CDR2	cerebellar degeneration-related protein 2, 62kDa	[28]
CECR1	cat eye syndrome chromosome region, candidate 1	[28]
CES1	carboxylesterase 1 (monocyte/macrophage serine esterase 1)	[28]
CGN	cingulin	[28]

CHADL	chondroadherin-like	[28]
CHRM5	cholinergic receptor, muscarinic 5	[28]
CHSY1	chondroitin sulfate synthase 1	[28]
CIT	citron (rho-interacting, serine/threonine kinase 21)	[28]
CLCF1	cardiotrophin-like cytokine factor 1	[28]
CLDN15	claudin 15	[28]
CLMN	calmin (calponin-like, transmembrane)	[28]
CLRN1	clarin 1	[28]
CLYBL	citrate lyase beta like	[28]
CMTM2	CKLF-like MARVEL transmembrane domain containing 2	[28]
CNNM4	cyclin M4	[28]
COL18A1	collagen, type XVIII, alpha 1	[28]
COL7A1	collagen, type VII, alpha 1	[28]
CPT1A	carnitine palmitoyltransferase 1A (liver)	[28]
CREB3L1	cAMP responsive element binding protein 3-like 1	[28]
CRHR2	corticotropin releasing hormone receptor 2	[28]
CRIM1	cysteine rich transmembrane BMP regulator 1 (chordin-like)	[28]
CROCC	ciliary rootlet coiled-coil, rootletin	[28]
CRTAC1	cartilage acidic protein 1	[28]
CSNK1E	casein kinase 1, epsilon	[28]
CSRNP1	cysteine-serine-rich nuclear protein 1	[28]
CTAGE5	CTAGE family, member 5 pseudogene; CTAGE family member; CTAGE family, member 4; CTAGE family, member 5	[28]
CTBP1	C-terminal binding protein 1	[28]
CTBP2	C-terminal binding protein 2	[28]
CTNNBIP1	catenin, beta interacting protein 1	[28]
CUL1	cullin 1	[28]
CUX1	cut-like homeobox 1	[28]
CXCL12	chemokine (C-X-C motif) ligand 12 (stromal cell-derived factor 1)	[28]
CXXC5	CXXC finger 5	[28]
CYBASC3	cytochrome b, ascorbate dependent 3	[28]
CYFIP2	cytoplasmic FMR1 interacting protein 2	[28]
CYP1A1	cytochrome P450, family 1, subfamily A, polypeptide 1	[28]
CYP1B1	cytochrome P450, family 1, subfamily B, polypeptide 1	[27, 28]
CYP2W1	cytochrome P450, family 2, subfamily W, polypeptide 1	[28]
CYTSB	cytospin B	[28]
DACH1	dachshund homolog 1 (Drosophila)	[28]
DAGLA	diacylglycerol lipase, alpha	[28]
DCLK1	doublecortin-like kinase 1	[28]
DEFB132	defensin, beta 132	[28]
DGKZ	diacylglycerol kinase, zeta 104kDa	[28]
DHCR24	24-dehydrocholesterol reductase	[28]
DIDO1	death inducer-obliterator 1	[28]

DIP2C	DIP2 disco-interacting protein 2 homolog C (Drosophila)	[28]
DIS3L2	DIS3 mitotic control homolog (S. cerevisiae)-like 2	[28]
DLEU2	deleted in lymphocytic leukemia 2 (non-protein coding); deleted in lymphocytic leukemia 2-like	[28]
DLG4	discs, large homolog 4 (Drosophila)	[28]
DLX5	distal-less homeobox 5	[28]
DNAH1	dynein, axonemal, heavy chain 1	[28]
DNAH17	dynein, axonemal, heavy chain 17	[28]
DNAH2	dynein, axonemal, heavy chain 2	[28]
DNAJC6	DnaJ (Hsp40) homolog, subfamily C, member 6	[28]
DOCK6	dedicator of cytokinesis 6	[28]
DPH5	DPH5 homolog (S. cerevisiae)	[28]
DPP6	dipeptidyl-peptidase 6	[28]
DTX2	deltex homolog 2 (Drosophila)	[28]
DTX4	deltex homolog 4 (Drosophila)	[28]
DUSP16	dual specificity phosphatase 16	[28]
EBF3	early B-cell factor 3	[28]
ECE1	endothelin converting enzyme 1	[28]
ECE2	endothelin converting enzyme 2	[28]
EDC3	enhancer of mRNA decapping 3 homolog (S. cerevisiae)	[28]
EFEMP2	EGF-containing fibulin-like extracellular matrix protein 2	[28]
EHMT2	euchromatic histone-lysine N-methyltransferase 2	[28]
EIF4EBP1	eukaryotic translation initiation factor 4E binding protein 1	[28]
ELF1	E74-like factor 1 (ets domain transcription factor)	[28]
ELL2	elongation factor, RNA polymerase II, 2	[28]
EMX1	empty spiracles homeobox 1	[28]
ENOX1	ecto-NOX disulfide-thiol exchanger 1	[28]
EPB41L3	erythrocyte membrane protein band 4.1-like 3	[28]
EPHX1	epoxide hydrolase 1, microsomal (xenobiotic)	[28]
EPS8L2	EPS8-like 2	[28]
ERCC1	excision repair cross-complementing rodent repair deficiency, complementation group 1 (includes overlapping antisense sequence)	[28]
ERGIC1	endoplasmic reticulum-golgi intermediate compartment (ERGIC) 1	[28]
ETS2	v-ets erythroblastosis virus E26 oncogene homolog 2 (avian)	[28]
EXOC3	exocyst complex component 3	[28]
EXPH5	exophilin 5	[28]
F2RL3	coagulation factor II (thrombin) receptor-like 3	[28]
FAIM2	Fas apoptotic inhibitory molecule 2	[28]
FAM113B	family with sequence similarity 113, member B	[28]
FAM134B	family with sequence similarity 134, member B	[28]
FAM155A	family with sequence similarity 155, member A	[28]
FAM198B	chromosome 4 open reading frame 18	[28]
FAM19A1	family with sequence similarity 19 (chemokine (C-C motif)-like), member A1	[28]

FAM38A	family with sequence similarity 38, member A	[28]
FAM38B	family with sequence similarity 38, member B	[28]
FAM46A	family with sequence similarity 46, member A	[28]
FAM53A	family with sequence similarity 53, member A	[28]
FAM84B	family with sequence similarity 84, member B	[28]
FBLIM1	filamin binding LIM protein 1	[28]
FBN2	fibrillin 2	[28]
FBRSL1	fibrosin-like 1	[28]
FBXL18	F-box and leucine-rich repeat protein 18	[28]
FBXO2	F-box protein 2	[28]
FERMT1	fermitin family homolog 1 (Drosophila)	[28]
FGF18	fibroblast growth factor 18	[28]
FKBP4	FK506 binding protein 4, 59kDa	[28]
FLJ13197	hypothetical FLJ13197	[28]
FLJ23834	hypthetical protein FLJ23834	[28]
FLNB	filamin B, beta (actin binding protein 278)	[28]
FOXJ1	forkhead box J1	[28]
FOXK1	forkhead box K1	[28]
FOXR1	forkhead box R1	[28]
FREM3	FRAS1 related extracellular matrix 3	[28]
FRMD4A	FERM domain containing 4A	[27, 28]
FRMD4B	FERM domain containing 4B	[28]
FSCN1	fascin homolog 1, actin-bundling protein (Strongylocentrotus purpuratus)	[28]
FYN	FYN oncogene related to SRC, FGR, YES	[28]
FZD5	frizzled homolog 5 (Drosophila)	[28]
G0S2	G0/G1switch 2	[28]
GABRP	gamma-aminobutyric acid (GABA) A receptor, pi	[28]
GADD45A	growth arrest and DNA-damage-inducible, alpha	[28]
GAL3ST3	galactose-3-O-sulfotransferase 3	[28]
GFI1	growth factor independent 1 transcription repressor	[28]
GFOD1	glucose-fructose oxidoreductase domain containing 1	[28]
GLB1L3	galactosidase, beta 1-like 3	[28]
GLI2	GLI family zinc finger 2	[28]
GLI3	GLI family zinc finger 3	[28]
GLIS1	GLIS family zinc finger 1	[28]
GLTPD2	glycolipid transfer protein domain containing 2	[28]
GNG12	guanine nucleotide binding protein (G protein), gamma 12	[28]
GOT1L1	glutamic-oxaloacetic transaminase 1-like 1	[28]
GPR144	G protein-coupled receptor 144	[28]
GPR21	G protein-coupled receptor 21	[28]
GPRC5C	G protein-coupled receptor, family C, group 5, member C	[28]
GPX1	glutathione peroxidase 1	[28]
GPX2	glutathione peroxidase 2 (gastrointestinal)	[28]

GRAMD2	GRAM domain containing 2	[28]
GRIK3	glutamate receptor, ionotropic, kainate 3	[28]
GRK6	G protein-coupled receptor kinase 6	[28]
GSN	gelsolin (amyloidosis, Finnish type)	[28]
GUCA1A	guanylate cyclase activator 1A (retina)	[28]
GUCY1A2	guanylate cyclase 1, soluble, alpha 2	[28]
GUCY2E	guanylate cyclase 2E	[28]
GYS1	glycogen synthase 1 (muscle)	[28]
H2AFY	H2A histone family, member Y	[28]
H6PD	hexose-6-phosphate dehydrogenase (glucose 1-dehydrogenase)	[28]
HCG9	HLA complex group 9	[28]
HDAC4	histone deacetylase 4	[28]
HDGF2	hepatoma-derived growth factor-related protein 2	[28]
HERPUD1	homocysteine-inducible, endoplasmic reticulum stress-inducible, ubiquitin-like domain member 1	[28]
HIPK2	homeodomain interacting protein kinase 2; similar to homeodomain interacting protein kinase 2	[28]
HIST1H2BK	histone cluster 1, H2bk	[28]
HIST1H4I	histone cluster 1, H4l; histone cluster 1, H4k; histone cluster 4, H4; histone cluster 1, H4h; histone cluster 1, H4j; histone cluster 1, H4i; histone cluster 1, H4d; histone cluster 1, H4c; histone cluster 1, H4f; histone cluster 1, H4e; histone cluster 1, H4b; histone cluster 1, H4a; histone cluster 2, H4a; histone cluster 2, H4b	[28]
HIVEP1	human immunodeficiency virus type I enhancer binding protein 1	[28]
HIVEP2	human immunodeficiency virus type I enhancer binding protein 2	[28]
HIVEP3	human immunodeficiency virus type I enhancer binding protein 3	[28]
HLA-DOA	major histocompatibility complex, class II, DO alpha	[28]
HLF	hepatic leukemia factor	[28]
HNRNPA1	heterogeneous nuclear ribonucleoprotein A1-like 3; similar to heterogeneous nuclear ribonucleoprotein A1; heterogeneous nuclear ribonucleoprotein A1 pseudogene 2; heterogeneous nuclear ribonucleoprotein A1; heterogeneous nuclear ribonucleoprotein A1 pseudogene	[28]
HNRNPUL1	heterogeneous nuclear ribonucleoprotein U-like 1	[28]
HOXB4	homeobox B4	[28]
HOXC10	homeobox C10	[28]
HOXD3	homeobox D3	[28]
HSD17B6	hydroxysteroid (17-beta) dehydrogenase 6 homolog (mouse)	[28]
HSPB6	heat shock protein, alpha-crystallin-related, B6	[28]
HTR1B	5-hydroxytryptamine (serotonin) receptor 1B	[28]
HYAL3	hyaluronoglucosaminidase 3	[28]
IFI30	interferon, gamma-inducible protein 30	[28]
IKZF4	IKAROS family zinc finger 4 (Eos)	[28]
INCENP	inner centromere protein antigens 135/155kDa	[28]

INPPL1	inositol polyphosphate phosphatase-like 1	[28]
IRF8	interferon regulatory factor 8	[28]
ITGA2B	integrin, alpha 2b (platelet glycoprotein IIb of IIb/IIIa complex, antigen CD41)	[28]
ITGA5	integrin, alpha 5 (fibronectin receptor, alpha polypeptide)	[28]
ITPK1	inositol 1,3,4-triphosphate 5/6 kinase	[28]
ITPKB	inositol 1,4,5-trisphosphate 3-kinase B	[28]
JAK3	Janus kinase 3	[28]
JAKMIP1	Janus kinase and microtubule interacting protein 1	[28]
JAZF1	JAZF zinc finger 1	[28]
KCNG4	potassium voltage-gated channel, subfamily G, member 4	[28]
KCNIP2	Kv channel interacting protein 2	[28]
KCNIP3	Kv channel interacting protein 3, calsenilin	[28]
KCNQ1	potassium voltage-gated channel, KQT-like subfamily, member 1	[28]
KCNS1	potassium voltage-gated channel, delayed-rectifier, subfamily S, member 1	[28]
KDM2B	lysine (K)-specific demethylase 2B	[28]
KHK	ketohexokinase (fructokinase)	[28]
KIAA0182	KIAA0182	[28]
KIAA1026	kazrin, periplakin interacting protein	[28]
KIAA1199	KIAA1199	[28]
KIAA1543	KIAA1543	[28]
KIF13A	kinesin family member 13A	[28]
KIF1B	kinesin family member 1B	[28]
KIFC2	kinesin family member C2	[28]
KIRREL3	kin of IRRE like 3 (Drosophila)	[28]
KLF3	Kruppel-like factor 3 (basic)	[28]
KLHDC7B	kelch domain containing 7B	[28]
KNDC1	kinase non-catalytic C-lobe domain (KIND) containing 1	[28]
KREMEN2	kringle containing transmembrane protein 2	[28]
KRT7	keratin 7	[28]
KRTAP3-1	keratin associated protein 3-1	[28]
L3MBTL2	l(3)mbt-like 2 (Drosophila)	[28]
LAMA3	laminin, alpha 3	[28]
LASS2	LAG1 homolog, ceramide synthase 2	[28]
LHX1	LIM homeobox 1	[28]
LINC00673	Long intergenic non-protein coding RNA 673	[27]
LINGO3	leucine rich repeat and Ig domain containing 3	[28]
LIX1L	Lix1 homolog (mouse)-like	[28]
LMX1A	LIM homeobox transcription factor 1, alpha	[28]
LOC100129066	hypothetical LOC100129066	[28]
LOC100130691	hypothetical LOC100130691	[28]
LOC100130933	hypothetical LOC100130933	[28]
LOC100130987	similar to hCG1815675	[28]

LOC100133991	similar to hCG1995169	[28]
LOC100169752	hypothetical LOC100169752	[28]
LOC100192378	hypothetical LOC100192378	[28]
LOC100287216	hypothetical LOC100287216	[28]
LOC221122	hypothetical LOC221122	[28]
LOC283404	hypothetical LOC283404	[28]
LOC285830	hypothetical LOC285830	[28]
LOC286467	hypothetical LOC286467	[28]
LOC340074	hypothetical LOC340074	[28]
LOC404266	hypothetical LOC404266	[28]
LOC552889	hypothetical protein LOC552889	[28]
LOC644145	exocyst -like pseudogene	[28]
LOC730755	keratin associated protein 2-1; keratin associated protein 2-4; keratin associated protein 2-3; similar to keratin associated protein 2-4; keratin associated protein 2-2	[28]
LOXHD1	lipoxygenase homology domains 1	[28]
LOXL1	lysyl oxidase-like 1	[28]
LPO	lactoperoxidase	[28]
LPXN	leupaxin	[28]
LRIG1	leucine-rich repeats and immunoglobulin-like domains 1	[28]
LRP5	low density lipoprotein receptor-related protein 5	[28]
LRRFIP1	leucine rich repeat (in FLII) interacting protein 1	[28]
LSP1	lymphocyte-specific protein 1	[28]
MAD1L1	MAD1 mitotic arrest deficient-like 1 (yeast)	[28]
MAEA	macrophage erythroblast attacher	[28]
MAGI1	membrane associated guanylate kinase, WW and PDZ domain containing 1; CNKSR family member 3	[28]
MAGI2	membrane associated guanylate kinase, WW and PDZ domain containing 2	[28]
MAML3	mastermind-like 3 (Drosophila)	[28]
MAN1C1	mannosidase, alpha, class 1C, member 1	[28]
MAST3	microtubule associated serine/threonine kinase 3	[28]
MCF2L	MCF.2 cell line derived transforming sequence-like	[28]
MCHR1	melanin-concentrating hormone receptor 1	[28]
MCM2	minichromosome maintenance complex component 2	[28]
MDFI	MyoD family inhibitor	[28]
ME3	malic enzyme 3, NADP(+)-dependent, mitochondrial	[28]
MEF2A	myocyte enhancer factor 2A	[28]
MEF2C	myocyte enhancer factor 2C	[28]
MEIS2	Meis homeobox 2	[28]
MEOX2	mesenchyme homeobox 2	[28]
MFAP4	microfibrillar-associated protein 4	[28]
MGC2752	hypothetical LOC65996	[28]
MINA	MYC induced nuclear antigen	[28]

MIR1204	microRNA 1204	[28]
MIR129-2	microRNA 129-2	[28]
MIR130A	microRNA 130a	[28]
MIR146B	microRNA 146b	[28]
MIR23A	microRNA 23a	[28]
MIR24-1	microRNA 24-1	[28]
MIR24-2	microRNA 24-2	[28]
MIR27A	microRNA 27a	[28]
MIR377	microRNA 377	[28]
MIR548F5	microRNA 548F-5	[28]
MKNK2	MAP kinase interacting serine/threonine kinase 2	[28]
MKX	mohawk homeobox	[28]
MMP23A	matrix metallopeptidase 23A (pseudogene); matrix metallopeptidase 23B	[28]
MMP23B	matrix metallopeptidase 23A (pseudogene); matrix metallopeptidase 23B	[28]
MPG	N-methylpurine-DNA glycosylase	[28]
MPPED1	metallophosphoesterase domain containing 1	[28]
MPPED2	metallophosphoesterase domain containing 2	[28]
MSI1	musashi homolog 1 (Drosophila)	[28]
MSI2	musashi homolog 2 (Drosophila)	[28]
MSRB3	methionine sulfoxide reductase B3	[28]
MT1A	metallothionein 1A	[28]
MT1IP	metallothionein 1I (pseudogene)	[28]
MTHFD1L	methylenetetrahydrofolate dehydrogenase (NADP+dependent) 1-like	[28]
MTL5	metallothionein-like 5, testis-specific (tesmin)	[28]
MTMR9L	myotubularin related protein 9 pseudogene	[28]
MUC2	mucin 2, oligomeric mucus/gel-forming	[28]
MYOM3	myomesin family, member 3	[28]
MYT1L	myelin transcription factor 1-like	[28]
NAT6	N-acetyltransferase 6 (GCN5-related)	[28]
NBEAL2	neurobeachin-like 2	[28]
NCF4	neutrophil cytosolic factor 4, 40kDa	[28]
NCOR2	nuclear receptor co-repressor 2	[28]
NDST1	N-deacetylase/N-sulfotransferase (heparan glucosaminyl) 1	[28]
NEFM	neurofilament, medium polypeptide	[28]
NEK3	NIMA (never in mitosis gene a)-related kinase 3	[28]
NEUROG1	neurogenin 1	[28]
NFAM1	NFAT activating protein with ITAM motif 1	[28]
NFAT5	nuclear factor of activated T-cells 5, tonicity-responsive	[28]
NHLRC4	hypothetical protein FLJ36208; chromosome 16 open reading frame 11	[28]
NKD2	naked cuticle homolog 2 (Drosophila)	[28]
NKX1-2	NK1 homeobox 2	[28]
NOTCH4	Notch homolog 4 (Drosophila)	[28]
NQO1	NAD(P)H dehydrogenase, quinone 1	[28]

NR2E1	nuclear receptor subfamily 2, group E, member 1	[28]
NRG2	neuregulin 2	[28]
NRXN1	neurexin 1	[28]
NT5E	5'-nucleotidase, ecto (CD73)	[28]
NTF3	neurotrophin 3	[28]
NTN1	netrin 1	[28]
NUP43	nucleoporin 43kDa	[28]
NXN	nucleoredoxin	[28]
NXPH4	neurexophilin 4	[28]
OCA2	oculocutaneous albinism II	[28]
ODC1	ornithine decarboxylase 1	[28]
ODZ4	odz, odd Oz/ten-m homolog 4 (Drosophila)	[28]
OSBPL9	oxysterol binding protein-like 9	[28]
OSCP1	chromosome 1 open reading frame 102	[28]
P2RY2	purinergic receptor P2Y, G-protein coupled, 2	[28]
PALLD	palladin, cytoskeletal associated protein	[28]
PAM	peptidylglycine alpha-amidating monooxygenase	[28]
PANK4	pantothenate kinase 4	[28]
PANX2	pannexin 2	[28]
PARD3B	par-3 partitioning defective 3 homolog B (C. elegans)	[28]
PARVA	parvin, alpha	[27, 28]
PAX7	paired box 7	[28]
PC	pyruvate carboxylase	[28]
PCGF3	polycomb group ring finger 3	[28]
PDE4D	phosphodiesterase 4D, cAMP-specific (phosphodiesterase E3 dunce homolog, Drosophila)	[28]
PER1	period homolog 1 (Drosophila)	[28]
PGAP2	post-GPI attachment to proteins 2	[28]
PGLYRP4	peptidoglycan recognition protein 4	[28]
PHF1	PHD finger protein 1	[28]
PHOX2A	paired-like homeobox 2a	[28]
PIGV	phosphatidylinositol glycan anchor biosynthesis, class V	[28]
PINX1	PIN2-interacting protein 1	[28]
PIR	pirin (iron-binding nuclear protein)	[28]
PITPNC1	phosphatidylinositol transfer protein, cytoplasmic 1	[28]
PKNOX2	PBX/knotted 1 homeobox 2	[28]
PLCB4	phospholipase C, beta 4	[28]
PLCH1	phospholipase C, eta 1	[28]
PLEC1	plectin 1	[28]
PLEKHA6	pleckstrin homology domain containing, family A member 6	[28]
PLXND1	plexin D1	[28]
PNPLA1	patatin-like phospholipase domain containing 1	[28]
POLG	polymerase (DNA directed), gamma	[28]

POLK	polymerase (DNA directed) kappa	[28]
POLR1A	polymerase (RNA) I polypeptide A, 194kDa	[28]
POU2F3	POU class 2 homeobox 3	[28]
PPBP	pro-platelet basic protein (chemokine (C-X-C motif) ligand 7)	[28]
PPP1R16B	protein phosphatase 1, regulatory (inhibitor) subunit 16B	[28]
PRDM1	PR domain containing 1, with ZNF domain	[28]
PRDM15	PR domain containing 15	[28]
PRDM16	PR domain containing 16	[28]
PRDM6	PR domain containing 6	[28]
PRKCZ	protein kinase C, zeta	[28]
PROM2	prominin 2	[28]
PRPH2	peripherin 2 (retinal degeneration, slow)	[28]
PRRT4	hypothetical protein LOC401399	[28]
PRRX1	paired related homeobox 1	[28]
PRSS27	protease, serine 27	[28]
PRX	periaxin	[28]
PSD2	pleckstrin and Sec7 domain containing 2	[28]
PTCRA	pre T-cell antigen receptor alpha	[28]
PTPRF	protein tyrosine phosphatase, receptor type, F	[28]
PTPRN	protein tyrosine phosphatase, receptor type, N	[28]
PTPRN2	protein tyrosine phosphatase, receptor type, N polypeptide 2	[28]
PVRL1	poliovirus receptor-related 1 (herpesvirus entry mediator C)	[28]
PVT1	Pvt1 oncogene (non-protein coding)	[28]
PXK	PX domain containing serine/threonine kinase	[28]
QDPR	quinoid dihydropteridine reductase	[28]
RAB25	RAB25, member RAS oncogene family	[28]
RAB31	RAB31, member RAS oncogene family	[28]
RABGAP1	RAB GTPase activating protein 1	[28]
RAC2	ras-related C3 botulinum toxin substrate 2 (rho family, small GTP binding protein Rac2)	[28]
RAD51L1	RAD51-like 1 (S. cerevisiae)	[28]
RAG1AP1	recombination activating gene 1 activating protein 1	[28]
RAI1	retinoic acid induced 1	[28]
RAPGEFL1	Rap guanine nucleotide exchange factor (GEF)-like 1	[28]
RARA	retinoic acid receptor, alpha	[28]
RASGRP4	RAS guanyl releasing protein 4	[28]
RASIP1	Ras interacting protein 1	[28]
RBMS1	RNA binding motif, single stranded interacting protein 1	[28]
RCBTB2	regulator of chromosome condensation (RCC1) and BTB (POZ) domain containing protein 2	[28]
REC8	REC8 homolog (yeast)	[28]
RECQL5	RecQ protein-like 5	[28]
REEP4	receptor accessory protein 4	[28]

RGS12	regulator of G-protein signaling 12	[28]
RGS22	regulator of G-protein signaling 22	[28]
RHOBTB1	Rho-related BTB domain containing 1	[28]
RHOBTB3	Rho-related BTB domain containing 3	[28]
RNF130	ring finger protein 130	[28]
RNF216	ring finger protein 216	[28]
ROR2	receptor tyrosine kinase-like orphan receptor 2	[28]
RPLP2	ribosomal protein, large, P2 pseudogene 3; ribosomal protein, large, P2	[28]
RRN3P2	RRN3 RNA polymerase I transcription factor homolog (S. cerevisiae) pseudogene	[28]
RTKN2	rhotekin 2	[28]
RTN4RL1	reticulon 4 receptor-like 1	[28]
RUNX1	runt-related transcription factor 1	[28]
RXRA	retinoid X receptor, alpha	[28]
RYR1	ryanodine receptor 1 (skeletal)	[28]
S100B	S100 calcium binding protein B	[28]
S1PR3	sphingosine-1-phosphate receptor 3	[28]
SBNO2	strawberry notch homolog 2 (Drosophila)	[28]
SCT	secretin	[28]
SDCCAG8	serologically defined colon cancer antigen 8	[28]
SEC14L3	SEC14-like 3 (S. cerevisiae)	[28]
SECTM1	secreted and transmembrane 1	[28]
SELPLG	selectin P ligand	[28]
SEMA4A	sema domain, immunoglobulin domain (Ig), transmembrane domain (TM) and short cytoplasmic domain, (semaphorin) 4A	[28]
SEMA6D	sema domain, transmembrane domain (TM), and cytoplasmic domain, (semaphorin) 6D	[28]
SEPT9	septin 9	[28]
SERINC4	serine incorporator 4	[28]
SERINC5	serine incorporator 5	[28]
SERPINB1	serpin peptidase inhibitor, clade B (ovalbumin), member 1	[28]
SERPINB13	serpin peptidase inhibitor, clade B (ovalbumin), member 13	[28]
SERPINB6	serpin peptidase inhibitor, clade B (ovalbumin), member 6	[28]
SERTAD3	SERTA domain containing 3	[28]
SEZ6L	seizure related 6 homolog (mouse)-like	[28]
SFRP4	secreted frizzled-related protein 4	[28]
SFRS8	splicing factor, arginine/serine-rich 8 (suppressor-of-white-apricot homolog, Drosophila)	[28]
SGIP1	SH3-domain GRB2-like (endophilin) interacting protein 1	[28]
SGPP2	sphingosine-1-phosphate phosphotase 2	[28]
SH3GL1	SH3-domain GRB2-like 1	[28]
SH3RF3	SH3 domain containing ring finger 3	[28]
SKI	v-ski sarcoma viral oncogene homolog (avian)	[28]

SLC11A1	solute carrier family 11 (proton-coupled divalent metal ion transporters), member 1	[28]
SLC12A7	solute carrier family 12 (potassium/chloride transporters), member 7	[28]
SLC13A5	solute carrier family 13 (sodium-dependent citrate transporter), member 5	[28]
SLC16A11	solute carrier family 16, member 11 (monocarboxylic acid transporter 11)	[28]
SLC22A18AS	solute carrier family 22 (organic cation transporter), member 18 antisense	[28]
SLC24A3	solute carrier family 24 (sodium/potassium/calcium exchanger), member 3	[28]
SLC25A13	solute carrier family 25, member 13 (citrin)	[28]
SLC25A20	solute carrier family 25 (carnitine/acylcarnitine translocase), member 20	[28]
SLC25A42	solute carrier family 25, member 42	[28]
SLC29A1	solute carrier family 29 (nucleoside transporters), member 1	[28]
SLC2A3	solute carrier family 2 (facilitated glucose transporter), member 3	[28]
SLC3A1	solute carrier family 3 (cystine, dibasic and neutral amino acid transporters, activator of cystine, dibasic and neutral amino acid transport), member 1	[28]
SLC7A5	solute carrier family 7 (cationic amino acid transporter, y+ system), member 5	[28]
SLC8A1	solute carrier family 8 (sodium/calcium exchanger), member 1	[28]
SLC9A3R2	solute carrier family 9 (sodium/hydrogen exchanger), member 3 regulator 2	[28]
SLMO1	slowmo homolog 1 (Drosophila)	[28]
SMAD6	SMAD family member 6	[28]
SMARCD3	SWI/SNF related, matrix associated, actin dependent regulator of chromatin, subfamily d, member 3	[28]
SMOC1	SPARC related modular calcium binding 1	[28]
SND1	staphylococcal nuclease and tudor domain containing 1	[28]
SNED1	sushi, nidogen and EGF-like domains 1	[28]
SNORA52	small nucleolar RNA, H/ACA box 52	[28]
SNORD53	small nucleolar RNA, C/D box 53	[28]
SNX25	sorting nexin 25	[28]
SOCS2	suppressor of cytokine signaling 2	[28]
SORBS2	sorbin and SH3 domain containing 2	[28]
SOST	sclerosteosis	[28]
SP5	Sp5 transcription factor	[28]
SPARC	secreted protein, acidic, cysteine-rich (osteonectin)	[28]
SPATA18	spermatogenesis associated 18 homolog (rat)	[28]
SPATC1	spermatogenesis and centriole associated 1	[28]
SPEN	spen homolog, transcriptional regulator (Drosophila)	[28]
SPTBN1	spectrin, beta, non-erythrocytic 1	[28]
SRC	v-src sarcoma (Schmidt-Ruppin A-2) viral oncogene homolog (avian)	[28]
SSH1	slingshot homolog 1 (Drosophila)	[28]
SSU72	SSU72 RNA polymerase II CTD phosphatase homolog (S. cerevisiae)	[28]
ST3GAL6	ST3 beta-galactoside alpha-2,3-sialyltransferase 6	[28]
ST6GALNAC6	ST6 (alpha-N-acetyl-neuraminyl-2,3-beta-galactosyl-1,3)-N-acetylgalactosaminide alpha-2,6-sialyltransferase 6	[28]

ST7	suppression of tumorigenicity 7	[28]
STAMBPL1	STAM binding protein-like 1	[28]
STAT5B	signal transducer and activator of transcription 5B	[28]
STEAP3	STEAP family member 3	[28]
STK33	serine/threonine kinase 33	[28]
STK39	serine threonine kinase 39 (STE20/SPS1 homolog, yeast)	[28]
STXBP5-AS1	Syntaxin binding protein 5 antisense RNA 1	[27]
SURF6	surfeit 6	[28]
SYN2	synapsin II	[28]
SYNGAP1	synaptic Ras GTPase activating protein 1 homolog (rat)	[28]
SYNJ2	synaptojanin 2	[28]
SYT17	synaptotagmin XVII; synaptotagmin VII	[28]
TADA2B	transcriptional adaptor 2 (ADA2 homolog, yeast)-beta	[28]
TBC1D2	TBC1 domain family, member 2	[28]
TBX5	T-box 5	[28]
TBXAS1	thromboxane A synthase 1 (platelet)	[28]
TCEA3	transcription elongation factor A (SII), 3	[28]
TCF15	transcription factor 15 (basic helix-loop-helix)	[28]
TCHH	trichohyalin	[28]
TCN2	transcobalamin II; macrocytic anemia	[28]
TCTEX1D1	Tctex1 domain containing 1	[28]
TEAD3	TEA domain family member 3	[28]
TEAD4	TEA domain family member 4	[28]
TECR	glycoprotein, synaptic 2	[28]
TET1	tet oncogene 1	[28]
TFF2	trefoil factor 2	[28]
TGFB3	transforming growth factor, beta 3	[28]
TGFBR2	transforming growth factor, beta receptor II (70/80kDa)	[28]
TGM3	transglutaminase 3 (E polypeptide, protein-glutamine-gamma-glutamyltransferase)	[28]
TGM6	transglutaminase 6	[28]
THSD7B	thrombospondin, type I, domain containing 7B	[28]
TIMP4	TIMP metallopeptidase inhibitor 4	[28]
TJAP1	tight junction associated protein 1 (peripheral)	[28]
TK2	thymidine kinase 2, mitochondrial	[28]
TLL1	tolloid-like 1	[28]
TLX2	T-cell leukemia homeobox 2	[28]
TMEM111	transmembrane protein 111	[28]
TMEM168	transmembrane protein 168	[28]
TMEM2	transmembrane protein 2	[28]
TMPRSS2	transmembrane protease, serine 2	[28]
TNFAIP8	tumor necrosis factor, alpha-induced protein 8	[28]
TNNT3	troponin T type 3 (skeletal, fast)	[28]

TOX3	TOX high mobility group box family member 3	[28]
TP73	tumor protein p73	[28]
TPH2	tryptophan hydroxylase 2	[28]
TPPP	tubulin polymerization promoting protein	[28]
TPST1	tyrosylprotein sulfotransferase 1	[28]
TRABD	TraB domain containing	[28]
TRIM13	tripartite motif-containing 13	[28]
TRIO	triple functional domain (PTPRF interacting)	[28]
TRNP1	TMF1-regulated nuclear protein 1	[28]
TSC22D2	TSC22 domain family, member 2	[28]
TSLP	thymic stromal lymphopoietin	[28]
TSPAN9	tetraspanin 9	[28]
TTYH3	tweety homolog 3 (Drosophila)	[28]
TXNRD1	thioredoxin reductase 1; hypothetical LOC100130902	[28]
UBE2O	ubiquitin-conjugating enzyme E2O	[28]
UBTF	upstream binding transcription factor, RNA polymerase I	[28]
UBXN11	UBX domain protein 11	[28]
UCN2	urocortin 2	[28]
UGT1A1	UDP glucuronosyltransferase 1 family, polypeptide A1	[28]
UGT1A10	UDP glucuronosyltransferase 1 family, polypeptide A10	[28]
UGT1A3	UDP glucuronosyltransferase 1 family, polypeptide A3	[28]
UGT1A4	UDP glucuronosyltransferase 1 family, polypeptide A4	[28]
UGT1A5	UDP glucuronosyltransferase 1 family, polypeptide A5	[28]
UGT1A6	UDP glucuronosyltransferase 1 family, polypeptide A6	[28]
UGT1A7	UDP glucuronosyltransferase 1 family, polypeptide A7	[28]
UGT1A8	UDP glucuronosyltransferase 1 family, polypeptide A8	[28]
UGT1A9	UDP glucuronosyltransferase 1 family, polypeptide A9	[28]
UHRF1BP1L	UHRF1 binding protein 1-like	[28]
UNC119B	unc-119 homolog B (C. elegans)	[28]
UNKL	unkempt homolog (Drosophila)-like	[28]
USH1G	Usher syndrome 1G (autosomal recessive)	[28]
USP20	ubiquitin specific peptidase 20	[28]
VARS	valyl-tRNA synthetase	[28]
VNN2	vanin 2	[28]
VTCN1	V-set domain containing T cell activation inhibitor 1	[28]
WDR43	WD repeat domain 43	[28]
WNT10B	wingless-type MMTV integration site family, member 10B	[28]
6-Mar	hypothetical protein	[28]
ZC3H3	zinc finger CCCH-type containing 3	[28]
ZCCHC14	zinc finger, CCHC domain containing 14	[28]
ZDHHC4	zinc finger, DHHC-type containing 4	[28]
ZHX2	zinc fingers and homeoboxes 2	[28]
ZIC5	Zic family member 5 (odd-paired homolog, Drosophila)	[28]

ZMYM4	zinc finger, MYM-type 4	[28]
ZNF250	zinc finger protein 250	[28]
ZNF385D	zinc finger protein 385D	[28]
ZNF491	zinc finger protein 491	[28]
ZNF69	zinc finger protein 69	[28]
ZNF710	zinc finger protein 710	[28]
ZNF814	zinc finger protein 814	[28]
ZNF83	zinc finger protein 83	[28]
ZNRF3	zinc and ring finger 3	[28]
ZSCAN1	zinc finger and SCAN domain containing 1	[28]

Note: Based on the biomaterial of buccal samples, here we present a total of 661 genes that were supported by two or more independent items of evidence (two-hint-based evidence); i.e., there are two or more significant smoking-associated DNAm loci in a gene, or there is only one significant DNAm locus in a gene replicated by two or more independent samples. [a]References: the included studies that reported significant loci within genes. The identified genes with a single reference represent the two-hint-based evidence were provided by the reference itself.

Supplemental Table S4: All overrepresented pathways by SA-DNAm-related genes from blood samples[a]

Canonical Pathways	P-value	FDR	Genes Included[d]
MSP-RON Signaling Pathway	6.17×10^{-07}	0.00022	*ITGAM, IL3, PIK3CD, ACTB, RPS6KA2, TNF, PIK3R5, PRKCZ*
RAR Activation	2.04×10^{-06}	0.00037	*NCOR2, AKT3, RARA, ZBTB16, SMAD6, RXRB, SMARCA4, NR2F6, PIK3CD, ACTB, PRKAR1B, RARG, PRKCZ, ADCY9*
Rac Signaling	6.17×10^{-06}	0.00071	*BAIAP2, PIK3CD, PARD3, PIK3R5, MCF2L, PIP5K1C, PAK4, PTK2, PRKCZ, PIP4K2A*
Actin Cytoskeleton Signaling[b]	7.94×10^{-06}	0.00071	*GNA12, PIK3R5, MYH10, FGF23, PTK2, PIP4K2A, BAIAP2, PIK3CD, ACTB, TRIO, GNG12, PIP5K1C, PAK4, TIAM2*
Aryl Hydrocarbon Receptor Signaling	1.15×10^{-05}	0.00083	*NCOR2, AHRR, RARA, TNF, CYP1A1, CDKN1A, RXRB, SMARCA4, CDK6, RARG, NFE2L2*
Signaling by Rho Family GTPases	2.51×10^{-05}	0.00151	*ARHGEF3, PARD3, GNA12, PIK3R5, PTK2, PIP4K2A, BAIAP2, PIK3CD, ACTB, SEPT9, GNG12, PIP5K1C, PAK4, PRKCZ*
AMPK Signaling	2.95×10^{-05}	0.00155	*CRTC2, ATF4, AKT3, PIK3CD, RPTOR, ACTB, CDKN1A, PIK3R5, PRKAR1B, SMARCA4, ADRA2A, ACACA*
Renin-Angiotensin Signaling	6.03×10^{-05}	0.00275	*PIK3CD, TNF, PIK3R5, PRKAR1B, PTPN6, PAK4, PTK2, PRKCZ, ADCY9*
Molecular Mechanisms of Cancer[b]	7.41×10^{-05}	0.00295	*LRP5, ARHGEF3, AKT3, NOTCH1, GNA12, SMAD6, PIK3R5, PTK2, PIK3CD, PSEN2, CDKN1A, PRKAR1B, PAK4, CDK6, PRKCZ, PMAIP1, ADCY9*

CXCR4 Signaling	0.00017	0.00575	*AKT3, PIK3CD, GNA12, PIK3R5, GNG12, EGR1, PAK4, PTK2, PRKCZ, ADCY9*
Dorso-Ventral Axis Formation[c]	0.00019	0.0088	*ETS1, ETS2, ETV6, NOTCH1, SPIRE2*
ERK/MAPK Signaling	0.00021	0.00575	*KSR1, ATF4, PIK3CD, PIK3R5, PRKAR1B, ETS1, PAK4, PTK2, NFATC1, DUSP4, ETS2*
HER-2 Signaling in Breast Cancer	0.00021	0.00575	*AKT3, PIK3CD, PARD3, CDKN1A, PIK3R5, CDK6, PRKCZ*
Tec Kinase Signaling	0.00021	0.00575	*PIK3CD, ACTB, TNF, GNA12, PIK3R5, GNG12, FGR, PAK4, PTK2, PRKCZ*
Thrombin Signaling	0.00022	0.00575	*GATA3, ARHGEF3, AKT3, PIK3CD, GNA12, PIK3R5, GNG12, PTK2, F2RL3, PRKCZ, ADCY9*
HGF Signaling	0.00027	0.00603	*AKT3, PIK3CD, CDKN1A, PIK3R5, ETS1, PTK2, PRKCZ, ETS2*
Relaxin Signaling	0.00028	0.00603	*AKT3, PIK3CD, MMP9, GNA12, PIK3R5, GNG12, PRKAR1B, PRKCZ, ADCY9*
Leukocyte Extravasation Signaling[b]	0.00030	0.00603	*ITGAM, NCF4, SPN, ITGAL, PIK3CD, MMP9, ACTB, PIK3R5, PTK2, PRKCZ, RASSF5*
Huntington's Disease Signaling	0.00030	0.00603	*NCOR2, ATF4, AKT3, PIK3CD, HTT, HAP1, SDHA, AP2A2, PIK3R5, GNG12, PRKCZ, RPH3A*
Role of Tissue Factor in Cancer	0.00033	0.00631	*AKT3, PIK3CD, RPS6KA2, GNA12, PIK3R5, FGR, EGR1, ARRB1*
Melanocyte Development and Pigmentation Signaling	0.00040	0.00724	*ATF4, PIK3CD, RPS6KA2, PIK3R5, PRKAR1B, PTPN6, ADCY9*
TR/RXR Activation	0.00043	0.00741	*NCOR2, LDLR, AKT3, PIK3CD, PIK3R5, RXRB, ACACA*
B Cell Receptor Signaling[b]	0.00045	0.00741	*ATF4, AKT3, PIK3CD, PIK3R5, PTPN6, EGR1, ETS1, PTK2, NFATC1, RASSF5*
Non-Small Cell Lung Cancer Signaling[b]	0.00060	0.00955	*AKT3, PIK3CD, PIK3R5, RXRB, CDK6, RASSF5*
P2Y Purigenic Receptor Signaling Pathway	0.00063	0.00955	*ATF4, AKT3, PIK3CD, PIK3R5, GNG12, PRKAR1B, PRKCZ, ADCY9*
G-Protein Coupled Receptor Signaling	0.00085	0.0120	*ATF4, AKT3, PIK3CD, PIK3R5, HRH1, PRKAR1B, ADRA2A, CNR2, AVPR1B, RGS12, ADCY9, DUSP4*
Telomerase Signaling	0.00089	0.0120	*AKT3, PIK3CD, CDKN1A, PIK3R5, ETS1, TERT, ETS2*
IL-3 Signaling	0.00098	0.0120	*IL3, AKT3, PIK3CD, PIK3R5, PTPN6, PRKCZ*
Small Cell Lung Cancer Signaling[b]	0.00098	0.0120	*AKT3, PIK3CD, PIK3R5, RXRB, CDK6, PTK2*
Hereditary Breast Cancer Signaling	0.00098	0.0120	*AKT3, PIK3CD, ACTB, CDKN1A, PIK3R5, BRCA2, SMARCA4, CDK6*
Chemokine Signaling Pathway[c]	0.00098	0.0185	*ADCY9, AKT3, ARRB1, CXCR5, FGR, GNG12, PARD3, PIK3CD, PIK3R5, PRKCZ, PTK2, TIAM2*
Paxillin Signaling	0.00102	0.0123	*ITGAM, ITGAL, PIK3CD, ACTB, PIK3R5, PAK4, PTK2*
NF-κB Activation by Viruses	0.00112	0.0126	*ITGAL, AKT3, PIK3CD, CXCR5, PIK3R5, PRKCZ*
IL-4 Signaling	0.00112	0.0126	*HMGA1, AKT3, PIK3CD, PIK3R5, PTPN6, NFATC1*

Pancreatic Adenocarcinoma Signaling[b]	0.00158	0.0170	*AKT3, PIK3CD, NOTCH1, MMP9, CDKN1A, PIK3R5, BRCA2*
NGF Signaling	0.00158	0.0170	*ATF4, AKT3, PIK3CD, RPS6KA2, TRIO, PIK3R5, PRKCZ*
Folate biosynthesis[c]	0.00158	0.0208	*ALPI, ALPP, ALPPL2*
CREB Signaling in Neurons	0.00166	0.0170	*ATF4, AKT3, PIK3CD, GNA12, PIK3R5, GNG12, PRKAR1B, PRKCZ, ADCY9*
Reelin Signaling in Neurons	0.00170	0.0170	*ARHGEF3, ITGAL, PIK3CD, PIK3R5, FGR, CNR2*
Ceramide Signaling	0.00182	0.0174	*KSR1, AKT3, PIK3CD, TNF, PIK3R5, PRKCZ*
EIF2 Signaling	0.00186	0.0178	*AKT3, PIK3CD, RPS18, RPL35, RPL3, PPP1R15A, PIK3R5, AGO2, EIF4G1*
Epithelial Adherens Junction Signaling	0.00219	0.0204	*BAIAP2, AKT3, PARD3, NOTCH1, ACTB, SORBS1, TGFBR3, MYH10*
ErbB4 Signaling	0.00234	0.0204	*PIK3CD, PSEN2, PIK3R5, YAP1, PRKCZ*
Gαq Signaling	0.00240	0.0204	*AKT3, PIK3CD, PIK3R5, GNG12, HRH1, NFATC1, AVPR1B, PRKCZ*
FGF Signaling	0.00245	0.0204	*ATF4, AKT3, PIK3CD, PIK3R5, PTPN6, FGF23*
FAK Signaling	0.00257	0.0204	*AKT3, PIK3CD, ACTB, PIK3R5, PAK4, PTK2*
ILK Signaling	0.00263	0.0204	*ATF4, AKT3, PIK3CD, MMP9, ACTB, TNF, PIK3R5, MYH10, PTK2*
14-3-3-mediated Signaling	0.00269	0.0204	*AKT3, PIK3CD, TNF, PIK3R5, YAP1, EDC3, PRKCZ*
Antiproliferative Role of Somatostatin Receptor 2	0.00275	0.0204	*PIK3CD, CDKN1A, PIK3R5, GNG12, PTPN6*
mTOR Signaling[b]	0.00275	0.0204	*AKT3, PIK3CD, RPTOR, RPS18, RPS6KA2, DDIT4, PIK3R5, PRKCZ, EIF4G1*
Regulation of the Epithelial-Mesenchymal Transition Pathway	0.00275	0.0204	*AKT3, PIK3CD, NOTCH1, MMP9, PSEN2, PIK3R5, EGR1, ETS1, FGF23*
IL-8 Signaling	0.00282	0.0209	*ITGAM, AKT3, PIK3CD, MMP9, GNA12, PIK3R5, GNG12, PTK2, PRKCZ*
PTEN Signaling	0.00295	0.0209	*AKT3, PIK3CD, TGFBR3, CDKN1A, PIK3R5, PTK2, PRKCZ*
Gap Junction Signaling	0.00309	0.0209	*AKT3, PIK3CD, ACTB, CSNK1G3, PIK3R5, PRKAR1B, PRKCZ, ADCY9*
Virus Entry via Endocytic Pathways	0.00309	0.0209	*ITGAL, PIK3CD, ACTB, AP2A2, PIK3R5, PRKCZ*
VEGF Signaling[b]	0.00309	0.0209	*AKT3, PIK3CD, ACTB, PIK3R5, PTPN6, PTK2*
RhoA Signaling	0.00324	0.0219	*BAIAP2, ACTB, GNA12, SEPT9, PIP5K1C, PTK2, PIP4K2A*
Hematopoietic Cell Lineage[c]	0.00336	0.0383	*ANPEP, CD38, CD59, GP5, IL3, ITGAM, TNF*
Estrogen-Dependent Breast Cancer Signaling	0.00339	0.0224	*ATF4, AKT3, PIK3CD, PIK3R5, TERT*
Axonal Guidance Signaling	0.00380	0.0245	*AKT3, MMP9, GNA12, PIK3R5, NFATC1, PTK2, RASSF5, BAIAP2, PIK3CD, SEMA7A, GNG12,*

			PRKAR1B, PAK4, FES, PRKCZ
Angiopoietin Signaling	0.00389	0.0245	*AKT3, PIK3CD, PIK3R5, PAK4, PTK2*
Glioma Signaling[b]	0.00407	0.0251	*AKT3, PIK3CD, CDKN1A, PIK3R5, CDK6, PRKCZ*
IL-15 Signaling	0.00417	0.0251	*AKT3, PIK3CD, TNF, PIK3R5, PTK2*
Colorectal Cancer Metastasis Signaling	0.00427	0.0251	*LRP5, AKT3, PIK3CD, MMP9, TNF, PIK3R5, GNG12, PRKAR1B, ADCY9, ARRB1*
Nitric Oxide Signaling in the Cardiovascular System	0.00427	0.0251	*AKT3, PIK3CD, PIK3R5, PRKAR1B, PRKCZ, CACNA1D*
Erythropoietin Signaling	0.00437	0.0251	*AKT3, PIK3CD, PIK3R5, PTPN6, PRKCZ*
GNRH Signaling	0.00447	0.0251	*ATF4, PRKAR1B, EGR1, PAK4, PTK2, PRKCZ, ADCY9*
Melanoma Signaling[b]	0.00447	0.0251	*AKT3, PIK3CD, CDKN1A, PIK3R5*
Insulin Receptor Signaling	0.00457	0.0257	*AKT3, PIK3CD, RPTOR, STXBP4, PIK3R5, PRKAR1B, PRKCZ*
IGF-1 Signaling	0.00468	0.0257	*AKT3, PIK3CD, PIK3R5, PRKAR1B, PTK2, PRKCZ*
Growth Hormone Signaling	0.00501	0.0263	*PIK3CD, RPS6KA2, PIK3R5, PTPN6, PRKCZ*
Renal Cell Carcinoma Signaling[b]	0.00501	0.0263	*AKT3, PIK3CD, PIK3R5, ETS1, PAK4*
Ovarian Cancer Signaling	0.00501	0.0263	*AKT3, PIK3CD, MMP9, PIK3R5, PRKAR1B, BRCA2, ARRB1*
FLT3 Signaling in Hematopoietic Progenitor Cells	0.00562	0.0288	*ATF4, AKT3, PIK3CD, RPS6KA2, PIK3R5*
Role of Oct4 in Mammalian Embryonic Stem Cell Pluripotency	0.00575	0.0295	*RARA, RXRB, NR2F6, ETS2*
JAK/Stat Signaling	0.00603	0.0302	*AKT3, PIK3CD, CDKN1A, PIK3R5, PTPN6*
Leptin Signaling in Obesity	0.00631	0.0316	*AKT3, PIK3CD, PIK3R5, PRKAR1B, ADCY9*
Phosphatidylinositol signaling system[c]	0.00768	0.0643	*DGKA, ITPK1, PIK3CD, PIK3R5, PIP4K2A, PIP5K1C*
Natural Killer Cell Signaling	0.00794	0.0372	*AKT3, PIK3CD, PIK3R5, PTPN6, PAK4, PRKCZ*
Sphingosine-1-phosphate Signaling	0.00794	0.0372	*AKT3, PIK3CD, GNA12, PIK3R5, PTK2, ADCY9*
VDR/RXR Activation	0.00794	0.0372	*NCOR2, LRP5, CDKN1A, RXRB, PRKCZ*
Regulation of eIF4 and p70S6K Signaling	0.00813	0.0372	*AKT3, PIK3CD, RPS18, PIK3R5, AGO2, PRKCZ, EIF4G1*
Fc Epsilon RI Signaling[b]	0.00832	0.0380	*IL3, AKT3, PIK3CD, TNF, PIK3R5, PRKCZ*
IL-15 Production	0.00891	0.0398	*PTK6, PTK2, PRKCZ*
Prostate Cancer Signaling[b]	0.00933	0.0407	*ATF4, AKT3, PIK3CD, CDKN1A, PIK3R5*
Semaphorin Signaling in Neurons	0.00955	0.0417	*SEMA7A, PAK4, FES, PTK2*
Clathrin-mediated Endocytosis Signaling	0.00977	0.0427	*LDLR, PIK3CD, ACTB, AP2A2, PIK3R5, PIP5K1C, FGF23, ARRB1*
T Cell Teceptor Signaling Pathway[c]	0.0102	0.0675	*AKT3, NFATC1, PAK4, PIK3CD, PIK3R5, PTPN6, TNF*
Lymphotoxin β Receptor Signaling	0.0110	0.0468	*AKT3, PIK3CD, PIK3R5, LTB*
Insulin Signaling Pathway[c]	0.0113	0.0675	*ACACA, AKT3, PIK3CD, PIK3R5, PRKAR1B, PRKCZ, RPTOR, SORBS1*
Gα12/13 Signaling	0.0115	0.0479	*AKT3, PIK3CD, GNA12, PIK3R5, PTK2, F2RL3*
Breast Cancer Regulation by Stathmin1	0.0117	0.0490	*ARHGEF3, PIK3CD, CDKN1A, PIK3R5, GNG12, PRKAR1B, PRKCZ, ADCY9*

α-Adrenergic Signaling	0.0117	0.0490	*GNG12, PRKAR1B, ADRA2A, PRKCZ, ADCY9*

Note:

[a]Pathways were identified by the software of IPA (FDR Q-value<0.05).

[b]Pathways were identified by the software of IPA, EnrichNet and Genetrail (FDR Q-value<0.05).

[c]Pathways were identified by the software of EnrichNet and Genetrail (FDR Q<0.05).

[d]The number of enriched genes ≥ 3 in each term was included.

Supplemental Table S5: The putative pathways associated with smoking-attributed cancer from blood samples[a]

Canonical Pathways	P-value	FDR	Genes Included[d]	References[e]
MSP-RON Signaling Pathway	6.17×10^{-07}	0.00022	*ITGAM, IL3, PIK3CD, ACTB, RPS6KA2, TNF, PIK3R5, PRKCZ*	[36]
RAR Activation	2.04×10^{-06}	0.00037	*NCOR2, AKT3, RARA, ZBTB16, SMAD6, RXRB, SMARCA4, NR2F6, PIK3CD, ACTB, PRKAR1B, RARG, PRKCZ, ADCY9*	[37]
Rac Signaling	6.17×10^{-06}	0.00071	*BAIAP2, PIK3CD, PARD3, PIK3R5, MCF2L, PIP5K1C, PAK4, PTK2, PRKCZ, PIP4K2A*	[38]
Actin Cytoskeleton Signaling[b]	7.94×10^{-06}	0.00071	*GNA12, PIK3R5, MYH10, FGF23, PTK2, PIP4K2A, BAIAP2, PIK3CD, ACTB, TRIO, GNG12, PIP5K1C, PAK4, TIAM2*	[39]
Aryl Hydrocarbon Receptor Signaling	1.15×10^{-05}	0.00083	*NCOR2, AHRR, RARA, TNF, CYP1A1, CDKN1A, RXRB, SMARCA4, CDK6, RARG, NFE2L2*	[40, 41]
Signaling by Rho Family GTPases	2.51×10^{-05}	0.00151	*ARHGEF3, PARD3, GNA12, PIK3R5, PTK2, PIP4K2A, BAIAP2, PIK3CD, ACTB, SEPT9, GNG12, PIP5K1C, PAK4, PRKCZ*	[42]
AMPK Signaling	2.95×10^{-05}	0.00155	*CRTC2, ATF4, AKT3, PIK3CD, RPTOR, ACTB, CDKN1A, PIK3R5, PRKAR1B, SMARCA4, ADRA2A, ACACA*	[43]
Renin-Angiotensin Signaling	6.03×10^{-05}	0.00275	*PIK3CD, TNF, PIK3R5, PRKAR1B, PTPN6, PAK4, PTK2, PRKCZ, ADCY9*	[44]
Molecular Mechanisms of Cancer[b]	7.41×10^{-05}	0.00295	*LRP5, ARHGEF3, AKT3, NOTCH1, GNA12, SMAD6, PIK3R5, PTK2, PIK3CD, PSEN2, CDKN1A, PRKAR1B, PAK4, CDK6, PRKCZ, PMAIP1, ADCY9*	[45]
CXCR4 Signaling	0.000166	0.00575	*AKT3, PIK3CD, GNA12, PIK3R5, GNG12, EGR1, PAK4, PTK2, PRKCZ, ADCY9*	[46]
ERK/MAPK Signaling	0.000214	0.00575	*KSR1, ATF4, PIK3CD, PIK3R5, PRKAR1B, ETS1, PAK4, PTK2, NFATC1, DUSP4, ETS2*	[47]
HER-2 Signaling in Breast Cancer	0.000214	0.00575	*AKT3, PIK3CD, PARD3, CDKN1A, PIK3R5, CDK6, PRKCZ*	[48]
Thrombin Signaling	0.000224	0.00575	*GATA3, ARHGEF3, AKT3, PIK3CD, GNA12, PIK3R5, GNG12, PTK2, F2RL3, PRKCZ, ADCY9*	[49]

HGF Signaling	0.000269	0.00603	*AKT3, PIK3CD, CDKN1A, PIK3R5, ETS1, PTK2, PRKCZ, ETS2*	[50]
Relaxin Signaling	0.000282	0.00603	*AKT3, PIK3CD, MMP9, GNA12, PIK3R5, GNG12, PRKAR1B, PRKCZ, ADCY9*	[51, 52]
Role of Tissue Factor in Cancer	0.000331	0.00631	*AKT3, PIK3CD, RPS6KA2, GNA12, PIK3R5, FGR, EGR1, ARRB1*	[53, 54]
Non-Small Cell Lung Cancer Signaling[b]	0.000603	0.00955	*AKT3, PIK3CD, PIK3R5, RXRB, CDK6, RASSF5*	[55, 56]
G-Protein Coupled Receptor Signaling	0.000851	0.0120	*ATF4, AKT3, PIK3CD, PIK3R5, HRH1, PRKAR1B, ADRA2A, CNR2, AVPR1B, RGS12, ADCY9, DUSP4*	[49]
Telomerase Signaling	0.000891	0.0120	*AKT3, PIK3CD, CDKN1A, PIK3R5, ETS1, TERT, ETS2*	[57, 58]
Small Cell Lung Cancer Signaling[b]	0.000977	0.0120	*AKT3, PIK3CD, PIK3R5, RXRB, CDK6, PTK2*	[59, 60]
Hereditary Breast Cancer Signaling	0.000977	0.0120	*AKT3, PIK3CD, ACTB, CDKN1A, PIK3R5, BRCA2, SMARCA4, CDK6*	[61]
Chemokine signaling pathway	0.000978	0.0185	*ADCY9, AKT3, ARRB1, CXCR5, FGR, GNG12, PARD3, PIK3CD, PIK3R5, PRKCZ, PTK2, TIAM2*	[62]
NF-κB Activation by Viruses	0.00112	0.0126	*ITGAL, AKT3, PIK3CD, CXCR5, PIK3R5, PRKCZ*	[63]
Pancreatic Adenocarcinoma Signaling[b]	0.00158	0.0170	*AKT3, PIK3CD, NOTCH1, MMP9, CDKN1A, PIK3R5, BRCA2*	[64]
NGF Signaling	0.00158	0.0170	*ATF4, AKT3, PIK3CD, RPS6KA2, TRIO, PIK3R5, PRKCZ*	[65, 66]
Ceramide Signaling	0.00182	0.0174	*KSR1, AKT3, PIK3CD, TNF, PIK3R5, PRKCZ*	[67]
ErbB4 Signaling	0.00234	0.0204	*PIK3CD, PSEN2, PIK3R5, YAP1, PRKCZ*	[68]
FAK Signaling	0.00257	0.0204	*AKT3, PIK3CD, ACTB, PIK3R5, PAK4, PTK2*	[69]
ILK Signaling	0.00263	0.0204	*ATF4, AKT3, PIK3CD, MMP9, ACTB, TNF, PIK3R5, MYH10, PTK2*	[70]
14-3-3-mediated Signaling	0.00269	0.0204	*AKT3, PIK3CD, TNF, PIK3R5, YAP1, EDC3, PRKCZ*	[71]
Antiproliferative Role of Somatostatin Receptor 2	0.00275	0.0204	*PIK3CD, CDKN1A, PIK3R5, GNG12, PTPN6*	[72]
mTOR Signaling[b]	0.00275	0.0204	*AKT3, PIK3CD, RPTOR, RPS18, RPS6KA2, DDIT4, PIK3R5, PRKCZ, EIF4G1*	[73]
Regulation of the Epithelial-Mesenchymal Transition Pathway	0.00275	0.0204	*AKT3, PIK3CD, NOTCH1, MMP9, PSEN2, PIK3R5, EGR1, ETS1, FGF23*	[74]
IL-8 Signaling	0.00282	0.0209	*ITGAM, AKT3, PIK3CD, MMP9, GNA12, PIK3R5, GNG12, PTK2, PRKCZ*	[75, 76]
PTEN Signaling	0.00295	0.0209	*AKT3, PIK3CD, TGFBR3, CDKN1A, PIK3R5, PTK2, PRKCZ*	[77, 78]

Gap Junction Signaling	0.00309	0.0209	*AKT3, PIK3CD, ACTB, CSNK1G3, PIK3R5, PRKAR1B, PRKCZ, ADCY9*	[79, 80]
VEGF Signaling[b]	0.00309	0.0209	*AKT3, PIK3CD, ACTB, PIK3R5, PTPN6, PTK2*	[81]
RhoA Signaling	0.00324	0.0219	*BAIAP2, ACTB, GNA12, SEPT9, PIP5K1C, PTK2, PIP4K2A*	[42]
Estrogen-Dependent Breast Cancer Signaling	0.00339	0.0224	*ATF4, AKT3, PIK3CD, PIK3R5, TERT*	[82, 83]
Axonal Guidance Signaling	0.00380	0.0245	*AKT3, MMP9, GNA12, PIK3R5, NFATC1, PTK2, RASSF5, BAIAP2, PIK3CD, SEMA7A, GNG12, PRKAR1B, PAK4, FES, PRKCZ*	[84, 85]
Angiopoietin Signaling	0.00389	0.0245	*AKT3, PIK3CD, PIK3R5, PAK4, PTK2*	[86]
Glioma Signaling[b]	0.00407	0.0251	*AKT3, PIK3CD, CDKN1A, PIK3R5, CDK6, PRKCZ*	[87]
IL-15 Signaling	0.00417	0.0251	*AKT3, PIK3CD, TNF, PIK3R5, PTK2*	[88]
Colorectal Cancer Metastasis Signaling	0.00427	0.0251	*LRP5, AKT3, PIK3CD, MMP9, TNF, PIK3R5, GNG12, PRKAR1B, ADCY9, ARRB1*	[89, 90]
Erythropoietin Signaling	0.00437	0.0251	*AKT3, PIK3CD, PIK3R5, PTPN6, PRKCZ*	[91, 92]
GNRH Signaling	0.00447	0.0251	*ATF4, PRKAR1B, EGR1, PAK4, PTK2, PRKCZ, ADCY9*	[93, 94]
Melanoma Signaling[b]	0.00447	0.0251	*AKT3, PIK3CD, CDKN1A, PIK3R5*	[95, 96]
Insulin Receptor Signaling	0.00457	0.0257	*AKT3, PIK3CD, RPTOR, STXBP4, PIK3R5, PRKAR1B, PRKCZ*	[97]
IGF-1 Signaling	0.00468	0.0257	*AKT3, PIK3CD, PIK3R5, PRKAR1B, PTK2, PRKCZ*	[98]
Renal Cell Carcinoma Signaling[b]	0.00501	0.0263	*AKT3, PIK3CD, PIK3R5, ETS1, PAK4*	[99]
Ovarian Cancer Signaling	0.00501	0.0263	*AKT3, PIK3CD, MMP9, PIK3R5, PRKAR1B, BRCA2, ARRB1*	[100]
JAK/Stat Signaling	0.00603	0.0302	*AKT3, PIK3CD, CDKN1A, PIK3R5, PTPN6*	[101]
Natural Killer Cell Signaling	0.00794	0.0372	*AKT3, PIK3CD, PIK3R5, PTPN6, PAK4, PRKCZ*	[102, 103]
Sphingosine-1-phosphate Signaling	0.00794	0.0372	*AKT3, PIK3CD, GNA12, PIK3R5, PTK2, ADCY9*	[104]
Prostate Cancer Signaling[b]	0.00933	0.0407	*ATF4, AKT3, PIK3CD, CDKN1A, PIK3R5*	[105]
Lymphotoxin β Receptor Signaling	0.01096	0.04678	*AKT3, PIK3CD, PIK3R5, LTB*	[106]

Note:

[a]Pathways were identified by the software of IPA (FDR Q-value<0.05).

[b]Pathways were identified by the software of IPA, EnrichNet and Genetrail (FDR Q-value<0.05).

[c]Pathways were identified by the software of EnrichNet and Genetrail (FDR Q<0.05).

[d]The number of enriched genes ≥ 3 in each term was included.

[e]References: previously reported studies in supporting of the detected pathways associated with cnacers.

Supplemental Table S6: All common pathways overrepresented by SA-DNAm-related genes from the two different biomaterials (p-value<0.05)

Canonical Pathways	In Blood Samples			In Buccal Samples	
	No. of Genes	P-value	FDR	No. of Genes	P-value
RAR Activation	14	2.04×10^{-06}	0.00037	13	0.0076
Actin Cytoskeleton Signaling	14	7.94×10^{-06}	0.00071	13	0.0186
Aryl Hydrocarbon Receptor Signaling	11	1.15×10^{-05}	0.00083	11	0.0043
Signaling by Rho Family GTPases	14	2.51×10^{-05}	0.00151	13	0.0389
Molecular Mechanisms of Cancer	17	7.41×10^{-05}	0.00295	28	1.55×10^{-05}
Thrombin Signaling	11	0.00022	0.00575	14	0.0030
Leukocyte Extravasation Signaling	11	0.00030	0.00603	13	0.0098
P2Y Purigenic Receptor Signaling Pathway	8	0.00063	0.00955	8	0.0363
G-Protein Coupled Receptor Signaling	12	0.00085	0.0120	17	0.0036
CREB Signaling in Neurons	9	0.00166	0.0170	10	0.0468
Reelin Signaling in Neurons	6	0.00170	0.0170	6	0.0417
Epithelial Adherens Junction Signaling	8	0.00219	0.0204	10	0.0174
PTEN Signaling	7	0.00295	0.0209	9	0.0138
Virus Entry via Endocytic Pathways	6	0.00309	0.0209	7	0.0245
Axonal Guidance Signaling	15	0.00380	0.0245	22	0.0200
Colorectal Cancer Metastasis Signaling	10	0.00427	0.0251	13	0.0363
GNRH Signaling	7	0.00447	0.0251	9	0.0214
VDR/RXR Activation	5	0.00794	0.0372	7	0.0117
Breast Cancer Regulation by Stathmin1	8	0.0117	0.0490	12	0.0200
α-Adrenergic Signaling	5	0.0117	0.0490	7	0.0195
Gαi Signaling	6	0.0129	0.0501	8	0.0398
Cellular Effects of Sildenafil (Viagra)	6	0.0148	0.0537	8	0.0468
L-9 Signaling	3	0.0170	0.0589	4	0.0229
GM-CSF Signaling	4	0.0174	0.0603	5	0.0490
Tight Junction Signaling	7	0.0178	0.0603	11	0.0145
RhoGDI Signaling	7	0.0214	0.0676	11	0.0234
Notch Signaling Pathway	3	0.0214	0.0676	8	0.0004
Sertoli Cell-Sertoli Cell Junction	7	0.0219	0.0676	12	0.0102

ignaling					
enobiotic Metabolism Signaling	9	0.0229	0.0692	21	8.51×10^{-05}
ole of NFAT in Cardiac Hypertrophy	7	0.0234	0.0708	16	0.00018
dherens junction	5	0.0257	0.0910	9	0.0023
PCR-Mediated Nutrient Sensing n Enteroendocrine Cells	4	0.0447	0.1072	7	0.0174

Supplemental Table S7: Molecular functions of SA-DNAm-enriched genes from blood samples by Gene Ontology (GO) analysis.

GO-ID	Molecular Functions	No. of Genes [a]	Enrichment	P-value	FDR
GO:0016563	transcription activator activity	24	3.22	4.92×10^{-07}	0.00019
GO:0043565	sequence-specific DNA binding	30	2.74	6.20×10^{-07}	0.00019
GO:0005515	protein binding	178	1.26	2.63×10^{-06}	0.00054
GO:0008134	transcription factor binding	24	2.65	1.45×10^{-05}	0.0022
GO:0004035	alkaline phosphatase activity	3	43.17	2.05×10^{-05}	0.0025
GO:0030528	transcription regulator activity	48	1.83	2.54×10^{-05}	0.0026
GO:0005488	binding	239	1.11	4.26×10^{-05}	0.0038
GO:0003708	retinoic acid receptor activity	3	28.78	9.98×10^{-05}	0.0069
GO:0005524	ATP binding	45	1.76	1.23×10^{-04}	0.0073
GO:0005548	phospholipid transporter activity	5	9.92	1.29×10^{-04}	0.0073
GO:0019904	protein domain specific binding	17	2.52	4.60×10^{-04}	0.017
GO:0003779	actin binding	15	2.67	5.44×10^{-04}	0.019
GO:0003712	transcription cofactor activity	16	2.54	6.32×10^{-04}	0.020
GO:0003676	nucleic acid binding	79	1.40	6.54×10^{-04}	0.020
GO:0003700	transcription factor activity	30	1.82	1.12×10^{-03}	0.032
GO:0008092	cytoskeletal protein binding	19	2.16	1.42×10^{-03}	0.038
GO:0019899	enzyme binding	22	2.00	1.61×10^{-03}	0.038
GO:0042162	telomeric DNA binding	3	12.33	1.64×10^{-03}	0.038
GO:0003677	DNA binding	58	1.43	2.43×10^{-03}	0.049

Note: [a] The number of enriched genes ≥ 3 in each term was included.

Supplemental Table S8: Significantly negative correlation between methylation and RNA expression in lung adenocarcinoma (LUAD)

CpG Loci	Chromosome & Position	Gene Region	Gene Name	LUAD	
				Correlation Coefficient (r)[a]	P-value
cg07151117	chr8:29204954	5'UTR, gene body	*DUSP4*	r=-0.742	**p<0.001**
cg24379915	chr8:29202958	gene body	*DUSP4*	r=-0.657	**p<0.001**
cg27514333	chr15:66996626	gene body	*SMAD6*	r=-0.422	**p<0.001**

cg04265051	chr11:68079686	TSS+/-1500	*LRP5*	r=-0.396	**p<0.001**
cg04813697	chr10:22920025	gene body	*PIP4K2A*	r=-0.395	**p<0.001**
cg19572487	chr17:38476024	5'UTR	*RARA*	r=-0.394	**p<0.001**
cg08052292	chr3:56789178	gene body	*ARHGEF3*	r=-0.377	**p<0.001**
cg26271591	chr2:178125956	5'UTR, gene body	*NFE2L2*	r=-0.352	**p<0.001**
cg13937905	chr12:53612551	gene body	*RARG*	r=-0.332	**p<0.001**
cg10179300	chr5:14147618	gene body	*TRIO*	r=-0.305	**p<0.001**
cg10062919	chr17:38503802	gene body	*RARA*	r=-0.295	**p<0.001**
cg10592478	chr12:53612641	gene body	*RARG*	r=-0.292	**p<0.001**
cg13399816	chr1:68299468	TSS+/-1500	*GNG12*	r=-0.289	**p<0.001**
cg23098018	chr1:9775755	gene body	*PIK3CD*	r=-0.288	**p<0.001**
cg26654286	chr17:38465510	5'UTR, 1st exon	*RARA*	r=-0.281	**p<0.001**
cg12406027	chr19:39616818	5'UTR	*PAK4*	r=-0.274	**p<0.001**
cg07793148	chr1:9712988	5'UTR	*PIK3CD*	r=-0.271	**p<0.001**
cg13184736	chr1:68299409	TSS+/-1500	*GNG12*	r=-0.268	**p<0.001**
cg01901332	chr11:75031054	gene body	*ARRB1*	r=-0.262	**p<0.001**
cg18405341	chr22:39918625	3'UTR	*ATF4*	r=-0.260	**p<0.001**
cg01207684	chr16:4103167	gene body	*ADCY9*	r=-0.257	**p<0.001**
cg03140521	chr1:68299388	TSS+/-1500	*GNG12*	r=-0.257	**p<0.001**
cg06223834	chr16:4103161	gene body	*ADCY9*	r=-0.254	**p<0.001**
cg26764244	chr1:68299511	TSS+/-1500	*GNG12*	r=-0.245	**p<0.001**
cg05824218	chr17:38499096	1st exon, gene body	*RARA*	r=-0.237	**p<0.001**
cg09637172	chr6:31545242	gene body	*TNF*	r=-0.234	**p<0.001**
cg25189904	chr1:68299493	TSS+/-1500	*GNG12*	r=-0.230	**p<0.001**
cg09858022	chr17:38465333	TSS+/-200	*RARA*	r=-0.226	**p<0.001**
cg05329352	chr10:112838983	1st exon	*ADRA2A*	r=-0.203	**p<0.001**
cg03604424	chr5:14171590	gene body	*TRIO*	r=-0.198	**p<0.001**
cg16276850	chr17:38498914	1st exon, 5'UTR, gene body	*RARA*	r=-0.190	**p<0.001**
cg14817490	chr5:392920	gene body	*AHRR*	r=-0.185	**p<0.001**
cg11554391	chr5:321320	gene body	*AHRR*	r=-0.181	**p<0.001**
cg15059065	chr19:17354961	gene body	*NR2F6*	r=-0.171	**p<0.001**
cg03725573	chr11:113962901	gene body	*ZBTB16*	r=-0.166	**p<0.001**
cg08553327	chr6:31543647	1st exon	*TNF*	r=-0.150	**p<0.01**
cg26729380	chr6:31543655	1st exon	*TNF*	r=-0.142	**p<0.01**
cg06710464	chr17:79047695	gene body	*BAIAP2*	r=-0.133	**p<0.01**
cg11884933	chr7:2774414	gene body	*GNA12*	r=-0.123	**p<0.05**
cg25799109	chr3:57102900	5'UTR	*ARHGEF3*	r=-0.115	**p<0.05**
cg25677394	chr3:11178758	TSS+/-200	*HRH1*	r=-0.115	**p<0.05**
cg24090911	chr5:400732	gene body	*AHRR*	r=-0.114	**p<0.05**
cg12661610	chr4:3412653	gene body	*RGS12*	r=-0.109	**p<0.05**

Note: [a] indicates the Pearson correlation coefficient calculated by the web-based tool of MEXPRESS.

Supplemental Table S9: Significantly positive correlation between methylation and RNA expression in lung adenocarcinoma (LUAD)

CpG Loci	Chromosome & Position	Gene Region	Gene Name	LUAD	
				Correlation Coefficient (r)[a]	P-value
cg24538512	chr18:77233465	gene body	*NFATC1*	r=+0.503	**p<0.001**
cg05944967	chr18:77166811	5'UTR, gene body	*NFATC1*	r=+0.459	**p<0.001**
cg02385153	chr5:404766	gene body	*AHRR*	r=+0.442	**p<0.001**
cg11314684	chr1:244006288	gene body	*AKT3*	r=+0.404	**p<0.001**
cg10841124	chr5:433274	gene body	*AHRR*	r=+0.367	**p<0.001**
cg11902777	chr5:368843	gene body	*AHRR*	r=+0.364	**p<0.001**
cg07805542	chr1:9779309	gene body	*PIK3CD*	r=+0.353	**p<0.001**
cg06688763	chr7:92238207	3'UTR	*CDK6*	r=+0.337	**p<0.001**
cg26850624	chr5:429559	gene body	*AHRR*	r=+0.335	**p<0.001**
cg10251229	chr7:630581	gene body	*PRKAR1B*	r=+0.321	**p<0.001**
cg01899089	chr5:369969	gene body	*AHRR*	r=+0.309	**p<0.001**
cg13256912	chr3:11211081	5'UTR	*HRH1*	r=+0.300	**p<0.001**
cg07413467	chr7:92238086	3'UTR	*CDK6*	r=+0.297	**p<0.001**
cg15261712	chr7:92238248	3'UTR	*CDK6*	r=+0.292	**p<0.001**
cg04226002	chr11:113953462	gene body	*ZBTB16*	r=+0.292	**p<0.001**
cg03120555	chr7:630473	gene body	*PRKAR1B*	r=+0.285	**p<0.001**
cg17518710	chr11:113954170	gene body	*ZBTB16*	r=+0.264	**p<0.001**
cg17593625	chr7:752800	TSS+/-, 5'UTR, 1st exon	*PRKAR1B*	r=+0.253	**p<0.001**
cg01097768	chr5:378854	gene body	*AHRR*	r=+0.249	**p<0.001**
cg00541718	chr7:630823	gene body	*PRKAR1B*	r=+0.244	**p<0.001**
cg03604011	chr5:400201	gene body	*AHRR*	r=+0.233	**p<0.01**
cg24688690	chr5:345850	gene body	*AHRR*	r=+0.230	**p<0.001**
cg14835981	chr7:752715	TSS+/-200, 5'UTR, TSS+/-1500	*PRKAR1B*	r=+0.227	**p<0.001**
cg02322048	chr19:3646574	gene body	*PIP5K1C*	r=+0.200	**p<0.001**
cg10827488	chr11:113953838	gene body	*ZBTB16*	r=+0.183	**p<0.001**
cg09084391	chr5:346247	gene body	*AHRR*	r=+0.181	**p<0.001**
cg06784563	chr18:77284509	gene body	*NFATC1*	r=+0.168	**p<0.001**
cg09180820	chr1:2113911	gene body	*PRKCZ*	r=+0.164	**p<0.001**
cg26703534	chr5:377358	gene body	*AHRR*	r=+0.148	**p<0.01**
cg23916896	chr5:368804	gene body	*AHRR*	r=+0.147	**p<0.01**
cg26529655	chr5:424371	gene body	*AHRR*	r=+0.130	**p<0.01**

cg16324409	chr4:3416370	gene body	*RGS12*	r=+0.130	**p<0.05**
cg04956244	chr17:38511592	gene body	*RARA*	r=+0.116	**p<0.05**
cg10505873	chr20:44637511	TSS+/-200	*MMP9*	r=+0.114	**p<0.05**
cg05575921	chr5:373378	gene body	*AHRR*	r=+0.110	**p<0.05**

Note: [a] indicates the Pearson correlation coefficient calculated by the web-based tool of MEXPRESS.

Supplemental Table S10: Significantly negative correlation between methylation and RNA expression in lung squamous cell carcinoma (LUSC)

CpG Loci	Chromosome & Position	Gene Region	Gene Name	LUSC	
				Correlation Coefficient (r)[a]	P-value (p)
cg26271591	chr2:178125956	5'UTR, gene body	*NFE2L2*	r=-0.544	**p<0.001**
cg07151117	chr8:29204954	5'UTR, gene body	*DUSP4*	r=-0.485	**p<0.001**
cg27514333	chr15:66996626	gene body	*SMAD6*	r=-0.460	**p<0.001**
cg19572487	chr17:38476024	5'UTR	*RARA*	r=-0.407	**p<0.001**
cg10062919	chr17:38503802	gene body	*RARA*	r=-0.407	**p<0.001**
cg08052292	chr3:56789178	gene body	*ARHGEF3*	r=-0.365	**p<0.001**
cg05329352	chr10:112838983	1st exon	*ADRA2A*	r=-0.349	**p<0.001**
cg12406027	chr19:39616818	5'UTR	*PAK4*	r=-0.341	**p<0.001**
cg04265051	chr11:68079686	TSS+/-1500	*LRP5*	r=-0.335	**p<0.001**
cg10179300	chr5:14147618	gene body	*TRIO*	r=-0.328	**p<0.001**
cg04813697	chr10:22920025	gene body	*PIP4K2A*	r=-0.312	**p<0.001**
cg07793148	chr1:9712988	5'UTR	*PIK3CD*	r=-0.304	**p<0.001**
cg03604424	chr5:14171590	gene body	*TRIO*	r=-0.285	**p<0.001**
cg08553327	chr6:31543647	1st exon	*TNF*	r=-0.282	**p<0.001**
cg13009654	chr5:137802252	gene body	*EGR1*	r=-0.266	**p<0.001**
cg26654286	chr17:38465510	5'UTR, 1st exon	*RARA*	r=-0.263	**p<0.001**
cg26729380	chr6:31543655	1st exon	*TNF*	r=-0.253	**p<0.001**
cg01741041	chr7:5567862	gene body	*ACTB*	r=-0.247	**p<0.001**
cg09858022	chr17:38465333	TSS+/-200	*RARA*	r=-0.247	**p<0.001**
cg05971148	chr7:5568411	gene body	*ACTB*	r=-0.242	**p<0.001**
cg09637172	chr6:31545242	gene body	*TNF*	r=-0.233	**p<0.001**
cg26878655	chr17:38481877	5'UTR	*RARA*	r=-0.210	**p<0.001**
cg11554391	chr5:321320	gene body	*AHRR*	r=-0.202	**p<0.001**
cg15059065	chr19:17354961	gene body	*NR2F6*	r=-0.188	**P<0.01**
cg05824218	chr17:38499096	1st exon, gene body	*RARA*	r=-0.160	**p<0.01**
cg19717773	chr7:2847554	gene body	*GNA12*	r=-0.155	**p<0.05**
cg10592478	chr12:53612641	gene body	*RARG*	r=-0.155	**p<0.05**
cg25212025	chr10:34602937	gene body	*PARD3*	r=-0.154	**p<0.01**
cg23098018	chr1:9775755	gene body	*PIK3CD*	r=-0.154	**p<0.01**

cg18040892	chr19:11144017	gene body	*SMARCA4*	r=-0.153	**p<0.05**
cg14554244	chr9:139393956	gene body	*NOTCH1*	r=-0.142	**p<0.05**
cg18446336	chr7:2847575	gene body	*GNA12*	r=-0.140	**p<0.05**
cg23963476	chr19:11099907	gene body	*SMARCA4*	r=-0.139	**p<0.05**
cg04551776	chr5:393366	gene body	*AHRR*	r=-0.137	**p<0.05**
cg13937905	chr12:53612551	gene body	*RARG*	r=-0.136	**p<0.05**
cg26764244	chr1:68299511	TSS+/-1500	*GNG12*	r=-0.123	**p<0.05**

Note: [a] indicates the Pearson correlation coefficient calculated by the web-based tool of MEXPRESS.

Supplemental Table S11: Significantly positive correlation between methylation and RNA expression in lung squamous cell carcinoma (LUSC)

CpG Loci	Chromosome & Position	Gene Region	Gene Name	LUSC	
				Correlation Coefficient (r)[a]	P-value (p)
cg11314684	chr1:244006288	gene body	*AKT3*	r=+0.422	**p<0.001**
cg03604011	chr5:400201	gene body	*AHRR*	r=+0.334	**p<0.001**
cg11902777	chr5:368843	gene body	*AHRR*	r=+0.324	**p<0.001**
cg26850624	chr5:429559	gene body	*AHRR*	r=+0.323	**p<0.001**
cg07805542	chr1:9779309	gene body	*PIK3CD*	r=+0.311	**p<0.001**
cg02385153	chr5:404766	gene body	*AHRR*	r=+0.307	**p<0.001**
cg23916896	chr5:368804	gene body	*AHRR*	r=+0.304	**p<0.001**
cg07967717	chr1:24229682	5'UTR	*CNR2*	r=+0.293	**p<0.001**
cg26404511	chr1:24229575	5'UTR	*CNR2*	r=+0.286	**p<0.001**
cg26529655	chr5:424371	gene body	*AHRR*	r=+0.285	**p<0.001**
cg07413467	chr7:92238086	3'UTR	*CDK6*	r=+0.269	**p<0.001**
cg13256912	chr3:11211081	5'UTR	*HRH1*	r=+0.245	**p<0.001**
cg10827488	chr11:113953838	gene body	*ZBTB16*	r=+0.244	**p<0.001**
cg05460226	chr17:8804279	gene body	*PIK3R5*	r=+0.243	**p<0.001**
cg05944967	chr18:77166811	5'UTR, gene body	*NFATC1*	r=+0.240	**p<0.01**
cg24538512	chr18:77233465	gene body	*NFATC1*	r=+0.221	**p<0.001**
cg01899089	chr5:369969	gene body	*AHRR*	r=+0.220	**p<0.001**
cg06688763	chr7:92238207	3'UTR	*CDK6*	r=+0.220	**p<0.001**
cg10841124	chr5:433274	gene body	*AHRR*	r=+0.218	**p<0.001**
cg03120555	chr7:630473	gene body	*PRKAR1B*	r=+0.215	**p<0.001**
cg00541718	chr7:630823	gene body	*PRKAR1B*	r=+0.202	**p<0.001**
cg03991871	chr5:368447	gene body	*AHRR*	r=+0.199	**p<0.001**
cg03611151	chr1:24229581	5'UTR	*CNR2*	r=+0.193	**p<0.01**
cg14835981	chr7:752715	TSS+/-200, 5'UTR, TSS+/-1500	*PRKAR1B*	r=+0.192	**p<0.001**

cg00931843	chr6:155442993	5'UTR	*TIAM2*	r=+0.191	**p<0.01**
cg19276111	chr1:24229232	5'UTR	*CNR2*	r=+0.189	**p<0.01**
cg15261712	chr7:92238248	3'UTR	*CDK6*	r=+0.186	**p<0.01**
cg10251229	chr7:630581	gene body	*PRKAR1B*	r=+0.184	**p<0.001**
cg14120703	chr9:139416102	gene body	*NOTCH1*	r=+0.160	**p<0.01**
cg17518710	chr11:113954170	gene body	*ZBTB16*	r=+0.158	**p<0.01**
cg26703534	chr5:377358	gene body	*AHRR*	r=+0.156	**p<0.01**
cg17593625	chr7:752800	TSS+/-, 5'UTR, 1st exon	*PRKAR1B*	r=+0.152	**p<0.01**
cg17924476	chr5:323794	gene body	*AHRR*	r=+0.136	**p<0.05**
cg04226002	chr11:113953462	gene body	*ZBTB16*	r=+0.135	**p<0.05**
cg24379915	chr8:29202958	gene body	*DUSP4*	r=+0.134	**p<0.05**
cg01097768	chr5:378854	gene body	*AHRR*	r=+0.131	**p<0.05**
cg05575921	chr5:373378	gene body	*AHRR*	r=+0.129	**p<0.05**

Note: [a] indicates the Pearson correlation coefficient calculated by the web-based tool of MEXPRESS.

Supplemental Table S12: Summary of methylation loci correlated with RNA expression showed significant differences between control and lung cancer.

	LUAD		LUSC	
	Positive correlation	Negative correlation	Positive correlation	Negative correlation
Total loci	42	35	36	37
Hypermethlated loci (%) [a]	21 (50.0%)	6 (17.1%)	12 (33.3%)	7 (16.2%)
Hypomethlated loci (%) [b]	11 (26.2%)	17 (48.6%)	6 (19.4%)	14 (37.8%)

Note: [a] Signigicantly hypermethylated loci in lung cancer compared to control samples; The significant differences between cancer and control were calculated by using the Wilcoxon's rank-sum test. [b] Significantly hypomethylated loci in lung cancer compared to control samples; The significant differences between cancer and control were calculated by using the Wilcoxon's rank-sum test.

References

1. Xu, Q., et al., *Determination of methylated CpG sites in the promoter region of catechol-O-methyltransferase (COMT) and their involvement in the etiology of tobacco smoking.* Frontiers in psychiatry, 2010. **1**: p. 16.
2. Philibert, R.A., et al., *MAOA methylation is associated with nicotine and alcohol dependence in women.* American Journal of Medical Genetics Part B: Neuropsychiatric Genetics, 2008. **147**(5): p. 565-570.
3. Philibert, R.A., et al., *The effect of smoking on MAOA promoter methylation in DNA prepared from lymphoblasts and whole blood.* American Journal of Medical Genetics Part B: Neuropsychiatric Genetics, 2010. **153**(2): p. 619-628.

4. Breitling, L.P., et al., *Smoking, F2RL3 methylation, and prognosis in stable coronary heart disease.* European heart journal, 2012. **33**(22): p. 2841-2848.
5. Shenker, N.S., et al., *DNA methylation as a long-term biomarker of exposure to tobacco smoke.* Epidemiology, 2013. **24**(5): p. 712-716.
6. Zhang, Y., et al., *F2RL3 methylation in blood DNA is a strong predictor of mortality.* International journal of epidemiology, 2014. **43**(4): p. 1215-1225.
7. Zhang, Y., et al., *F2RL3 methylation as a biomarker of current and lifetime smoking exposures.* Environmental Health Perspectives (Online), 2014. **122**(2): p. 131.
8. Peluso, M.E., et al., *Aberrant methylation of hypermethylated-in-cancer-1 and exocyclic DNA adducts in tobacco smokers.* toxicological sciences, 2014. **137**(1): p. 47-54.
9. Siedlinski, M., et al., *Association of cigarette smoking and CRP levels with DNA methylation in alpha-1 antitrypsin deficiency.* Epigenetics, 2012. **7**(7): p. 720-8.
10. Breitling, L.P., et al., *Tobacco-smoking-related differential DNA methylation: 27K discovery and replication.* Am J Hum Genet, 2011. **88**(4): p. 450-7.
11. Wan, E.S., et al., *Cigarette smoking behaviors and time since quitting are associated with differential DNA methylation across the human genome.* Hum Mol Genet, 2012. **21**(13): p. 3073-82.
12. Sun, Y.V., et al., *Epigenomic association analysis identifies smoking-related DNA methylation sites in African Americans.* Hum Genet, 2013. **132**(9): p. 1027-37.
13. Zeilinger, S., et al., *Tobacco smoking leads to extensive genome-wide changes in DNA methylation.* PLoS One, 2013. **8**(5): p. e63812.
14. Shenker, N.S., et al., *Epigenome-wide association study in the European Prospective Investigation into Cancer and Nutrition (EPIC-Turin) identifies novel genetic loci associated with smoking.* Hum Mol Genet, 2013. **22**(5): p. 843-51.
15. Elliott, H.R., et al., *Differences in smoking associated DNA methylation patterns in South Asians and Europeans.* Clin Epigenetics, 2014. **6**(1): p. 4.
16. Dogan, M.V., et al., *The effect of smoking on DNA methylation of peripheral blood mononuclear cells from African American women.* BMC Genomics, 2014. **15**: p. 151.
17. Harlid, S., et al., *CpG sites associated with cigarette smoking: analysis of epigenome-wide data from the Sister Study.* Environ Health Perspect, 2014. **122**(7): p. 673-8.
18. Tsaprouni, L.G., et al., *Cigarette smoking reduces DNA methylation levels at multiple genomic loci but the effect is partially reversible upon cessation.* Epigenetics, 2014. **9**(10): p. 1382-96.
19. Besingi, W. and A. Johansson, *Smoke-related DNA methylation changes in the etiology of human disease.* Hum Mol Genet, 2014. **23**(9): p. 2290-7.
20. Zaghlool, S.B., et al., *Association of DNA methylation with age, gender, and smoking in an Arab population.* Clin Epigenetics, 2015. **7**(1): p. 6.
21. Allione, A., et al., *Novel epigenetic changes unveiled by monozygotic twins discordant for smoking habits.* PLoS One, 2015. **10**(6): p. e0128265.
22. Guida, F., et al., *Dynamics of smoking-induced genome-wide methylation changes with time since smoking cessation.* Hum Mol Genet, 2015. **24**(8): p. 2349-59.
23. Philibert, R.A., S.R. Beach, and G.H. Brody, *Demethylation of the aryl hydrocarbon receptor repressor as a biomarker for nascent smokers.* Epigenetics, 2012. **7**(11): p. 1331-8.
24. Philibert, R.A., et al., *Changes in DNA methylation at the aryl hydrocarbon receptor repressor may be a new biomarker for smoking.* Clin Epigenetics, 2013. **5**(1): p. 19.

25. Steenaard, R.V., et al., *Tobacco smoking is associated with methylation of genes related to coronary artery disease.* Clin Epigenetics, 2015. **7**(1): p. 54.
26. Zhang, Y., et al., *Smoking-Associated DNA Methylation Biomarkers and Their Predictive Value for All-Cause and Cardiovascular Mortality.* Environ Health Perspect, 2015.
27. Wan, E.S., et al., *Smoking Associated Site Specific Differential Methylation in Buccal Mucosa in the COPDGene Study.* Am J Respir Cell Mol Biol, 2014.
28. Teschendorff, A.E., et al., *Correlation of Smoking-Associated DNA Methylation Changes in Buccal Cells With DNA Methylation Changes in Epithelial Cancer.* JAMA Oncol, 2015. **1**(4): p. 476-85.
29. Buro-Auriemma, L.J., et al., *Cigarette smoking induces small airway epithelial epigenetic changes with corresponding modulation of gene expression.* Hum Mol Genet, 2013. **22**(23): p. 4726-38.
30. Shenker, N.S., et al., *DNA methylation as a long-term biomarker of exposure to tobacco smoke.* Epidemiology, 2013. **24**(5): p. 712-6.
31. Xu, Q., et al., *Determination of Methylated CpG Sites in the Promoter Region of Catechol-O-Methyltransferase (COMT) and their Involvement in the Etiology of Tobacco Smoking.* Front Psychiatry, 2010. **1**: p. 16.
32. Zhang, Y., et al., *F2RL3 methylation in blood DNA is a strong predictor of mortality.* Int J Epidemiol, 2014. **43**(4): p. 1215-25.
33. Peluso, M.E., et al., *Aberrant methylation of hypermethylated-in-cancer-1 and exocyclic DNA adducts in tobacco smokers.* Toxicol Sci, 2014. **137**(1): p. 47-54.
34. Philibert, R.A., et al., *MAOA methylation is associated with nicotine and alcohol dependence in women.* Am J Med Genet B Neuropsychiatr Genet, 2008. **147B**(5): p. 565-70.
35. Philibert, R.A., et al., *The effect of smoking on MAOA promoter methylation in DNA prepared from lymphoblasts and whole blood.* Am J Med Genet B Neuropsychiatr Genet, 2010. **153B**(2): p. 619-28.
36. Yao, H.P., et al., *MSP-RON signalling in cancer: pathogenesis and therapeutic potential.* Nat Rev Cancer, 2013. **13**(7): p. 466-81.
37. Altucci, L., et al., *RAR and RXR modulation in cancer and metabolic disease.* Nat Rev Drug Discov, 2007. **6**(10): p. 793-810.
38. Wertheimer, E., et al., *Rac signaling in breast cancer: a tale of GEFs and GAPs.* Cell Signal, 2012. **24**(2): p. 353-62.
39. Olson, M.F. and E. Sahai, *The actin cytoskeleton in cancer cell motility.* Clin Exp Metastasis, 2009. **26**(4): p. 273-87.
40. Safe, S. and A. McDougal, *Mechanism of action and development of selective aryl hydrocarbon receptor modulators for treatment of hormone-dependent cancers (Review).* Int J Oncol, 2002. **20**(6): p. 1123-8.
41. Tsay, J.J., et al., *Aryl hydrocarbon receptor and lung cancer.* Anticancer Res, 2013. **33**(4): p. 1247-56.
42. Sahai, E. and C.J. Marshall, *RHO–GTPases and cancer.* Nature Reviews Cancer, 2002. **2**(2): p. 133-142.
43. Shackelford, D.B. and R.J. Shaw, *The LKB1–AMPK pathway: metabolism and growth control in tumour suppression.* Nature Reviews Cancer, 2009. **9**(8): p. 563-575.
44. George, A.J., W.G. Thomas, and R.D. Hannan, *The renin-angiotensin system and cancer: old*

dog, new tricks. Nat Rev Cancer, 2010. **10**(11): p. 745-59.
45. Koeffler, H.P., F. McCormick, and C. Denny, *Molecular mechanisms of cancer.* West J Med, 1991. **155**(5): p. 505-14.
46. Sun, X., et al., *CXCL12/CXCR4/CXCR7 chemokine axis and cancer progression.* Cancer and Metastasis Reviews, 2010. **29**(4): p. 709-722.
47. Fang, J.Y. and B.C. Richardson, *The MAPK signalling pathways and colorectal cancer.* Lancet Oncol, 2005. **6**(5): p. 322-7.
48. Ross, J.S., et al., *The Her-2/neu gene and protein in breast cancer 2003: biomarker and target of therapy.* Oncologist, 2003. **8**(4): p. 307-25.
49. Dorsam, R.T. and J.S. Gutkind, *G-protein-coupled receptors and cancer.* Nat Rev Cancer, 2007. **7**(2): p. 79-94.
50. Cecchi, F., D.C. Rabe, and D.P. Bottaro, *Targeting the HGF/Met signalling pathway in cancer.* Eur J Cancer, 2010. **46**(7): p. 1260-70.
51. Feng, S., et al., *Relaxin promotes prostate cancer progression.* Clin Cancer Res, 2007. **13**(6): p. 1695-702.
52. Kamat, A.A., et al., *The role of relaxin in endometrial cancer.* Cancer Biol Ther, 2006. **5**(1): p. 71-7.
53. Kasthuri, R.S., M.B. Taubman, and N. Mackman, *Role of tissue factor in cancer.* J Clin Oncol, 2009. **27**(29): p. 4834-8.
54. Ruf, W. and B.M. Mueller, *Tissue factor in cancer angiogenesis and metastasis.* Curr Opin Hematol, 1996. **3**(5): p. 379-84.
55. Aviel-Ronen, S., et al., *K-ras mutations in non-small-cell lung carcinoma: a review.* Clinical lung cancer, 2006. **8**(1): p. 30-38.
56. Pao, W. and J. Chmielecki, *Rational, biologically based treatment of EGFR-mutant non-small-cell lung cancer.* Nature Reviews Cancer, 2010. **10**(11): p. 760-774.
57. Saretzki, G. and T. von Zglinicki, *Telomerase as a promising target for human cancer gene therapy.* Drugs Today (Barc), 2003. **39**(4): p. 265-76.
58. Saretzki, G., *Telomerase inhibition as cancer therapy.* Cancer Lett, 2003. **194**(2): p. 209-19.
59. Watkins, D.N., et al., *Hedgehog signalling within airway epithelial progenitors and in small-cell lung cancer.* Nature, 2003. **422**(6929): p. 313-7.
60. Sriuranpong, V., et al., *Notch signaling induces cell cycle arrest in small cell lung cancer cells.* Cancer Res, 2001. **61**(7): p. 3200-5.
61. Ellisen, L.W. and D.A. Haber, *Hereditary breast cancer.* Annu Rev Med, 1998. **49**: p. 425-36.
62. Lazennec, G. and A. Richmond, *Chemokines and chemokine receptors: new insights into cancer-related inflammation.* Trends Mol Med, 2010. **16**(3): p. 133-44.
63. Karin, M., et al., *NF-kappaB in cancer: from innocent bystander to major culprit.* Nat Rev Cancer, 2002. **2**(4): p. 301-10.
64. Bardeesy, N. and R.A. DePinho, *Pancreatic cancer biology and genetics.* Nat Rev Cancer, 2002. **2**(12): p. 897-909.
65. Dolle, L., et al., *Nerve growth factor receptors and signaling in breast cancer.* Curr Cancer Drug Targets, 2004. **4**(6): p. 463-70.
66. Zhu, Z.W., et al., *Nerve growth factor exerts differential effects on the growth of human pancreatic cancer cells.* Clin Cancer Res, 2001. **7**(1): p. 105-12.
67. Morad, S.A. and M.C. Cabot, *Ceramide-orchestrated signalling in cancer cells.* Nat Rev Cancer,

2013. **13**(1): p. 51-65.
68. Hynes, N.E. and H.A. Lane, *ERBB receptors and cancer: the complexity of targeted inhibitors.* Nat Rev Cancer, 2005. **5**(5): p. 341-54.
69. Gabarra-Niecko, V., M.D. Schaller, and J.M. Dunty, *FAK regulates biological processes important for the pathogenesis of cancer.* Cancer Metastasis Rev, 2003. **22**(4): p. 359-74.
70. Persad, S. and S. Dedhar, *The role of integrin-linked kinase (ILK) in cancer progression.* Cancer Metastasis Rev, 2003. **22**(4): p. 375-84.
71. Hermeking, H., *The 14-3-3 cancer connection.* Nat Rev Cancer, 2003. **3**(12): p. 931-43.
72. Reubi, J.C., et al., *Somatostatin receptors in human cancer: incidence, characteristics, functional correlates and clinical implications.* J Steroid Biochem Mol Biol, 1992. **43**(1-3): p. 27-35.
73. Sabatini, D.M., *mTOR and cancer: insights into a complex relationship.* Nature Reviews Cancer, 2006. **6**(9): p. 729-734.
74. Thiery, J.P., *Epithelial–mesenchymal transitions in tumour progression.* Nature Reviews Cancer, 2002. **2**(6): p. 442-454.
75. Waugh, D.J. and C. Wilson, *The interleukin-8 pathway in cancer.* Clin Cancer Res, 2008. **14**(21): p. 6735-41.
76. Xie, K., *Interleukin-8 and human cancer biology.* Cytokine & growth factor reviews, 2001. **12**(4): p. 375-391.
77. Hollander, M.C., G.M. Blumenthal, and P.A. Dennis, *PTEN loss in the continuum of common cancers, rare syndromes and mouse models.* Nature Reviews Cancer, 2011. **11**(4): p. 289-301.
78. Tamura, M., et al., *PTEN gene and integrin signaling in cancer.* J Natl Cancer Inst, 1999. **91**(21): p. 1820-8.
79. Holder, J.W., E. Elmore, and J.C. Barrett, *Gap junction function and cancer.* Cancer Res, 1993. **53**(15): p. 3475-85.
80. Cronier, L., et al., *Gap junctions and cancer: new functions for an old story.* Antioxid Redox Signal, 2009. **11**(2): p. 323-38.
81. Ellis, L.M. and D.J. Hicklin, *VEGF-targeted therapy: mechanisms of anti-tumour activity.* Nature reviews cancer, 2008. **8**(8): p. 579-591.
82. Germain, D., *Estrogen carcinogenesis in breast cancer.* Endocrinol Metab Clin North Am, 2011. **40**(3): p. 473-84, vii.
83. Yager, J.D. and N.E. Davidson, *Estrogen carcinogenesis in breast cancer.* N Engl J Med, 2006. **354**(3): p. 270-82.
84. Biankin, A.V., et al., *Pancreatic cancer genomes reveal aberrations in axon guidance pathway genes.* Nature, 2012. **491**(7424): p. 399-405.
85. Chedotal, A., G. Kerjan, and C. Moreau-Fauvarque, *The brain within the tumor: new roles for axon guidance molecules in cancers.* Cell Death Differ, 2005. **12**(8): p. 1044-56.
86. Saharinen, P., et al., *VEGF and angiopoietin signaling in tumor angiogenesis and metastasis.* Trends Mol Med, 2011. **17**(7): p. 347-62.
87. Huse, J.T. and E.C. Holland, *Targeting brain cancer: advances in the molecular pathology of malignant glioma and medulloblastoma.* Nature reviews cancer, 2010. **10**(5): p. 319-331.
88. Waldmann, T.A., *The biology of interleukin-2 and interleukin-15: implications for cancer therapy and vaccine design.* Nat Rev Immunol, 2006. **6**(8): p. 595-601.
89. Gulhati, P., et al., *mTORC1 and mTORC2 regulate EMT, motility, and metastasis of colorectal*

cancer via RhoA and Rac1 signaling pathways. Cancer Res, 2011. **71**(9): p. 3246-56.

90. Sonoshita, M., et al., *Suppression of colon cancer metastasis by Aes through inhibition of Notch signaling.* Cancer Cell, 2011. **19**(1): p. 125-37.
91. Mohyeldin, A., et al., *Erythropoietin signaling promotes invasiveness of human head and neck squamous cell carcinoma.* Neoplasia, 2005. **7**(5): p. 537-43.
92. Hardee, M.E., et al., *Erythropoietin biology in cancer.* Clin Cancer Res, 2006. **12**(2): p. 332-9.
93. Emons, G., et al., *GnRH antagonists in the treatment of gynecological and breast cancers.* Endocr Relat Cancer, 2003. **10**(2): p. 291-9.
94. Grundker, C. and G. Emons, *Role of gonadotropin-releasing hormone (GnRH) in ovarian cancer.* Reprod Biol Endocrinol, 2003. **1**: p. 65.
95. Wu, H., V. Goel, and F.G. Haluska, *PTEN signaling pathways in melanoma.* Oncogene, 2003. **22**(20): p. 3113-22.
96. Smalley, K.S., *Understanding melanoma signaling networks as the basis for molecular targeted therapy.* J Invest Dermatol, 2010. **130**(1): p. 28-37.
97. Belfiore, A. and F. Frasca, *IGF and insulin receptor signaling in breast cancer.* J Mammary Gland Biol Neoplasia, 2008. **13**(4): p. 381-406.
98. Baserga, R., F. Peruzzi, and K. Reiss, *The IGF-1 receptor in cancer biology.* Int J Cancer, 2003. **107**(6): p. 873-7.
99. Cohen, H.T. and F.J. McGovern, *Renal-cell carcinoma.* N Engl J Med, 2005. **353**(23): p. 2477-90.
100. Bast, R.C., Jr., B. Hennessy, and G.B. Mills, *The biology of ovarian cancer: new opportunities for translation.* Nat Rev Cancer, 2009. **9**(6): p. 415-28.
101. Lai, S.Y., et al., *Erythropoietin-mediated activation of JAK-STAT signaling contributes to cellular invasion in head and neck squamous cell carcinoma.* Oncogene, 2005. **24**(27): p. 4442-9.
102. Cerwenka, A. and L.L. Lanier, *Natural killer cells, viruses and cancer.* Nat Rev Immunol, 2001. **1**(1): p. 41-9.
103. Smyth, M.J., et al., *New aspects of natural-killer-cell surveillance and therapy of cancer.* Nat Rev Cancer, 2002. **2**(11): p. 850-61.
104. Pyne, N.J., et al., *Sphingosine 1-phosphate signalling in cancer.* Biochem Soc Trans, 2012. **40**(1): p. 94-100.
105. Feldman, B.J. and D. Feldman, *The development of androgen-independent prostate cancer.* Nat Rev Cancer, 2001. **1**(1): p. 34-45.
106. Wolf, M.J., et al., *The unexpected role of lymphotoxin beta receptor signaling in carcinogenesis: from lymphoid tissue formation to liver and prostate cancer development.* Oncogene, 2010. **29**(36): p. 5006-18.
